# OsbZIP81, A Homologue of Arabidopsis VIP1, May Positively Regulate JA Levels by Directly Targetting the Genes in JA Signaling and Metabolism Pathway in Rice

**DOI:** 10.3390/ijms20092360

**Published:** 2019-05-13

**Authors:** Defang Liu, Shaopeng Shi, Zhijun Hao, Wentao Xiong, Meizhong Luo

**Affiliations:** College of Life Science and Technology, Huazhong Agricultural University, Wuhan 430070, China; liudefang@webmail.hzau.edu.cn (D.L.); ssp15212781108@163.com (S.S.); haozj1225@163.com (Z.H.); xiongwentao860820@163.com (W.X.)

**Keywords:** OsbZIP81, VirE2, OVRE, OsPIOX, JA signaling and metabolism pathway, JA levels, PR proteins

## Abstract

Rice (*Oryza sativa* L.) is one of the most important food crops in the world. In plants, jasmonic acid (JA) plays essential roles in response to biotic and abiotic stresses. As one of the largest transcription factors (TFs), basic region/leucine zipper motif (bZIP) TFs play pivotal roles through the whole life of plant growth. However, the relationship between JA and bZIP TFs were rarely reported, especially in rice. In this study, we found two rice homologues of Arabidopsis VIP1 (VirE2-interacting protein 1), *OsbZIP81*, and *OsbZIP84*. *OsbZIP81* has at least two alternative transcripts, *OsbZIP81.1* and *OsbZIP81.2*. OsbZIP81.1 and OsbZIP84 are typical bZIP TFs, while OsbZIP81.2 is not. OsbZIP81.1 can directly bind *OsPIOX* and activate its expression. In OsbZIP81.1 overexpression transgenic rice plant, JA (Jasmonic Acid) and SA (Salicylic acid) were up-regulated, while ABA (Abscisic acid) was down-regulated. Moreover, *Agrobacterium*, Methyl Jasmonic Acid (MeJA), and PEG6000 can largely induce *OsbZIP81*. Based on ChIP-Seq and Random DNA Binding Selection Assay (RDSA), we identified a novel *cis*-element OVRE (Oryza VIP1 response element). Combining ChIP-Seq and RNA-Seq, we obtained 1332 targeted genes that were categorized in biotic and abiotic responses, including α-linolenic acid metabolism and fatty acid degradation. Together, these results suggest that OsbZIP81 may positively regulate JA levels by directly targeting the genes in JA signaling and metabolism pathway in rice.

## 1. Introduction

Rice (*Oryza sativa* L.) is a model species for monocotyledonous plants and cereals, which are the greatest source of food for the world’s population. With the great change in climate, rice is confronted with critical biotic and abiotic stresses. Plants have evolved complex signaling pathways to survive under multiple stresses, which are generally composed of receptors, secondary messengers, phytohormones, and signal transducers [1]. Plant hormones are fundamentally involved in plant growth and development, and they play roles in adapting to the changing environment and in quick responses to multiple stresses. Jasmonic acid (JA), as a basic plant growth regulator, is widely present in higher plants and a natural compound that is produced in plants [2,3,4,5]. As an important endogenous hormone, JA plays diverse roles in plant growth, seed germination, drought stress, pathogens, and insects’ defenses [3,6,7,8,9,10,11].

Transcription factors (TFs) are triggers for gene expression and they play important regulatory roles throughout the plant life time, especially in plant growth, development, and responses to abiotic and biotic stresses. Being one of the largest families of transcriptional regulators, the basic region/leucine zipper motif (bZIP) transcription factors have been systematically characterized in many higher plants. There are 75 [12] or 78 bZIP TFs in Arabidopsis [13], 89 [14] or 92 in rice [15], 125 in maize [16], and 247 in rapeseed [17]. These bZIP TFs have been classified into 13 groups in Arabidopsis (A, B, C, D, E, F, G, H, I, J, K, M, S) [13] and 11 in rice (I–XI),according to the DNA binding specificity and amino acid sequence similarities of bZIP domains [14]. In Arabidopsis, group I is the subfamily with the third largest number of genes among all of the groups, containing 12 members [13]. Among the 12 members, VIP1/AtbZIP51 is a VirE2-interacting protein and it has been well studied [18]. It was thought to play an important role in *Agrobacterium*-mediated T-DNA transfer [18]. In the model of the “Trojan house hypothesis”, VIP1 serves as a bridge between VirE2 and nuclear importin α, which mediates the transport of the T-DNA strand to the plant nucleus [19,20]. Moreover, the subcellular localization of VIP1 is affected by its own phosphorylation status and interaction with 14-3-3 [21]. VIP1 is involved in other functions in addition to its role in *Agrobacterium*-mediated transformation, including osmosensory signaling, low sulfur tolerance, metal-binding, touch response, *Botrytis* and salt stress responses, the ABA response, and transcriptional regulation [22,23,24,25,26,27,28,29,30]. VIP1 can bind to VRE (VIP1 response element: ACNGCT) or VRE similar sequences (AGCTGT/G, CAGCT) of promoters and control the expression of stress-related genes [23,24,26,31,32]. In addition to VIP1, numerous members of the group I subfamily can interact with C58 VirE2 (AtbZIP52, AtbZIP69, PosF21/AtbZIP59, AtbZIP29, and AtbZIP30) and they are involved in osmosensory responses (PosF21/AtbZIP59, AtbZIP69, AtbZIP29, AtbZIP30, and AtbZIP52) and vascular development (AtbZIP18, AtbZIP29, AtbZIP30, AtbZIP52, PosF21/AtbZIP59, and AtbZIP69), revealing the functional redundancy among group I members [26,33,34]. Moreover, AtbZIP29 has been defined to function in leaf and root development, PosF21/AtbZIP59 in auxin-induced callus formation and plant regeneration, DRINK ME/AtbZIP30 in growth and reproductive development regulation, and AtbZIP18 in pollen and male gametophyte development [6,27,35,36].

Group IX (or B) of bZIPs in rice remains poorly described. This group represents the fourth largest subfamily, is very close to group I in Arabidopsis, and contains 11 members (OsbZIP25, OsbZIP30, OsbZIP35, OsbZIP36 OsbZIP61, OsbZIP68, OsbZIP75, OsbZIP76, OsbZIP78, OsbZIP81, and OsbZIP84); however, only two of them have been systematically studied [37,38]. RF2a/OsbZIP75 functions in rice vascular development and they can bind to the Box II *cis*-element of the promoter of rice tungro bacilliform virus (RTBV) to activate its expression [37,39]. RF2b/OsbZIP30 can interact with RF2a and it is involved in the symptom development of rice tungro disease and vascular development [38]. Transgenic rice plants overexpressing RF2a and RF2b present a tolerance to rice tungro virus replication and disease [40].

*Agrobacterium* (*Agrobacterium tumefaciens*) is a kind of soil bacterium and a pathogen. It is also a natural genetic engineer, which plays prominent roles in transferring genetic information into the eukaryotic genome [41]. In our previous studies, we found that *Agrobacterium* VirD5 could interact with Arabidopsis VIP1 and competitively inhibit the interaction between VIP1 and VBF. This competitive interaction could prevent T-DNA coat protein degradation in the plant cell nucleus [42]. In addition, VirD5 could increase the *Agrobacterium* infection efficiency, potentially by a competitive interaction with Arabidopsis VIP2 [41]. In another work, our group demonstrated that a large maize fragment (164 kb) that contained a high content of repetitive sequences was successfully transferred into rice by *Agrobacterium*-mediated transformation, but the transformation efficiency was very low [43]. Thus, the application of large exogenous DNA fragments in rice is largely limited. Therefore, the elucidation of the mechanism underlying *Agrobacterium*-mediated rice transformation may help to solve the problem.

In this study, we identified two rice homologues of the Arabidopsis *VIP1* gene, *OsbZIP81* and *OsbZIP84*, and functionally analyzed OsbZIP81. OsbZIP81 and OsbZIP84 both belong to group IX of bZIPs. OsbZIP81.1 and OsbZIP84 are typical transcription factors of the bZIP super family. OsbZIP81.1 may positively affect the JA levels of rice plant by directly targeting the genes in JA signaling and metabolism pathway, especially OsPIOX. In addition, OsbZIP81.2 can interact with *Agrobacterium* VirE2. To our knowledge, this is the first report on the interaction between rice proteins and *Agrobacterium* virulence proteins. Furthermore, we identified two pathogenesis-regulated (PR) proteins, PR10a/PBZ1 and RSOsPR10, and other stress response genes. These results suggest that OsbZIP81 may positive regulate JA levels and may play a role in pathogen resistance.

## 2. Results

### 2.1. Identification of Arabidopsis VIP1 Homologue(s) in Rice

**Figure 1:**
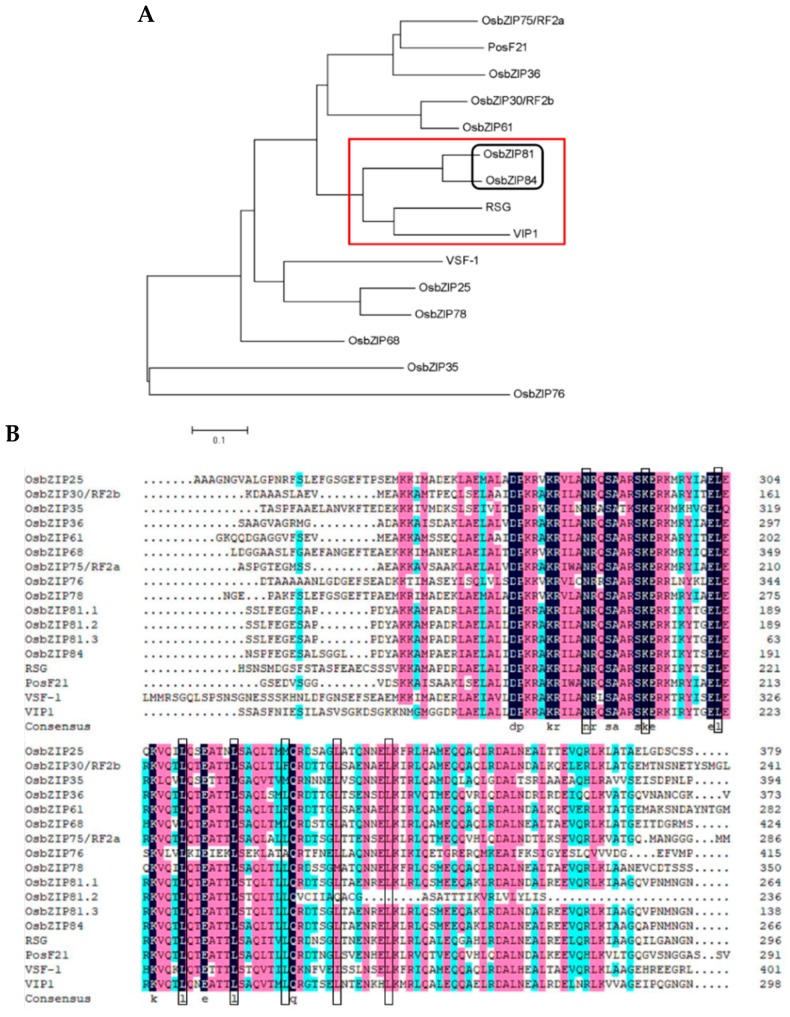
Evolutionary relationship of OsbZIP81 homologs and alignment of basic region/leucine zipper motif (bZIP) domains. (**A**) The evolutionary relationship of OsbZIP81 and its homologs, including the same subfamily members from rice, VIP1 and PosF21 from Arabidopsis, RSG from tobacco and VSF-1 from tomato. The evolutionary history was inferred using the maximum likelihood method. The software ClustalX (v. 1.83) and MEGA7 were used. The closest evolutionary relationship of OsbZIP81 were circled with different colored boxes (black box and red box). (**B**) Alignment of the bZIP domain of OsbZIP81 homologues.

We used the Arabidopsis *VIP1* as a query to search similar sequences in rice by mining the RGAP database with the BLAST program to understand the mechanism of *Agrobacterium*-mediated rice transformation in rice. Two potential homologous genes were found: GenBank accession numbers: XM_015762292 (*OsbZIP81*) and XM_015762716 (*OsbZIP84*). *OsbZIP81* (LOC_Os11g06170) and *OsbZIP84* (LOC_Os12g06520) are members of the bZIP transcription factor family. They both belong to the group IX subfamily. This group contains 11 genes but it encodes 13 proteins. Genes that have been reported to belong to the same subfamily are *VIP1* and *PosF21* in Arabidopsis, *RSG* in tobacco and *VSF-1* in tomato [18,44,45,46]. Based on an analysis of the evolutionary relationship of all group IX subfamily members, we found that OsbZIP81 had the closest evolutionary relationship with OsbZIP84 (Figure 1A, black box), followed by VIP1 and RSG (Figure 1A, red box). Through a comparison of the bZIP conserved domains of these genes, we found that the number of leucine in the leucine zipper region of the bZIP domain ranged from three to six, and the amino acids at positions -10 and -18 before the first leucine were K and N, respectively (Figure 1B, black box). These results indicate that the members of the group IX subfamily in different plants have highly conserved protein sequences, and that OsbZIP81 and OsbZIP84 are homologues of Arabidopsis VIP1 in rice.

**Figure 2:**
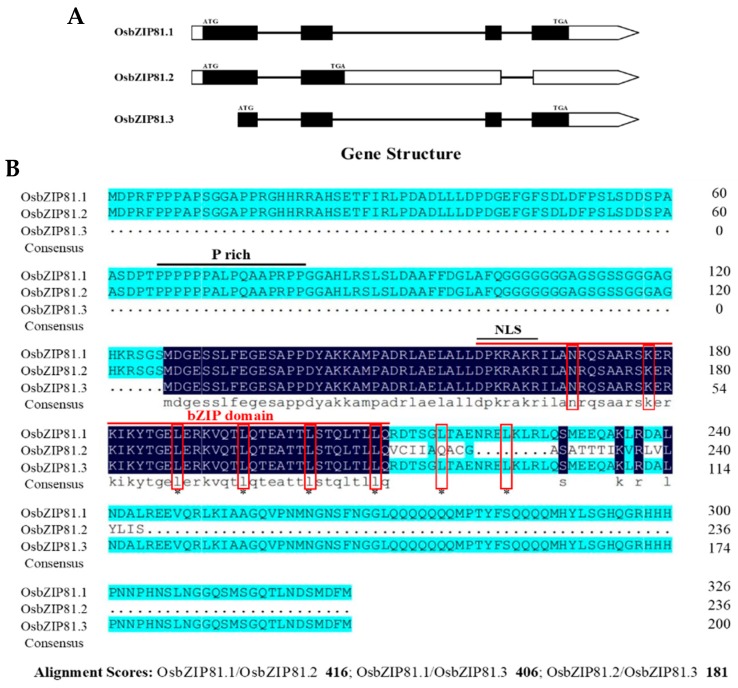
Gene structure and sequence alignment of *OsbZIP81* variants. (**A**) Schematic representation of the *OsbZIP81* genomic organization with UTRs (white boxes), exons (black boxes) and introns (lines between exons). (**B**) Alignment of the amino acid sequences of the OsbZIP81 variants. The conserved properties or sites were circled by red boxes.

By applying a general bioinformatics analysis, we found that *OsbZIP81* had three alternative transcripts of 981, 711 and 603 bp (GenBank Accession NO. XP_015617778, BAT12791, and BAF27663.1). The three transcripts encoded 326, 236 and 200 amino acids (from long to short), and they were designated *OsbZIP81.1*, *OsbZIP81.2*, *OsbZIP81.3*, respectively. OsbZIP84 had only one transcript (encoding 328 amino acids). The three variants (CDS) of *OsbZIP81* were successfully isolated from young seedlings of rice by reverse transcription PCR (RT-PCR). OsbZIP81.1 and OsbZIP81.2 shared amino acids from 1 to 210, while OsbZIP81.3 was part of OsbZIP81.1 lacking the N-terminal 126 amino acids (Figure 2). Due to the difficulty confirming the existence of *OsbZIP81.3*, we focused our study on the first two variants, *OsbZIP81.1* and *OsbZIP81.2*.

### 2.2. Subcellular Localization of OsbZIP81 and OsbZIP84

**Figure 3:**
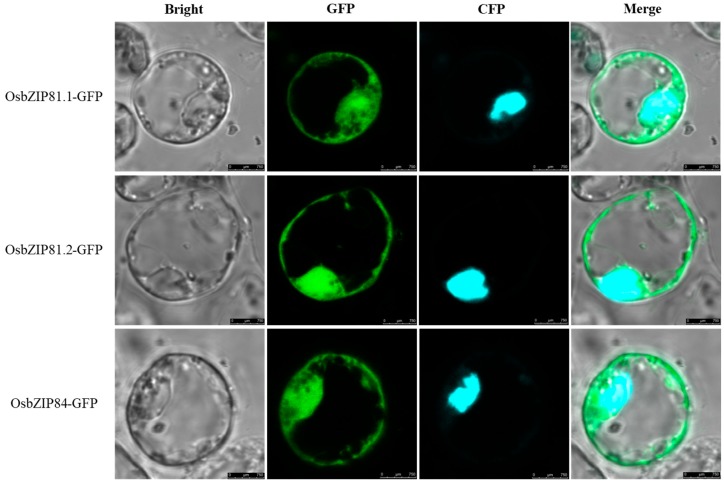
Subcellular localization of OsbZIP81.1, OsbZIP81.2, and OsbZIP84 proteins. Subcellular localization of OsbZIP81.1, OsbZIP81.2, and OsbZIP84 were determined in rice protoplasts. 35S::CFP-Ghd7 was used as the nucleic marker [48]. The confocal image was acquired using a confocal laser scanning microscope.

The subcellular localization of a protein is an important clue in understanding its function. OsbZIP81.1, OsbZIP81.2, and OsbZIP84 were all predicted to be localized in the nucleus by ProtComp 9.0 (www.softberry.com) and Cell-PLoc 2.0 [47], two online programs that are used to predict the subcellular localization of plant proteins. To experimentally confirm the subcellular locations, we constructed the vectors pM999-OsbZIP81.1-GFP, pM999-OsbZIP81.2-GFP, and pM999-OsbZIP84-GFP by fusing GFP to the C-terminus of OsbZIP81.1, OsbZIP81.2, and OsbZIP84, respectively. We observed that the GFP signals of OsbZIP81.1-GFP, OsbZIP81.2-GFP, and OsbZIP84-GFP were localized in the nucleus and cytoplasm (Figure 3).

### 2.3. OsbZIP81.1 and OsbZIP84 Have Strong Self-Activation and Transactivation Activities

**Figure 4:**
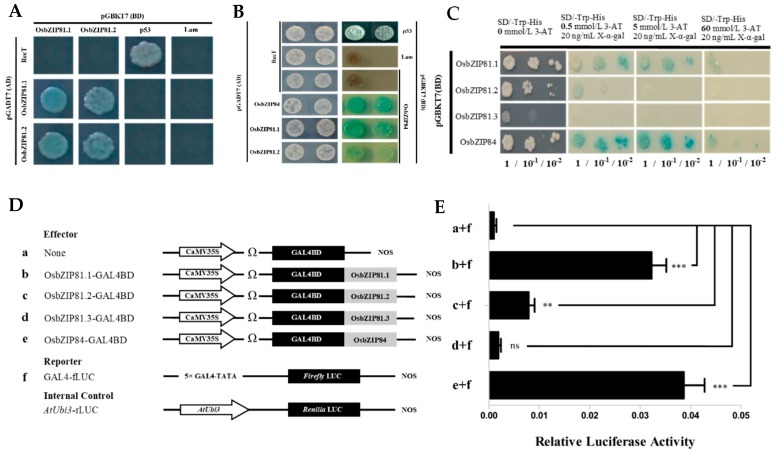
The self-activating activity test of the proteins encoded by *OsbZIP81* and *OsbZIP84*. (**A**) and (**B**) Yeast two hybrid experiments for dimer detection among OsbZIP81.1, OsbZIP81.2, and OsbZIP84. (**C**) Self-activation activity assay. The concentrations of 3-AT were set as 0, 0.5, 1, 3, 5, 10, 15, 30, 60, and 120 mmol/L, and this figure only shows partial results. (**D**) Schematic of the vectors used in the rice protoplast co-transformation assay. (**E**) Transcription activities of *OsbZIP81* and *OsbZIP84*. The activity of GAL4-Flirfly luciferase (fLUC) was used as the reporter and that of Renilla luciferase (rLUC) was used as an internal control. The fLUC/rLUC ratio represents the relative activity of the gene. The values in each column are the means of at least three independent replicates and error bars represent the SEM. The asterisks represent a significant difference determined by the Student’s t test, triple asterisks indicate *p*-value < 0.001, the double asterisks indicate *p*-value < 0.01, and ns indicates *p*-value > 0.05.

Transcriptional activity, dimer formation, and nuclear localization are the key functional characteristics of a bZIP TF. Rice protoplast transformation and yeast two-hybrid (Y2H) experiments were performed to ascertain whether OsbZIP81.1, OsbZIP81.2, and OsbZIP84 functioned as TFs. Based on the two experiments, we found that OsbZIP81.1, OsbZIP81.2, and OsbZIP84 could form homodimers with themselves and heterodimers with each other (Figure 4A,B). Additionally, OsbZIP81.1 and OsbZIP84 showed very strong self-activation and transactivation activities, while OsbZIP81.2 had little and OsbZIP81.3 had no activation activity when compared with the control (Figure 4C–E). Together with the subcellular localization results, these findings indicated that OsbZIP81 and OsbZIP84 could play roles as transcription factors.

### 2.4. OsbZIP81.1 and OsbZIP84 Can Bind to the Motif Containing GCTG-Core Sequence

**Figure 5:**
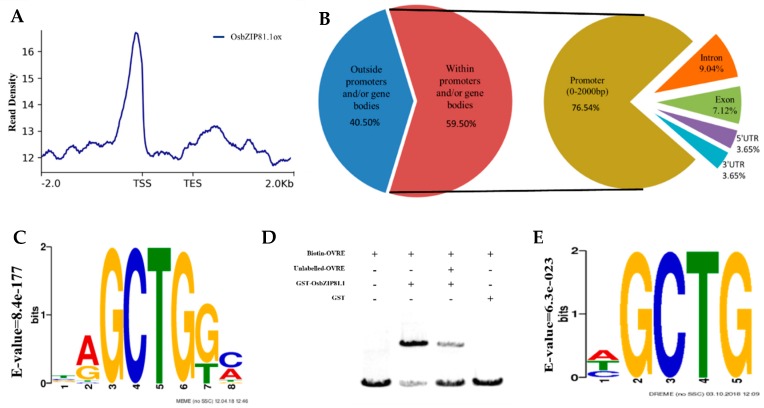
ChIP-Seq and Random DNA Binding Selection Assay (RDSA) data analyses. (**A**) Distribution of OsbZIP81.1 binding sites in the genic regions of the rice genome. (**B**) Statistics of distribution of OsbZIP81.1 binding sites in the rice genome. (**C**) Information regarding the most significant motif identified in the OsbZIP81.1 binding peaks with Multiple EM for Motif Elicitation (MEME). (**D**) Electrophoretic mobility shift assay (EMSA) assay. Glutathione S-Transferase (GST) tagged OsbZIP81.1 protein and biotin-labeled, or unlabeled TAGCTGGC sequences were used in the experiment. The concentration of unlabeled sequence was 200 times that of the biotin-labeled sequence. (**E**) Information regarding the most significant motif identified in the RDSA experiment.

ChIP-Seq and Random DNA Binding Selection Assay (RDSA) assays were performed for OsbZIP81.1 and OsbZIP84 to identify the sequences that were bound by OsbZIP81.1 and OsbZIP84, respectively. For the ChIP-Seq assay, a specific antibody against OsbZIP81.1-flag tag was used to pull down the putative OsbZIP81.1-bound DNA sequences from the seedlings of OsbZIP81.1ox transgenic rice (see Section 2.7). The average fragment sizes of the input and anti-flag ChIP libraries were 365 and 362 bp, respectively. After sequencing, we obtained 34,501,154 uniquely mapped reads for input and 43,192,879 reads for IP. More than 96% of the reads were mapped to *Oryza sativa* (RGAP v7.0) (Table 1). The unique mapped reads were mainly located within 500 bp upstream of the transcription start site (TSS) (Figure 5A). The Model-based Analysis of ChIP-Seq (MACS) program was used to identify the enriched regions using a false discovery cut-off of 0.05. Finally, we identified 14,245 peaks (Supplementary File S1, Supplementary Figure S1) that represented 8173 genes (Supplementary Files S2 and S3). In the enriched peaks, 59.5% were in genic regions (from 2 kb upstream of the transcription start site to 2 kb downstream of the stop codon) (Figure 5B). Of the peaks in genic regions, 76.54% were in promoter regions, 9.04% were in introns, 7.12% were in exons, 3.65% were in 5′UTR regions, and 3.65% were in 3′UTR regions (Figure 5B). Table 2 presents the genes that were associated with peaks (in genic regions, enriched greater than 13-fold and located at 500 bp upstream of CDS) and known putative functions.

**Figure 6:**
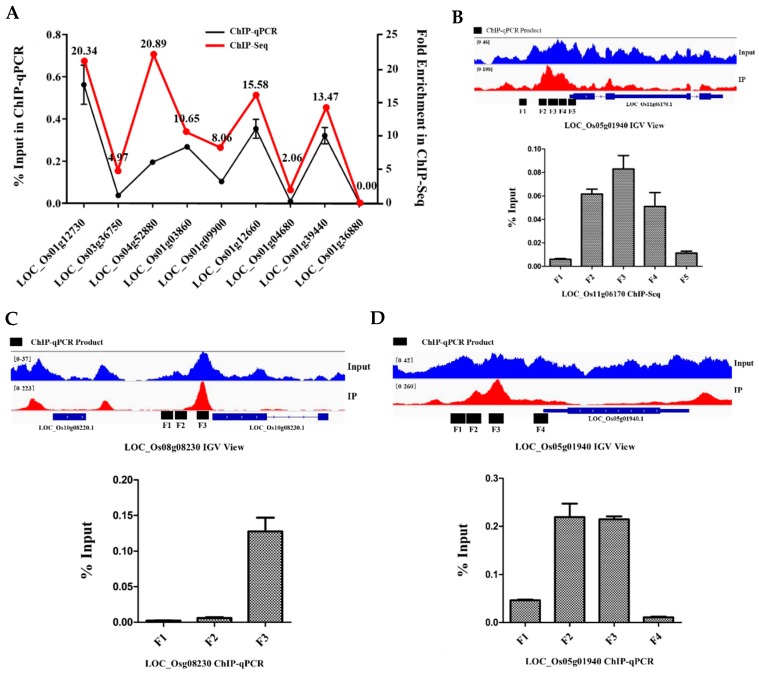
Verification of the ChIP-Seq results by ChIP-quantitative PCR (ChIP-qPCR). (**A**) Verification of the ChIP-Seq results by ChIP-qPCR including eight binding sites and one unbinding site (belonged to *LOC_Os12g36880*). (**B**,**D**) Verification of the ChIP-Seq results by ChIP-qPCR including 12 sites that belonged to three genes. The 12 sites at the promoter region of the three genes were arranged below the histogram. The primer pairs used in the RT-qPCR assay are represented with black lines in the promoter region (black boxes from left to right are F1 to F5 for *LOC_Os11g06170*, F1 to F3 for *LOC_Os10g08230*, and F1 to F4 for *LOC_Os05g01940*). The values in each column are the means of three independent replicates and error bars represent the SEM.

ChIP-quantitative PCR (ChIP-qPCR) was performed for 12 randomly selected genes, including 11 target genes and one nontarget gene to validate the ChIP-Seq results. Of the 12 genes, three were determined to have 3–5 potential binding sites in the promoter region. In general, the ChIP-qPCR values agreed with the ChIP-Seq results (Figure 6). For OsbZIP81.2, only 615 peaks in total were identified and 77 peaks were significantly enriched (fold enrichment > 2.5, *p*-value < 0.05). The maximum fold enriched only reached 5.13 (Supplementary File S4). When combining the above results, we suggest that the primary role of OsbZIP81.2 may not be as a transcription factor.

A motif search was performed using the most commonly used Multiple EM for Motif Elicitation (MEME) software [49]. The motif analysis presented 15 motifs (Supplementary Table S1). The most significantly enriched motif was Motif 1: core sequence GCTG (E-value of 8.4 × 10^−177^). Approximately 80.5% of the highest 1000 *p*-value peaks contained this motif and they were evenly distributed among all of the chromosomes. We renamed this motif OVRE for *Oryza* VIP1 response element (Figure 5C). The electrophoretic mobility shift assay (EMSA) further validated that OsbZIP81.1 could bind the motif OVRE (Figure 5D).

The random DNA binding selection assay (RDSA) is another way to identify the sequence(s) bound by transcription factors, which is a way to investigate the protein-DNA interaction in vitro. The purified OsbZIP84 protein (fused to a GST-tag) and randomly synthetized oligonucleotides were used in this assay. We obtained 349 unique sequences after enrichment and sequencing. These sequences were submitted to the MEME website to predict the motif(s). Finally, we obtained three significantly enriched motifs from the DREME results, and the highest one was consistent with OVRE (Figure 5E, Supplementary Table S2), demonstrating that OsbZIP81.1 and OsbZIP84 are functionally similar as the transcription factors.

### 2.5. OsbZIP81.2 Can Interact with VirE2

**Figure 7:**
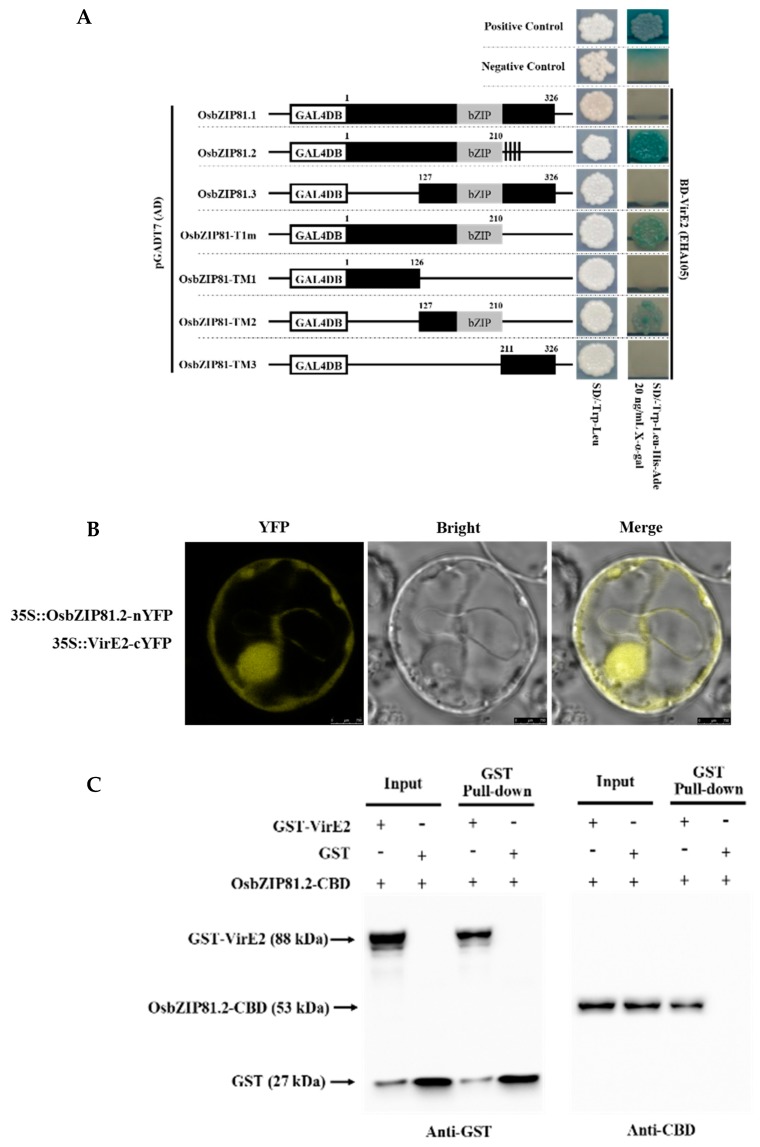
OsbZIP81.2 can interact with virulence effector protein 2 (VirE2). (**A**) Different truncated proteins of OsbZIP81 and VirE2 in yeast two-hybrid assay. (**B**) OsbZIP81.2 interacts with VirE2 in a BiFC assay. (**C**) OsbZIP81.2 interacts with VirE2 in a GST pull-down assay.

Previous reports have shown that Arabidopsis VIP1 and certain members of the same group can interact with the *Agrobacterium* protein VirE2 [18,34]. We cloned the three types of VirE2 from agropine (EHA105), nopaline (GV3101), and octopine (LBA4404) *Agrobacterium*, and all 13 members of group IX bZIPs from rice to assess whether the rice VIP1 homologues could also interact with different types of VirE2. Through the Y2H assay, we found that only OsbZIP81.2 could interact with three types of VirE2 in yeast (Figure 7A, Supplementary Figure S2), and this interaction was confirmed by BiFC and VirE2 GST pull-down (Figure 7B,C). This result suggested that OsbZIP81.2 might be conserved for some functions of VIP1 and might be involved in *Agrobacterium*-mediated transformation.

### 2.6. OsbZIP81 Can Be Strongly Induced by Agrobacterium, Methyl Jasmonic Acid (MeJA), and PGE6000

**Figure 8:**
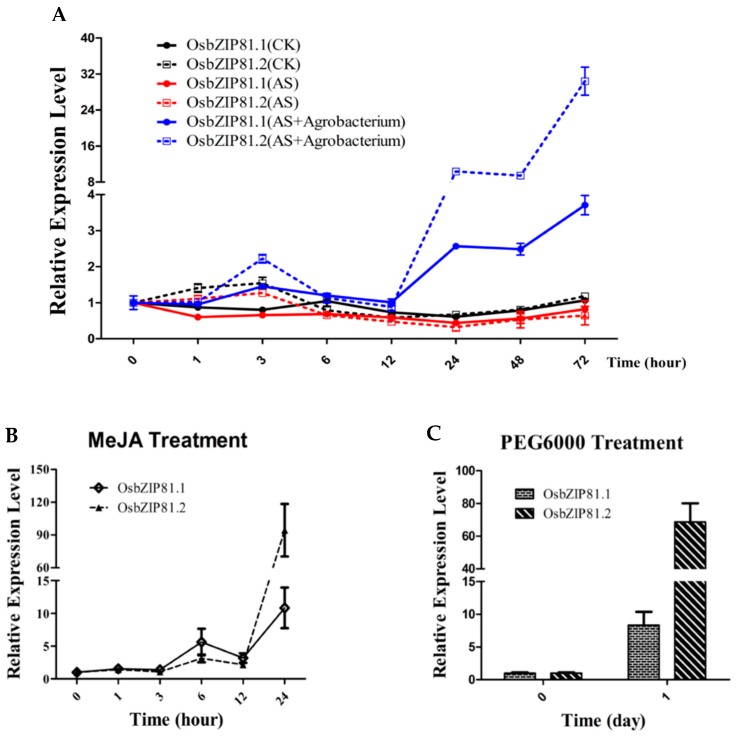
Expression of *OsbZIP81* under different biotic and abiotic stresses. (**A**) *Agrobacterium tumefaciens* infection of rice callus (Zhonghua 11, ZH11) with EHA105 strain and AS (100 μmol/L) treatment. Samples were collected after 0, 1, 3, 6, 12, 24, 48, and 72 h. (**B**) Methyl Jasmonic Acid (MeJA) (200 μmol/L) treatment. Samples were collected after 0, 1, 3, 6, 12, 24, 48, and 72 h. (**C**) PEG6000 (20%) treatment. Samples were harvested one day after treatment. The values in each column are the means of three independent replicates and error bars represent the SEM.

To investigate the physiological and functional relevance of the *OsbZIP81* gene, we checked its expression profile under different biotic and abiotic stresses by quantitative real-time PCR (qRT-PCR) assay. Overall, although the expression of *OsbZIP81.2* was much higher than that of *OsbZIP81.1* in most treatments, the trend was the same (Figure 8). The transcription levels of *OsbZIP81.1* and *OsbZIP81.2* were strongly induced by *Agrobacterium* infection and MeJA, PGE6000 treatments, and weakly induced by AS treatment (Figure 8A–C). The expression of *OsbZIP81.1* and *OsbZIP81.2* was mildly induced and reached a maximum at 6 h in response to ABA, SA, C_2_H_4_, NAA, IAA, and NaCl (Supplementary Figure S3B–G). Under heat/cold stress, *OsbZIP81.1* was sustainably induced by heat (42 °C) and *OsbZIP81.2* by cold (4 °C), with rising trends at 3 h after treatments (Supplementary Figure S3H,I). We also investigated whether the expression of *OsbZIP81* had any tissue specificity. The results indicated that the expression of both *OsbZIP81.1* and *OsbZIP81.2* were higher in leaves and flag leaves than in other tissues (Supplementary Figure S4).

### 2.7. Genome-Wide Identification of the Bound and Regulated Genes of OsbZIP81

We generated overexpression rice plants for the two transcripts, OsbZIP81.1-overexpression (OsbZIP81.1ox) and OsbZIP81.2-overexpression (OsbZIP81.2ox) to understand the potential function of OsbZIP81. We obtained 33 and 21 overexpression rice lines for *OsbZIP81.1* and *OsbZIP81.2*, respectively. We selected OsbZIP81.1ox-1, 3, 5 and OsbZIP81.2ox-2, 3, 4 for further studies based on the expression levels of the rice lines (Supplementary Figure S5).

For genome-wide analysis of the function of OsbZIP81, we performed an RNA-Seq assay. Nine RNA-Seq libraries were constructed with four-leaf stage seedlings: three for the OsbZIP81.1ox lines (OsbZIP81.1ox-1, 3, 5), three for the OsbZIP81.2ox lines (OsbZIP81.2ox-2, 3, 4), and three for wild-type ZH11 plants. Through high-throughput sequencing, we obtained 63 to 81 million reads. Most of these reads were mapped to the rice reference genome and transcriptome of *Oryza sativa* (RGAP v. 7) [50] (Supplementary Table S3). After analyzing the above data, we obtained 5143 (OsbZIP81.1ox_vs_ZH11) (Supplementary File S5) and 5002 (OsbZIP81.2ox_vs_ZH11) (Supplementary File S6) differentially expressed genes (DEGs) with parameters of |log_2_FC| > 1 and *p*-value < 0.05. The RNA-Seq results were validated by RT-qPCR of 57 randomly selected genes (belonging to seven different biological processes) (Supplementary Figure S6).

**Figure 9:**
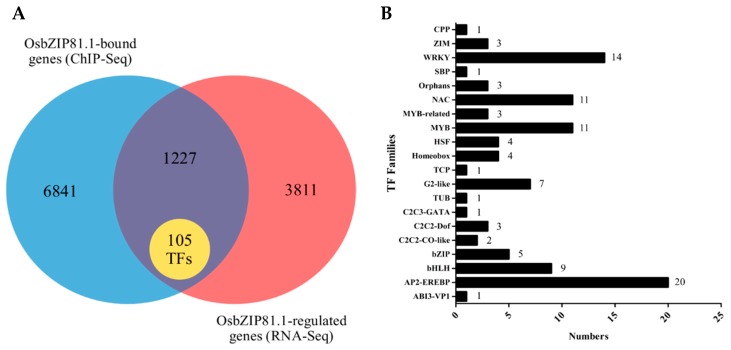
Venn analysis and statistics for OsbZIP81.1 targeted and regulated genes. (**A**) Venn diagram showing the number of genes regulated by OsbZIP81.1 based on the ChIP-Seq and RNA-Seq analyses. (**B**) One hundred and five overlapping genes belonged to 20 transcription factor families.

We identified 1332 target genes of OsbZIP81.1 (Supplementary File S7) when combining the results of ChIP-Seq and RNA-Seq, including 105 TFs belonging to 20 TF families (Figure 9, Supplementary File S8). To explore the potential function of OsbZIP81.1, the 1332 targeted genes were analyzed by Gene Ontology (GO) studies using the AgriGO online Gene Set Enrichment Analysis tool (http://bioinfo.cau.edu.cn/agriGO/index.php) [51]. The GO studies revealed 38 categories that belonged to Biological Process (P), 23 to Molecular Function (F) and 1 to Cellular Component (C). These categories were determined to be significantly overrepresented in the ChIP-Seq and RNA-Seq analysis (Supplementary File S9). We used the KEGG database to analyze the pathway annotations of the DEGs to characterize the complex biological behaviors of the transcriptome (OsbZIP81.1ox_vs_ZH11 and OsbZIP81.2ox_vs_ZH11). For OsbZIP81.1ox, 20 pathways were enriched with the most representation (*p*-value < 0.05) (Supplementary File S10). For OsbZIP81.2ox, 11 pathways were enriched with the most representation (Supplementary File S11).

### 2.8. OsbZIP81.1 May Positively Affects JA levels through Directly Targetting the Genes in JA Signaling and Metabolism Pathway

**Figure 10:**
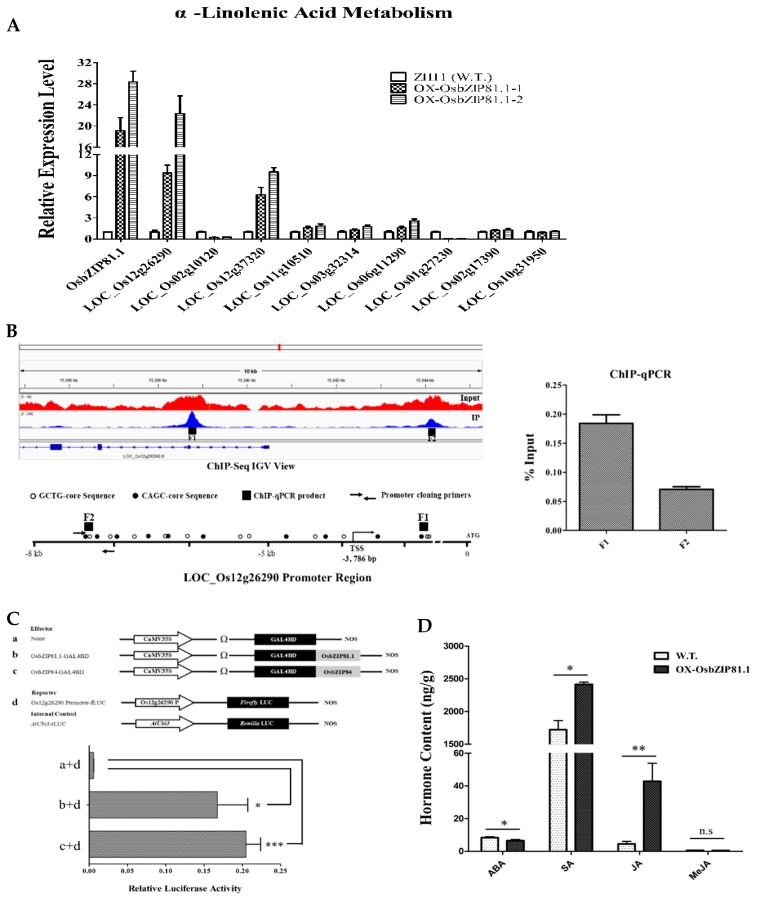
OsbZIP81.1 directly and positively regulates the expression of OsPIOX. (**A**) The expression level of genes enriched in α-linolenic acid metabolism pathway that were checked by RT-qPCR. (**B**) Peaks of OsPIOX in ChIP-Seq library and validation of the peaks by ChIP-qPCR. (**C**) Scheme of the constructs used in the rice protoplast cotransfection assay and the activities of different combinations were detected. The activity of GAL4-fLUC was used as the reporter and rLUC activity was used as an internal control. The fLUC/rLUC ratio represents the relative activity of the gene. The asterisks represent a significant difference determined by the Student’s *t* test, double asterisks indicate *p*-value < 0.01 and > 0.001, one asterisk indicates *p*-value < 0.05 and > 0.01, and n.s indicates *p*-value > 0.05. (**D**) Measurement of the ABA, SA, JA, and MeJA content in wild type Zhonghua 11 and OsbZIP81.1 overexpression transgenic rice plant (OX-OsbZIP81.1). The values in each column are the means of three independent replicates and error bars represent the SEM.

By carefully checking the genes identified in ChIP-Seq and RNA-Seq, we found that 9 genes that belong to α-linolenic acid metabolism pathway are bound and regulated by OsbZIP81.1 (Supplementary Files S1, S5 and S10). In the nine genes, five were up-regulated and two were down-regulated in OsbZIP81.1 overexpression transgenic rice plants. OsPIOX (LOC_Os12g26290) is one of the up-regulated genes, and its expression level reached to 3.77-fold (log_2_) in RNA-Seq data (Supplementary File S5). RT-qPCR confirmed this result (Figure 10A). By ChIP-Seq analysis, two distinctive peaks of this gene were detected, one at ~1.2 kb downstream of the TSS site, and the other one located at ~3.8 kb upstream of the TSS site (Figure 10B left). ChIP-qPCR verified that OsbZIP81.1 could specially binds to the two regions (Figure 10B right). The peaks for the other eight genes were also verified by ChIP-qPCR (Supplementary Figure S7). Furthermore, we selected approximately 500-bp sequence containing the second peaks (OsPIOX-F2) as a promoter to perform a dual-luciferase transient transcriptional activity assay. The OsPIOX promoter-driven reporter was up-regulated by both OsbZIP81.1 and OsbZIP84 (Figure 10C). To further understand whether OsbZIP81.1 could affect the JA levels, we measured the contents of JA, MeJA, SA, and ABA with OsbZIP81.1 overexpression and wild type rice plants. When compared with wild type plants, JA and SA were up-regulated in OsbZIP81.1ox, and ABA was a little down-regulated, while no significance was observed in MeJA (Figure 10D). Together, these results suggest that OsbZIP81.1 can directly and positively regulate the expression level of OsPIOX that may affect the levels of JA in rice.

### 2.9. OsbZIP81 can Interact with PR Proteins in Yeast

To understand the systematic function of OsbZIP81 in rice, we investigated the potential interacting proteins. By adopting the Y2H screening of a rice cDNA yeast library with OsbZIP81.2 (OsbZIP81.1 cannot be used as a bait in this experiment because of its strong self-activation activity), we obtained 154 clones in total, including 42 unique genes (proteins) (Supplementary Table S4). Most of these genes were involved in rice growth and development. Several other genes, LOC_Os12g36830 (RSOsPR10), LOC_Os12g36850 (PR10 family gene), LOC_Os12g36880 (OsPR10a/PBZ1), LOC_Os11g05860, and LOC_Os06g22919 (DEFL family gene), were involved in plant defense. The interactions between OsbZIP81.2 and RSOsPR10, and between OsbZIP81.2 and OsPR10a/PBZ1, were further confirmed by Y2H (Supplementary Figure S8). For OsbZIP81.1, we found that it could only interact with RSOsPR10, but not with OsPR10a/PBZ1 (Supplementary Figure S8).

## 3. Discussion

### 3.1. OsbZIP81 is an AS Gene

Alternative splicing (AS) is a critical feature of post-transcription in eukaryotes that can both increase protein diversity and function as an additional regulatory point of gene expression. At the level of the proteome, alternative splicing may generate a tremendous diversity to adapt to the demands of plant development and a stressful environment [52,53,54,55]. In rice, there are more than 13,291 alternatively spliced genes, which represent approximately 53.3% of the multiexon genes in the rice genome [54]. For bZIP TFs in rice, alternative splicing also exists. *OsABI5* was reported to have two transcripts, *OsABI5*-1 and *OsABI5*-2, and was the first bZIP TF reported as an AS gene in rice [52]. In this study, we obtained a novel gene *OsbZIP81* by homologous cloning (Figure 1). Through bioinformatics analysis, we found that *OsbZIP81* has three transcripts, one from NCBI (*OsbZIP81.1*) and two from RGAP (*OsbZIP81.2* and *OsbZIP81.3*). Of the three deduced proteins, OsbZIP81.1 and OsbZIP81.2 have the same N-termini (from 1–210 aa) but different C-termini, whereas OsbZIP81.3 is part of OsbZIP81.1 (from 127–326 aa) (Figure 2). We successfully cloned these three transcripts using RT-PCR with gene specific primers. However, we only confirmed the existence of *OsbZIP81.1* and *OsbZIP81.2*. To confirm the existence of *OsbZIP81.3*, further validation with experiments, such as RACE or full-length cDNA sequencing, is needed. A research group has reported the acquisition of 11,733 validated splicing isoforms by full-length cDNA sequencing (PacBio) [56]. Although we did not find alternative splicing information for *OsbZIP81* in that paper, we believe that this problem will be resolved with the publication of much more data in the future.

### 3.2. OsbZIP81.1 and OsbZIP84 are Typical bZIP Transcription Factors

In plants, the bZIP TF family is one of the largest transcription factor families. It is involved in almost all biological processes and it plays a vital role in response to environmental stresses [35,57]. A typical bZIP transcription factor possesses two conserved regions: the basic region and the leucine zipper region. The former contains ~16 amino acid residues of nuclear localization signal, followed by a conserved N-X7-R/K sequence, which can directly bind to a specific DNA sequence. The latter, which was located at the C-terminus of the former conserved region, has a typical structure in which the seventh of every seven amino acids contains one leucine or another hydrophobic amino acid and the first leucine is nine amino acid residues away from the conserved R/K amino acid (Figure 2) [12,13]. In this study, all three transcripts of OsbZIP81 and OsbZIP84 that we cloned had a typical bZIP structure (Figure 1). However, only OsbZIP81.1 and OsbZIP84 have strong self-activation and transactivation activities in yeast and rice protoplasts. Both OsbZIP81.2 and OsbZIP81.3 only had weak self-activation and transactivation activities (Figure 3). We also obtained little sequence data from ChIP-Seq with OsbZIP81.2ox (Supplementary File S4). In addition, OsbZIP81.1 and OsbZIP84 could form homo- and heterodimers with themselves and each other (Figure 4A,B). Moreover, OsbZIP81.1 and OsbZIP84 were both localized in the nucleus and cytoplasm (Figure 3). Taken together, these results indicate that OsbZIP81.1 and OsbZIP84 are typical bZIP transcription factors with strong transactivation activities, while OsbZIP81.2 and OsbZIP81.3 may not be.

### 3.3. OVRE is a Novel Motif for Group IX bZIPs in Rice

Information regarding TF-bound *cis*-acting elements can provide insight into transcriptional regulation and reveal, in depth, the functions of TFs. Most bZIP TFs can recognize similar *cis*-acting elements, such as those with the core sequence of ACGT, including CACGTG (G box), GACGTC (C box), and TACGTA (A box), due to their similar DNA binding domains. The promoter region of most genes induced by light or abscisic acid, auxin, jasmonic acid, and salicylic acid contain these elements [14]. In rice, most TFs that can recognize these *cis*-elements belong to group I, IV, VI, and IX of bZIPs [14]. For group IX, CCA(N)_n_TGG has been reported to bind the motif of RF2a and RF2b [37,38,39]. Moreover, the binding motifs of bZIP TFs belonging to the same subfamily of RF2a and RF2b have been identified in other species, such as VRE (core sequence: ACNGCT, Arabidopsis VIP1) [31], GCTCCGTTG (tomato VSF-1) [58], TCCAGCTTGA, and TCCAACTTGGA (tobacco RSG) [45]. However, the dissimilarity among RF2a/RF2b, VIP1, VSF-1, and RSG binding motifs suggest that, despite high homology in the bZIP domains (Figure 1A), the DNA binding preferences are not always conserved. In this study, we identified 15 motifs from the ChIP-Seq data analysis (Supplementary Table S1). Of the 15 motifs, the most enriched motif OVRE (core sequence: GCTG) was close to VRE, but it was not completely the same. In addition, we performed an RDSA assay with OsbZIP84, which can form heterodimers with OsbZIP81 (Figure 4B), and it obtained three motifs (Supplementary Table S2). The most significantly enriched motif was basically consistent with the OVRE motif that we obtained from ChIP-Seq of OsbZIP81.1 (Figure 5C,E). These results suggest that the binding motifs are likely conserved if these TFs are sufficiently close. Moreover, no similar binding motifs of rice bZIPs have been reported to our knowledge. Thus, OVRE is a novel binding motif of rice bZIPs, that provides new insights for the researcher to study bZIP TFs in eukaryotes.

### 3.4. OsbZIP81 May Positively Affect Endogenous JA Levels through Directly Binding and Regulating Genes in JA Signaling and Metabolism Pathway

Plants have evolved complex defense systems to protect themselves from herbivores and pathogens to survive under a changing environment. In these defense systems, plant hormones play an indispensable role [59,60,61]. JA, SA, and ET are such hormones related to defense [62,63,64]. JAs are lipid-derived compounds that act as key signals in plant stress responses and development [9,65]. Some bZIP members in plants are also involved in herbivore and pathogen resistance through JA signaling and the metabolism pathway [65,66,67]. JA and related compounds ubiquitously exist in land plants and function in plant development and responses to numerous stresses [5,9,68,69]. The triunsaturated fatty acid α-linolenic acid (18:3) (α-LeA) derived from chloroplastic glycerolipids is a substrate of JA biosynthesis. Another substrate is hexadecatrienoic acid (16:3) [9,70,71]. We found that exogenous MeJA could significantly (more than 10-fold) induce *OsbZIP81.1* and *OsbZIP81.2* (Figure 8B). In addition, by analyzing DEGs from the RNA-Seq data, we found that approximately one-third of the genes in the α-LeA metabolism pathway were enriched, 15 in OsbZIP81.1ox, and 11 in OsbZIP81.2ox. All 11 DEGs in OsbZIP81.2ox were included in the 15 DEGs in OsbZIP81.1ox (Supplementary Files S10 and S11). When combining RNA-Seq and ChIP-Seq data, we obtained seven genes that were enriched in JA signaling and the metabolism pathway (LOC_Os01g27230, LOC_Os01g27240, LOC_Os02g10120 (*OsLOX5*), LOC_Os03g32314 (*OsAOC*), LOC_Os05g07090, LOC_Os08g39840 (*OsHI-LOX*), LOC_Os12g26290 (*OsPIOX*)) that OsbZIP81.1 probably directly regulates.

LOXs, which catalyze the conversion of α-linoleic acid to hydroperoxy-octadecadienoic acid, are key enzymes in JA synthesis [9]. A research group recently reported that OsLOX2/5 may be hijacked by *M. oryzae* strain Guy11 to subvert host immunity and facilitate pathogenicity, which means that inducing the expression of OsLOX2/5 may improve resistance to the rice blast disease [72]. Moreover, OsHI-LOX is involved in herbivore-induced JA biosynthesis, and its increasing expression can enhance plant resistance to chewing herbivores in rice [73,74,75,76]. OsAOC (rice allene oxide cyclase), which catalyzes the conversion of 12,13-EOTrE to 12-OPDA, is a functional enzyme in the biosynthesis of JA and related compounds [77,78,79]. In another report, OsAOC was found to participate in the defense response against blast fungus that was mediated by the regulation of JA synthesis [78]. OsPIOX is a fatty acid α-oxygenase gene, which plays an important role in the α-linolenic acid metabolism pathway [80]. In some plants, PIOX also be found as a pathogen-inducible oxygenase [81]. In addition, we observed that the leaves of the *osbzip81* mutant had many more disease scabs than the wild-type (Supplementary Figure S9). Taken together, all four genes, OsLOX5, OsHI-LOX, OsAOC, and OsPIOX, could provide a positive response for defense against pathogen infection by regulating JA synthesis. These results indicate that OsbZIP81.1 could enhance pathogen resistance by directly regulating the expression of the four genes. However, further experiments are needed to reach a conclusion.

### 3.5. OsbZIP81 MAY Play Roles in Agrobacterium-Mediated Transformation and Pathogen Resistance in Rice

*Agrobacterium*-mediated plant transformation is a very effective genetic research tool, and its development has accelerated the research process of a variety of organisms. In the process of transformation, at least five *Agrobacterium* virulence effector proteins (VirE2, VirE3, VirF, VirD2, and VirD5) were transferred into plant cells to facilitate the T-DNA transfer [42,82]. In the plant cells, VirE2 can bind to single strand T-DNA and protect it from being degraded [83,84]. In Arabidopsis, VirE2 can interact with VIP1, and this interaction complex plays a vital role in the *Agrobacterium*-mediated Arabidopsis transformation process [18,21,85]. After phosphorylation, VIP1 can shift from the cytoplasm to the nucleus and regulate the expression of the pathogenesis-related gene *PR1* [31]. Simultaneously, *Agrobacterium* can abuse the MAPK-targeted VIP1 defense signaling pathway for nuclear delivery of the T-DNA complex as a Trojan horse [20,31]. In rice, *Agrobacterium*-mediated transformation has been regularly used for genetic improvement [86,87,88]. However, *Agrobacterium*-mediated transformation of many *indica* varieties still faces difficulties, especially with large fragments [43,89]. In this study, we found that a rice protein OsbZIP81.2 could interact with the *Agrobacterium* virulence protein VirE2 (Figure 7A, Supplementary Figure S2). Furthermore, we found that VirE2 can interact with the truncated OsbZIP81.1 containing the bZIP domain but not the C-terminus (Figure 7A). This phenomenon could be due to the altered protein structure that is caused by the C-terminus of OsbZIP81.1. By IP-MS with OsbZIP81.2-flag overexpression transgenic rice plants and anti-flag antibody, we also identified a 14-3-3 protein, and this result was verified by Y2H (data not shown). Based on these studies, we believe that OsbZIP81.2 has a potential role in *Agrobacterium*-mediated rice transformation. Our findings may provide an opportunity to improve the transformation efficiency.

PR proteins have been defined as a kind of plant protein that is induced not only during pathogen infection, but also in response to abiotic stress, including wounding, drought, and high salinity [90]. Most PRs and related proteins are induced through the action of the signaling compounds SA, JA, or ET, and they possess antimicrobial activities in vitro through hydrolytic activities on cell walls and contact toxicity [91]. In Arabidopsis, VIP1 can regulate *PR1* expression in an indirect manner during stress responses [31]. In rice, no bZIP-interacting PR proteins have been reported to our knowledge. In this study, we found two PR proteins, OsPR10a and RSOsPR10, which could interact with the rice VIP1 homologue OsbZIP81.2 (Supplementary Figure S8). We did not find strong binding site(s) at the promoters of OsPR10a and RSOsPR10 based on the ChIP-Seq data of OsbZIP81.1. However, when combining the RNA-Seq and ChIP-Seq data, we found a putative PR gene, LOC_Os01g14590, containing a strong binding site at its promoter region (Supplementary Figure S7E), and some genes related to plant disease response (Supplementary Table S5). Further analysis of the function of these genes and their relationship with OsbZIP81 may provide new insight in understanding whether the bZIP transcription factor OsbZIP81.1 can directly regulate these genes to enhance plant disease resistance.

We obtained a T-DNA insertion mutant of *OsbZIP81* (PFG_3A-08084, renamed as *osbzip81*) from SIGnAL (http://signal.salk.edu/). Unfortunately, we only achieved heterozygote genotype plants. The homozygote *osbzip81* plant grew to a much lower height and had many more scabs on the leaves than wild-type Dongjin, and it was unfruitful (Supplementary Figure S9). Furthermore, we found that the levels of endogenous JA and SA were up-regulated in OsbZIP81.1ox plants (Figure 10D).

**Figure 11:**
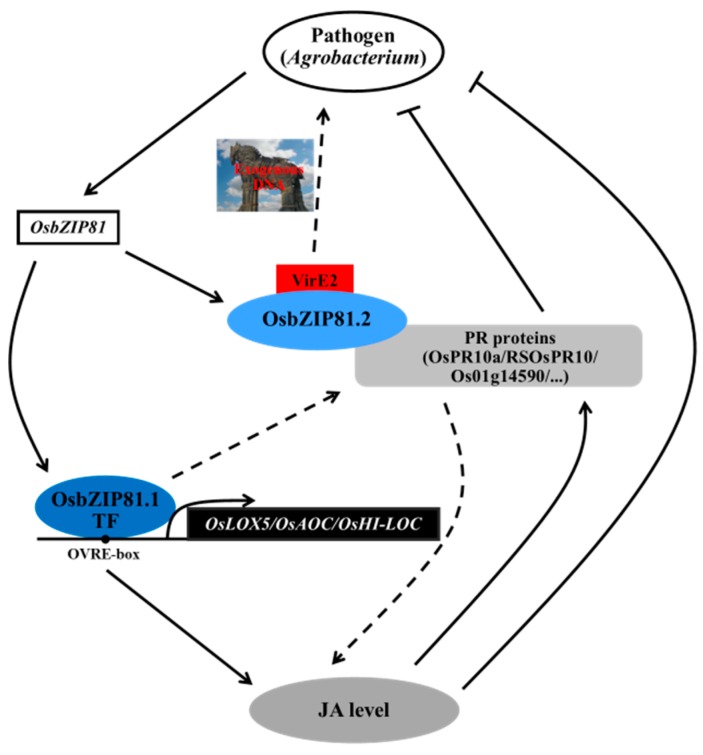
A working model for OsbZIP81. A proposed model for the OsbZIP81-mediated pathogen response by regulating PR proteins and genes in the JA metabolism and signaling pathway in rice. The image ‘Trojan horse’ was downloaded from https://image.baidu.com.

Collectively, to understand the mechanism of *Agrobacterium*-mediated rice transformation, Arabidopsis *VIP1* was used to find homologues in rice. Finally, we identified two homologues of Arabidopsis *VIP1*: *OsbZIP81* and *OsbZIP84*. Further study showed that *OsbZIP81* is an AS gene, and its two (or three) transcripts have different structures, as well as different transactivation activities and interaction partners, which may imply different roles in rice development and responses to multiple environmental stresses. We also found that OsbZIP81 might directly regulate PR proteins and the enzymes in JA synthesis to positively affect endogenous JA and SA, which may enhance the resistance to pathogens and other diseases (Figure 11).

## 4. Materials and Methods

### 4.1. Plant Materials and Growth Conditions

Rice Zhonghua 11 (ZH11; *Oryza sativa* ssp. japonica) was used as the wildtype. The OsbZIP81.1-overexpression line and the *osbzip81* mutant were constructed in this study. Seedlings were grown under natural long-day conditions (approximately 14 h light/10 h dark) from June to September at Wuhan. Two-week-old seedlings were used for the abiotic and biotic treatments. For all of the samples, the shoots of the seedlings were harvested and frozen in liquid nitrogen for RNA isolation or immediately placed in 1% formaldehyde for chromatin isolation.

To generate the overexpression OsbZIP81.1 transgenic rice line, the genome fragment containing the full-length OsbZIP81.1 cDNA fragment amplified with the specific primers OsbZIP81.1-F/R and cloned into the binary expression vector pCAMBIA1301U-flag (driven by a maize ubiquitin promoter and fused to 3× flag tags at its C terminus) at the *Kpn*I and *Bam*HI sites. The constructed vector was introduced into rice ZH11 by *Agrobacterium*-mediated transformation [92].

### 4.2. Subcellular Localization Assay in Rice Protoplasts

The vector pM999-GFP was used to study the subcellular localization of OsbZIP81.1, OsbZIP81.2, and OsbZIP84. The full-length cDNA fragment was amplified from pGADT7-OsbZIP81.1 using the following specific primer pair: GFP-OsbZIP81.1-F/R (Supplementary Table S6) and cloned into the pM999-GFP vector. The pM999-OsbZIP81.2-GFP and pM999-OsbZIP84-GFP were constructed in the same way.

For rice protoplast preparation and transformation, 14-day-old yellow seedlings of ZH11 (*O. sativa* ssp. japonica) after germination in half-strength MS medium were used in this study. The protoplasts were isolated according to previous reports with little modifications [93,94]. In general, the rice protoplasts were isolated by digesting the rice sheath strips in digestion solution (0.6 mol/L mannitol; 10 mmol/L MES, pH 5.7; 1.5% cellulose R-10; 0.75% macerozyme R-10; 0.1% BSA; 1 mmol/L CaCl_2_) for 4 to 5 h at 28 °C at a speed of 50 rpm in a dark table concentrator. The protoplasts were then incubated in W5 solution (154 mmol/L NaCl; 125 mmol/L CaCl_2_; 5 mmol/L KCl; 2 mmol/L MES, pH 5.7) at the 28 °C and 80 rpm for 10 min. The protoplasts were collected by centrifugation at 100× *g* and 4 °C for 8 min. after filtering through 300-mesh filter (50 μm). The supernatant was removed and the pellet resuspended in another 4 mL W5 solution. The protoplasts were collected after another centrifugation at 100× *g* and 4 °C for 8 min. and resuspended in MMG solution (0.6 mol/L mannitol; 15 mmol/L MgCl_2_; 4 mmol/L MES, pH 5.7) to a final concentration of 1.0 × 10^7^ mL^−1^. For transformation, 5 μL of each plasmid (5–10 μg) was pooled and gently mixed with 100 μL of protoplasts and 110 μL of PEG-CaCl_2_ solution (40% PEG33500, 0.6 mol/L mannitol, 100 mmol/L CaCl_2_), and then incubated at 28 °C for 15 min. in the dark. Transformation was stopped by the addition of two volumes of W5 solution. The transformed protoplasts were then collected by centrifugation and then re-suspended in WI solution (0.6 mol/L mannitol; 4 mmol/L KCl; 4 mmol/L MES, pH 5.7). The transformed protoplasts were maintained in 12-well culture plates at 28 °C for 12–16 h in the dark. After incubation, the transformed protoplasts were collected by centrifugation at 100× *g* for 8 min. and observed by fluorescence confocal microscopy (Leica Microsystems SP8, Wetzlar, Gemany).

### 4.3. Dual Luciferase Transcriptional Activity Assay in Rice Protoplasts

The full-length CDS of *OsbZIP81.1* and *OsbZIP81.2* was cloned into GAL4-DB via the *Bam*HI/*Eco*RI sites as the effector (the primer sequences are listed in Supplementary Table S6), and the 35S promoter driven luciferase gene (35S-GAL4-fLUC) and basic promoter driven fluorescent luciferase gene (GAL4-fLUC) were used as reporter to detect the transcriptional activation of OsbZIP81.1 and OsbZIP81.2. The promoter of OsPIOX was cloned into 190LUC via the *Hind* III/*Bam*HI. The internal reference vector was the luciferase gene (rLUC). Effectors, reporter, and internal reference plasmids were extracted while using the Qiagen plasmid Midi Kit, and the final concentration of these plasmids was approximately 1 μg/μL. Subsequently, 3 μg effectors, 3 μg reporters, and 0.5 μg internal reference plasmids were transformed into rice protoplasts by PEG mediated transformation. The transformation methods were performed, as described above (Section 4.2). The transformed protoplasts were cultured in the dark for 12 h or overnight and then collected and detected while using the Dual-Luciferase Reporter Assay System kit (E1910, Promega (Beijing), Beijing, China). Supplementary Table S6 lists the primers used for these different genes.

### 4.4. ChIP Sequencing

Overexpressed OsbZIP81.1 rice under normal condition was used for ChIP-Seq analysis. ChIP was performed, as described previously, with some modifications [94]. Briefly, leaf tissues from four-leaf-stage seedlings were immediately fixed after harvest in 1% formaldehyde under vacuum for 30 min and 1.5 g tissues were used for chromatin isolation. Isolated chromatin was sheared to approximately 200 bp using a supersonic instrument (Bioruptor Plus, DIAGENODE, Belgium), as follows: high power, cycle conditions 30/90 (On/Off times in s), 30 cycles. For ChIP-Seq, the DNA was immunoprecipitated by anti-flag antibody, as described previously, and the precipitated DNA was purified and solubilized in distilled water. For each library, three independent replicated samples were mixed together to generate the sequencing library, which was processed by Wuhan Igenebook Company.

ChIP-Seq data processing and analysis were performed as described by Zong [95]. Briefly, raw sequencing reads from each library were mapped to the rice genome (RGAP ver. 7.0, http://rice.plantbiology.msu.edu/) while using SOAP2 [96], and only uniquely mapped reads were used for peak identification. The Model-based Analysis of ChIP-Seq (MACS) software was used to identify OsbZIP81.1-associated regions with default parameters [97]. The .wig files of the MACS output were visualized using the Integrated Genomics Viewer [98]. A gene was regarded as an OsbZIP81.1-bound gene if the promoter region of the gene (including 2 kb upstream of the transcription start site) had at least 1 bp overlapping with the peaks. We extracted 200 bp around the peak summits (100 bp upstream and 100 bp downstream) of each library and the 1000 highest *q*-value peaks were subjected to MEME-ChIP (http://meme-suite.org/tools/meme-chip) to identify the enriched motifs [49,99]. For functional category analysis, KEGG pathway information was collected from the KEGG database [100], and the functional category (Rice_japonica_mapping_merged_08 download) was collected from the Mapman web site [101].

### 4.5. ChIP-qPCR

The ChIP product was analyzed by quantitative real-time PCR (the primer sequences are listed in Supplementary Table S6) with a CFX96 Real-Time System (Bio-Rad). Three replicates of each sample were evaluated and the enrichment values were normalized to the input sample and *Actin* was used as the reference gene. Supplementary Table S6 lists the primers used here.

### 4.6. Random DNA Binding Selection Assay (RDSA)

RDSA was used to identify the motif(s) for OsbZIP84 binding. Purified GST-tagged OsbZIP81 protein was used in this experiment, and the experimental protocol was performed according to Dr. Wang [38]. The primers used can be found in Supplementary Table S6. After seven cycles, purified DNA was ligated into the pGEM-T easy vector (Promega, Cat. # A3600) and then transformed into the DH10B strain. The plasmids from monoclones were extracted and sequenced. Supplementary Table S6 lists the primers.

### 4.7. Electrophoretic Mobility Shift Assay

GST-fused OsbZIP81.1 and GST tag proteins were expressed and purified, as described above. Oligonucleotides were synthesized and labeled with a biotin-tag at their 5′ end by TSINGKE Biological Technology Company (Supplementary Table S6). To generate the double-stranded oligos, an equal amount of the complementary single-stranded oligos was mixed and run using the following program: 95 °C for 1 min., 55 °C for 1 min., 72 °C 5 min., two cycles, and annealed by gradually cooling down to 4 °C. The LightShift Chemiluminescent EMSA Kit was used for the EMSA experiment (20148, Thermo Scientific) following the manufacturer’s instructions. The competition assay was performed, as follows. Unlabelled DNA was incubated with protein and other materials at room temperature (~25 °C). After 20 min., 2 μL biotin-labeled DNA was added and incubated at room temperature for 20 min. The reactions were then subjected to electrophoresis on 6% polyacrylamide gels running with 0.5× TBE buffer at 4 °C until the bromophenol blue dye had migrated approximately 2/3 to 3/4 down the length of the gel. The next steps were performed according to the instructions provided with the kit. Finally, the signals were detected with X-ray films (ChemiScope 5000Pro, CLiNX, Shanghai, China).

### 4.8. Yeast Two-Hybrid and Library Screening Assay

Full-length cDNA was amplified using specific primers. The obtained fragments were cloned into the pGBKT7 or pGADT7 vector (Clontech, Mountain View, CA, USA), depending on the different restriction sites. Supplementary Table S6 lists the primers and restriction sites that were used for these different genes. These plasmid pairs were used to cotransform the yeast strain AH109 according to the manufacturer’s instructions (Clontech). The transformed yeast cells were grown on SD medium lacking Leu and Trp (SD/-Leu-Trp) and then transferred to SD medium lacking Leu, Trp, Ade, and His, and were supplemented with 40 μg·mL^−1^ X-α-Gal (SD/-Leu-Trp-Ade-His + X-α-Gal).

For yeast two-hybrid screening, a library was constructed with rice seedling cDNA and kept in our laboratory was used (Y187 strain). The screening was performed using pGBKT7-OsbZIP81.2 (AH109 strain) with 5 mmol/L 3-Amino-1,2,4-triazole (3-AT). The mating procedures followed the Yeastmaker^TM^ Yeast Transformation System 2 User Manual (Clontech, Takara (Beijing), Beijing, China).

### 4.9. BiFC Assay

The OsbZIP81.2 was cloned into the pSPYCE(M) vector and VirE2 was cloned into the Pspyne173 vector [102]. Supplementary Table S6 lists the primers used for the vector construction. The two vectors were mixed and transformed into the rice protoplasts, as described above. After incubation in the dark for 16 h, the fluorescence was observed by confocal microscopy. The primers used can be found in Supplementary Table S6.

### 4.10. Glutathione S-Transferase (GST) Pull-down Assay

For the GST pull-down assay, the full-length coding sequences of *OsbZIP81.2* and *VirE2* were cloned into pTXB3 and pGEX-6p-1 vectors, yielding CBD-OsbZIP81.2 and GST-VirE2, respectively. The constructed vectors were transferred into *Escherichia coli* BL21 (DE3) cells for the expression of fusion proteins. Two purified proteins were mixed with equal volumes and incubated in 1 mL PBS buffer for 6 h at 4 °C. One-hundred microliters of Glutathione Sepharose 4B beads (GE Healthcare) were added into the protein mixture and incubated for another 2 h at 4 °C. The beads were washed five times with PBS buffer and the pulled proteins were eluted by boiling and further analyzed by immunoblotting using anti-GST (ABclonal) and anti-CBD (NEB). The primers that were used can be found in Supplementary Table S6.

### 4.11. Multiple Stress Treatment

To detect the sensitivity of OsbZIP81 and other members of the same bZIP subfamily under multiple treatments, ZH11 rice plants were grown in the greenhouse with a 14-h-light/10-h-dark cycle. Two-week-old seedlings were treated with chemical or abiotic stress. Chemical treatments were conducted by spraying leaves with 0.1 mmol/L ABA, 0.1 mmol/L MeJA, 0.1 mmol/L SA, 10 μmol/L C_2_H_4_, 10 μmol/L NAA, and 10 μmol/L IAA, followed by sampling at 0, 3, 6, 12, and 24 h, or irrigating the plants with 20% PEG6000, followed by sampling at 0, 1, 5, 12, and 14 h. For cold and heat stress, the seedlings were transferred to a growth chamber at 4 or 42 °C and sampled at 0, 1, 3, 6, 12, and 24 h after treatment. Incubating two-week-old seedlings with 200 mmol/L NaCl solution, followed by sampling at 0, 1, 3, 6, 12, and 24 h after treatment performed salt stress. Two-week-old seedlings were placed in air without a water supply and sampled at 0, 1, 3, 6, 12, and 24 h. AS (100 μmol/L) alone or with *Agrobacterium* (EHA105 strain) were performed while using the rice callus and followed by sampling at 0, 1, 3, 6, 12, 24, 48, and 72 h after treatment. Every treatment was performed at least three times.

### 4.12. RNA-Seq and Data Analysis

RNA-Seq was used to identify the target genes of OsbZIP81.1 by integrating the analysis with the ChIP-Seq data. Total RNAs were extracted from four-leaf-stage rice seedlings using RNAiso Plus (Takara) reagent according to the user manual. For library construction, three independent replicated RNA samples were prepared and each 10 μg of total RNA was used for RNA-Seq by Novogene Company (Beijing, China). The libraries were then sequenced with an Illumina HiSeq 3000. The *O. sativa* genome (RGAP v. 7.0) was used as a reference. The gene expression levels were calculated by using the reads per kilo bases per million reads (RPKM) method. To identify the differentially expressed genes between the libraries, edgeR software was applied to identify DEGs. The fold change (|log2FC| ≥ 1) and *p*-value (*p* ≤ 0.05) were used as the indexes of statistical significance.

### 4.13. Real-Time qPCR

The total RNAs were isolated from rice seedlings using RNAiso Plus (Takara) reagent according to the manufacturer’s instructions. RNAs (2 μg) were used for cDNA synthesis with the PrimeScript RT reagent Kit with gDNA Eraser (Takara). SYBR Green Realtime PCR Master Mix (TOYOBO, Shanghai) was used for real-time PCR analysis with the CFX96 Real-Time System (Bio-Rad). Three technical replicates were evaluated for each sample and *Actin* was used as the reference gene. The RT-qPCR profiles included the following steps: 94 °C for 3 min., followed by 45 cycles at 94 °C for 15 s, 60 °C for 15 s, and 72 °C for 15 s. Supplementary Table S6 lists the primer sequences.

### 4.14. Quantification of Endogenous JA, MeJA, SA and ABA

Half gram of four-leaf-stage rice samples were used for measuring the contents of endogenous JA, MeJA, SA, and ABA. Wild type rice ZH11 was selected as the control and three OsbZIP81.1 overexpression transgenic rice plants were sampled and mixed as the experiment group. Every group contains at least three samples (ZH11-1, ZH11-2, ZH11-3; OX-OsbZIP81.1-1, OX-OsbZIP81.1-2, OsbZIP81.1-3). Harvested samples were sent to company (ProNetsBio, Wuhan, China) to measure the hormones by HPLC-MS/MS.

### 4.15. Accession Numbers and Data Availability

The sequence data from this article can be found in the RGAP data base (http://rice.plantbiology.msu.edu/), under the following accession numbers: *OsbZIP81*, LOC_Os11g06170; *OsbZIP84*, LOC_Os12g06520; *RSOsPR10*, LOC_Os12g36830; *PBZ1*, LOC_Os12g36880; *OsMADS1*, LOC_Os11g34450; *Actin*, LOC_Os03g50855; *OsLOX5*, LOC_Os02g10120; *OsAOC*, LOC_Os03g32314; *OsHI-LOX*, LOC_Os08g39840; *OsPIOX*, and LOC_Os12g26290. The ChIP-Seq and RNA-Seq raw data are deposited in NCBI’S Sequence Read Archive (SRA) with accession code PRJNA510886.

## Figures and Tables

**Table 1:** Summary of ChIP-Seq data collected from OsbZIP81.1ox and OsbZIP81.2ox.

Sample	Total Reads	Mapped Reads	Paired	Single	SelfAND Mate	MapDiff CHR	Quality
OsbZIP81.1ox_IP	44705515	43192879 (96.62%)	21604458	373400	35769480	12784780	8179875
OsbZIP81.1ox_Input	35488065	34501154 (97.22%)	33180942	106591	34091530	775770	486020
OsbZIP81.2ox_IP	48091675	45489002 (94.59%)	43727660	146629	44858010	989478	622885
OsbZIP81.2ox_Input	39256499	38410324 (97.84%)	36286088	155149	37874736	1401852	936240

**Table 2:** List of genes and their putative function in the ChIP-Seq.

Gene ID	Nearest TSS	Putative Function (Reported Gene Name)	Fold Enrichment
LOC_Os05g08370	Chr5:4565177-4571461:-:-71	CESA1 - cellulose synthase	23.0974
LOC_Os04g56530	Chr4:33707427-33719236:-:-96	STE kinases include homologs to sterile 7, sterile 11 and sterile 20 from yeast	17.6621
LOC_Os08g32170	Chr8:19954661-19956231:-:-83	oxidoreductase, 2OG-FeII oxygenase domain containing protein	16.4529
LOC_Os04g54240	Chr4:32301622-32302456:-:-204	wound induced protein	16.4453
LOC_Os02g26160	Chr2:15363265-15367217:+:179	receptor lectin kinase like protein	16.1431
LOC_Os03g62700	Chr3:35480584-35486347:-:-161	protein kinase	15.8304
LOC_Os06g12660	Chr6:6915783-6916582:+:460	NHL repeat-containing protein	15.5848
LOC_Os05g50910	Chr5:29213556-29220234:+:154	extra-large G-protein-related	14.9281
LOC_Os08g35110	Chr8:22136791-22137879:+:221	OsSAUR33 - Auxin-responsive SAUR gene family member	14.2462
LOC_Os09g35010	Chr9:20395224-20396205:-:-147	dehydration-responsive element-binding protein (OsDREB1B)	13.8964
LOC_Os07g02200	Chr7:699844-700831:-:-70	plastocyanin-like domain containing protein	13.7476
LOC_Os10g39440	Chr10:21048271-21052794:+:167	transporter family protein (OsTMT1)	13.4741
LOC_Os09g07920	Chr9:4042693-4045478:+:249	NOI protein, nitrate-induced	13.3778
LOC_Os03g56820	Chr3:32375903-32378469:+:99	fatty acid hydroxylase (OsFAH2)	13.2101
LOC_Os03g22700	Chr3:13110912-13115736:-:-161	cyclin-dependent kinase C-2	13.2033
LOC_Os04g58250	Chr4:34680601-34685203:-:-421	protein kinase, putative	13.1943
LOC_Os10g33800	Chr10:17913818-17917850:+:215	lactate/malate dehydrogenase	13.0107

The genes listed in this table are limited to those associated with peaks that were enriched greater than 13-fold, located in the 500 bp upstream of the genes and have been classified with a known function.

## Supplementary Materials

Supplementary materials can be found at https://www.mdpi.com/1422-0067/20/9/2360/s1. Figure S1. Statistical analysis of the lengths of the OsbZIP81.1 bound regions. Figure S2. The interaction relationship between different type VirE2s and all members of the rice bZIP subfamily IX in yeast. Figure S3. Expression of *OsbZIP81* under different biotic and abiotic stresses. Figure S4. Expression profiles of *OsbZIP81.1* and *OsbZIP81.2*. Figure S5. Expression identification of OsbZIP81.1ox and OsbZIP81.2ox. Figure S6. Validation of differential expressed genes in RNA-Seq by qRT-PCR. Figure S7. Validation of the OsbZIP81.1 binding sites of partial genes by ChIP-qPCR. Figure S8. The interaction relationships between OsbZIP81.1 (or OsbZIP81.2) and the deduced proteins of the Y2H screened genes. Figure S9. The phenotype of *osbzip81* mutant. Supplementary File S1. List of enriched peaks and their location in the *O.stiva* genome (OsbZIP81.1ox). Supplementary File S2. List of enriched peaks and their location in the *O.stiva* genome. Supplementary File S3. List of potential genes bound by OsbZIP81.1. Supplementary File S4. List of enriched peaks and their location in the *O.stiva* genome (OsbZIP81.2ox). Supplementary File S5. List of DEGs in OsbZIP81.1ox_vs_WT. Supplementary File S6. List of DEGs in OsbZIP81.2ox_vs_WT. Supplementary File S7. List of genes bound and regulated by OsbZIP81.1. Supplementary File S8. Statistics of TFs in OsbZIP81.1 target genes. Supplementary File S9. List of genes in each of the enriched gene ontology (GO) categories. Supplementary File S10. List of the most enriched pathway term (*p*-value < 0.05; OsbZIP81.1ox_vs_ZH11). Supplementary File S11. List of the most enriched pathway terms (*p*-value < 0.05; OsbZIP81.2ox_vs_ZH11). Supplementary Table S1. Identified motifs in ChIP-Seq experiment with MEME online software. Supplementary Table S2. Identified motifs in RDSA experiment with DREME online software. Supplementary Table S3. Summary of RNA-Seq data collected from the rice shoots of the ZH11, OX-OsbZIP81.1, OX-OsbZIP81.2 and *osbzip81* under normal condition. Supplementary Table S4. List of clones screened by yeast two-hybrid assay. Supplementary Table S5. Partial genes related to plant disease response. Supplementary Table S6. List of the primers used in the study.

## Abbreviations

OVRE	Oryza VIP1 Response Element
TF(s)	Transcription Factor(s)
ChIP-Seq	Chromatin Immunoprecipitation Sequencing
RNA-Seq	RNA Sequencing
bZIP	Basic region/leucine zipper
3-AT	3-Amino-1,2,4-triazole
Y2H	Yeast Two-Hybrid
SA	Salicylic Acid
ABA	Abscisic Acid
JA	Jasmonic Acid
MeJA	Methyl Jasmonic Acid
EMSA	Electrophoretic Mobility Shift Assay
TSS	Transcription Start Site
WT	Wild Type
GFP	Green Fluorescent Protein
CFP	Yellow Fluorescent Protein
ChIP-qPCR	ChIP quantitative PCR
DEGs	Differentially Expressed Genes
α-LeA	α-Linolenic Acid
12-OPDA	12-Oxophytodienoic Acid
PR	Pathogen-Related
RT-PCR	Reverse Transcription-Polymerase Chain Reaction
CDS	Coding Sequence
RDSA	Random DNA Binding Selection Assay
LOX	Lipoxygenase
AOC	Allene Oxide Cyclase
RT-qPCR	Real-time quantitative PCR
