# Supplementary material for: OsbZIP81, A Homologue of Arabidopsis VIP1, May Positively Regulate JA Levels by Directly Targetting the Genes in JA Signaling and Metabolism Pathway in Rice
Source: Int J Mol Sci. 2019 May 13;20(9):2360. doi: 10.3390/ijms20092360 (PMC6539606; doi:10.3390/ijms20092360)
Supplement: Supplementary file 1 [file ijms-20-02360-s001.zip › Supplementary Figures 1~9.docx]

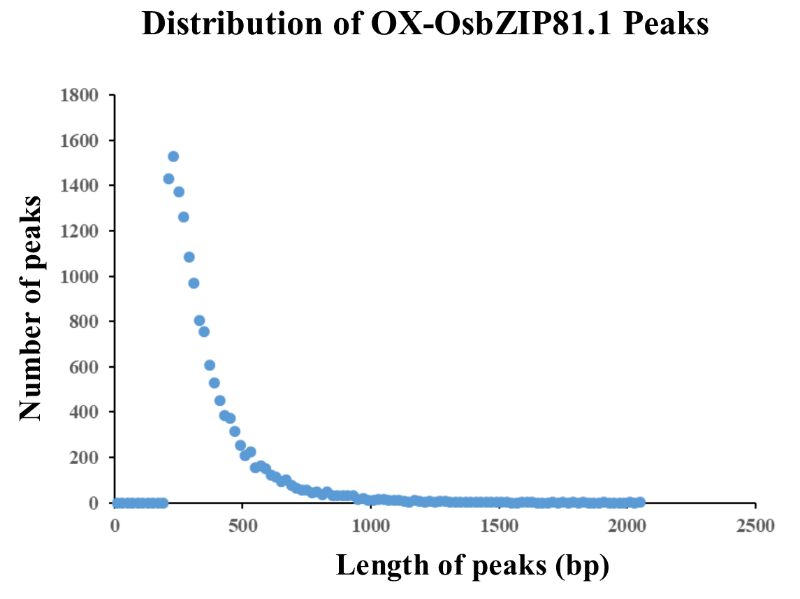
**Supplementary Figure 1. Statistical analysis of the lengths of the OsbZIP81.1 bound regions.**

**Supplementary Figure 2. The interaction relationship between different type VirE2s and all members of the rice bZIP subfamily IX in yeast.**


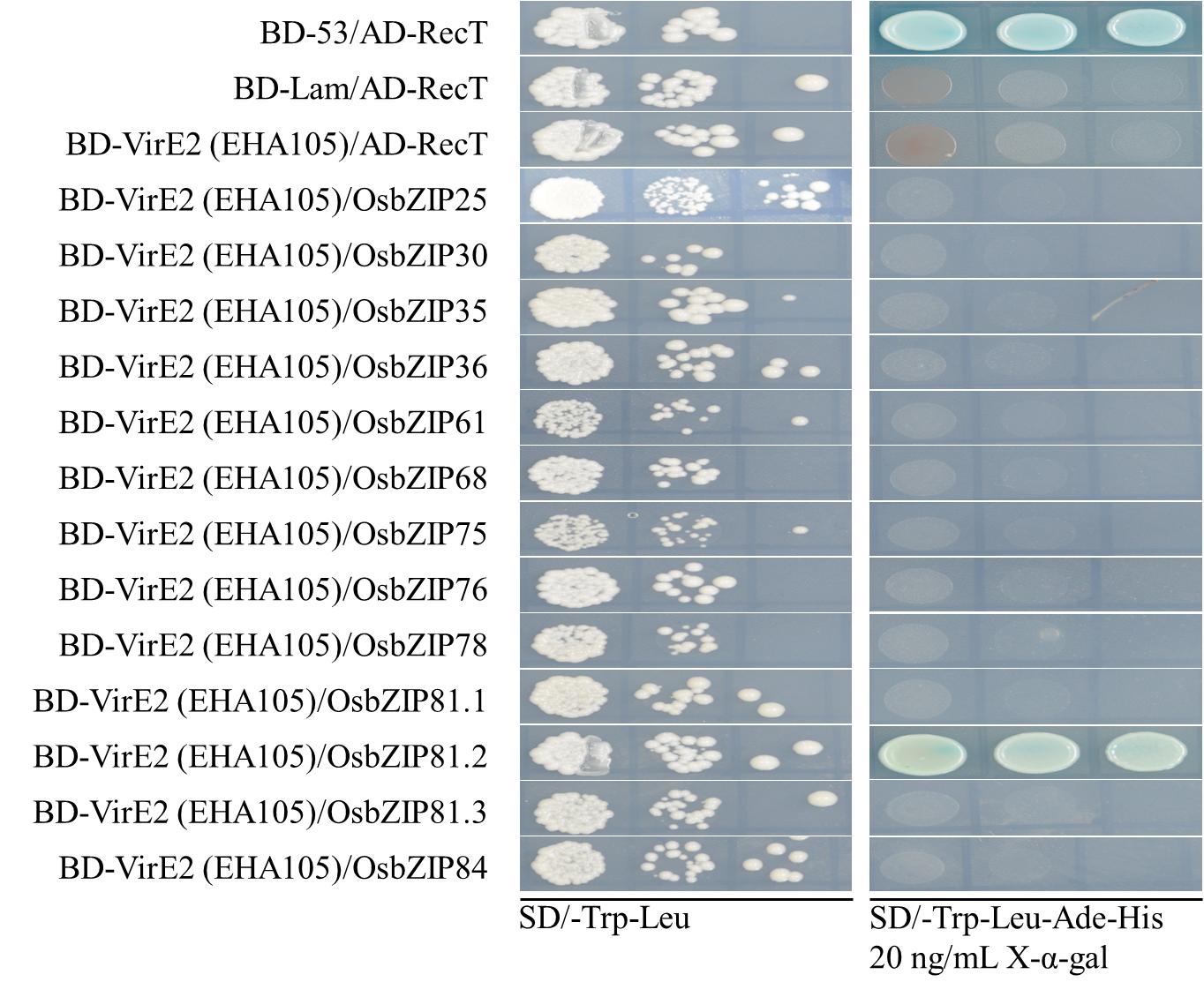


**A**


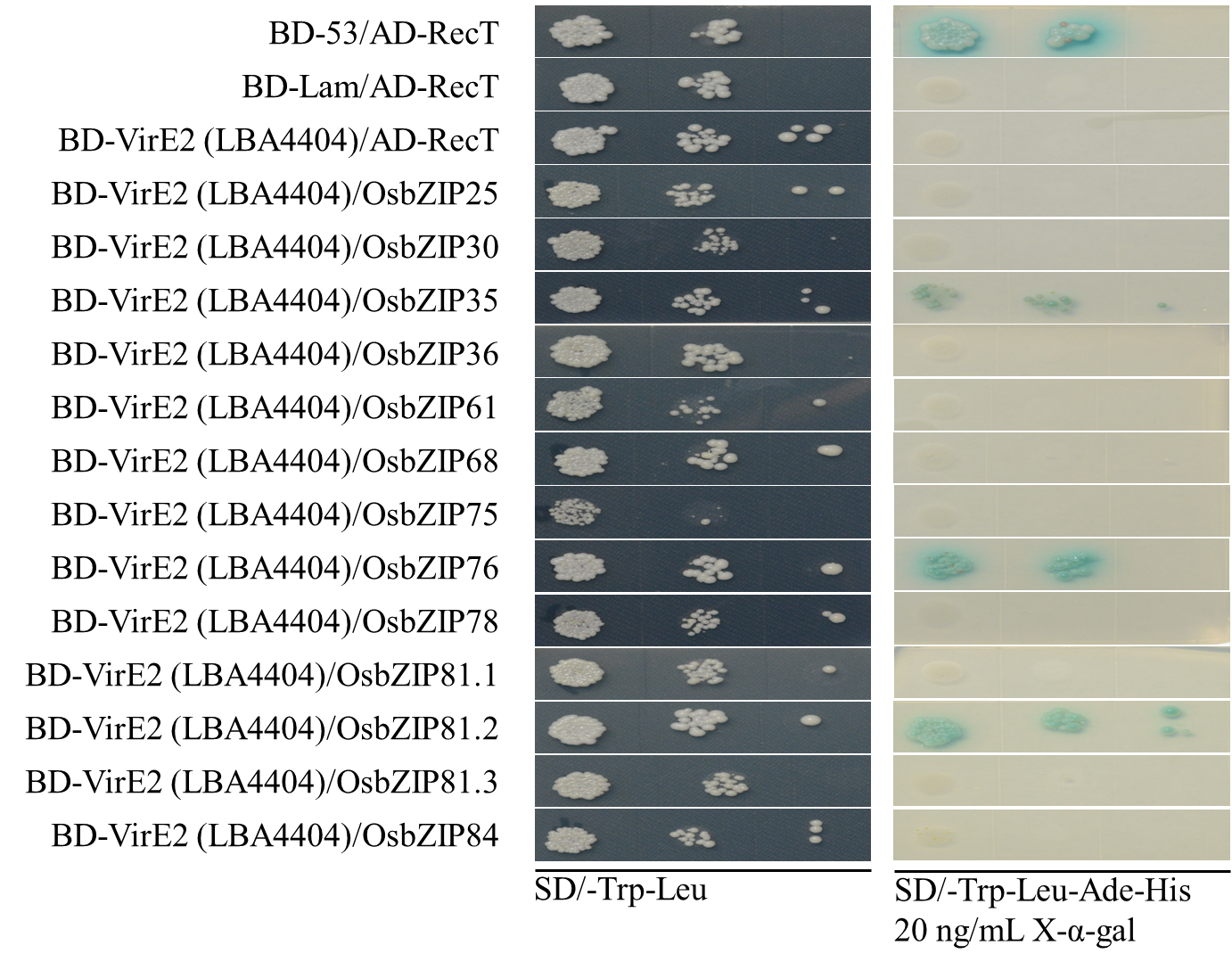


**C**


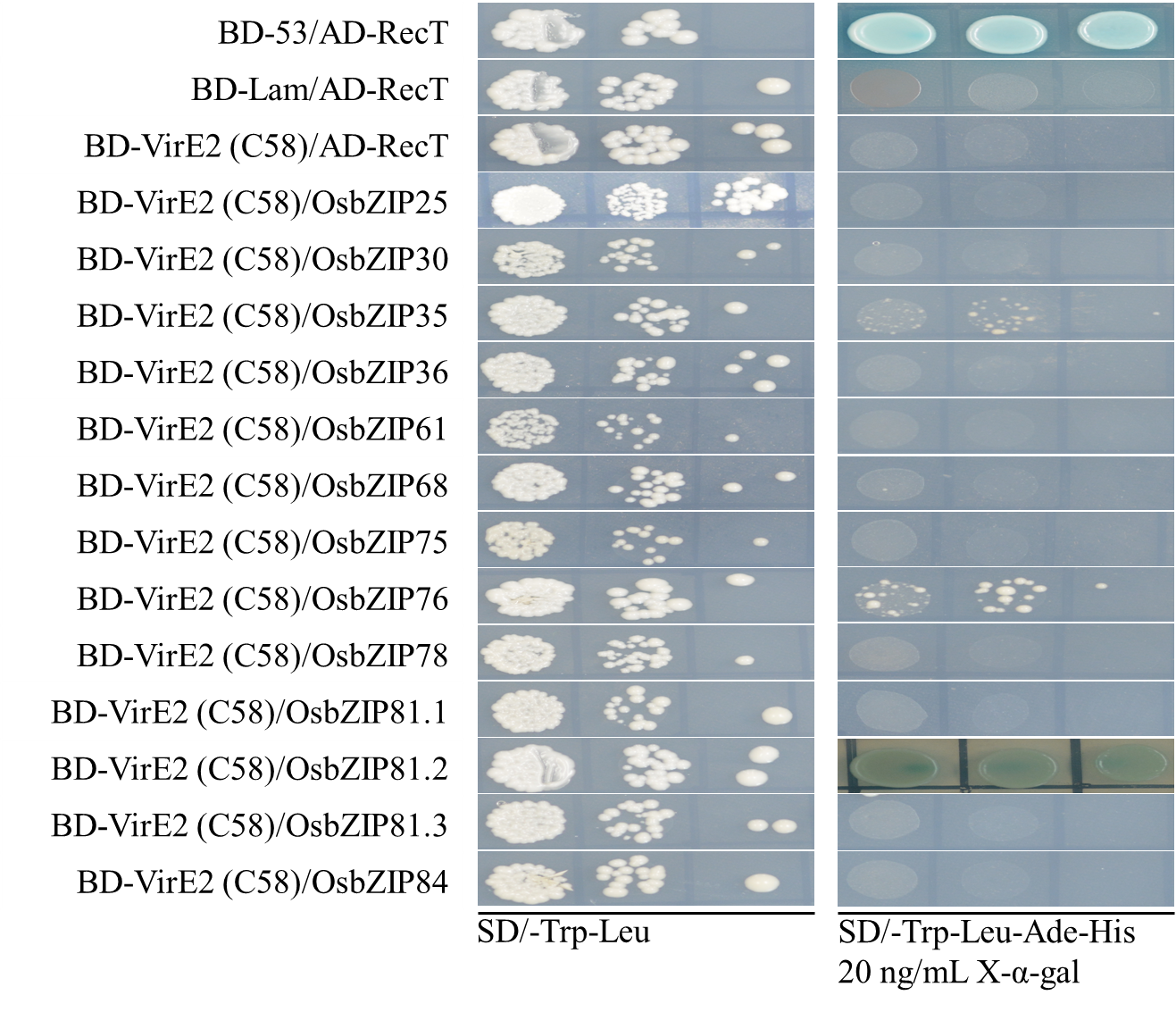


**B**

**(A)** The interaction relationship between agropine type VirE2 and all members of the bZIP subfamily IX in yeast. **(B)** The interaction relationship between nopaline type VirE2 and all members of the bZIP subfamily IX in yeast. **(C)** The interaction relationship between octopine type VirE2 and all members of the bZIP subfamily IX in yeast.

**Supplementary Figure 3. Expression of *OsbZIP81* under different biotic and abiotic stresses.**


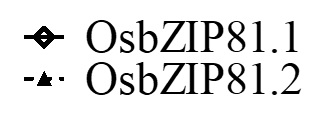


**A**


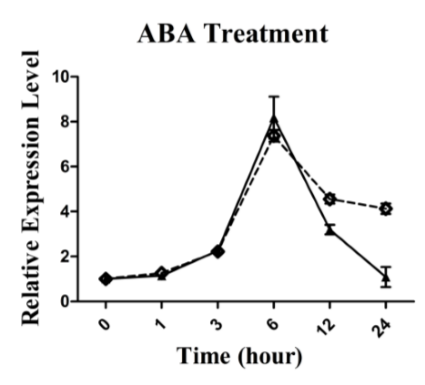


**B**


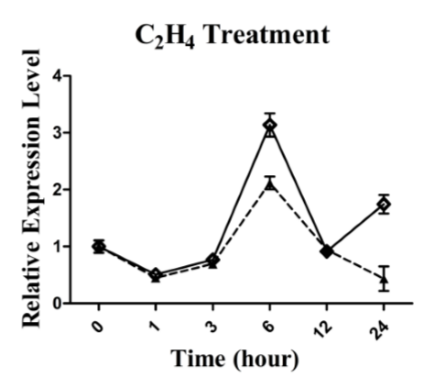


**C**


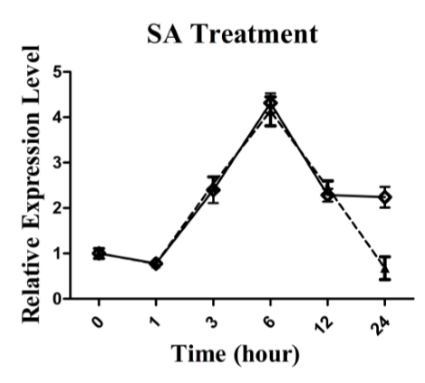


**D**


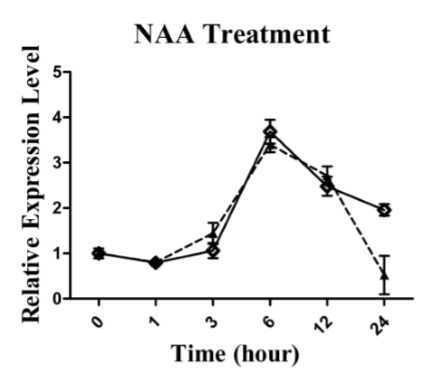


**E**


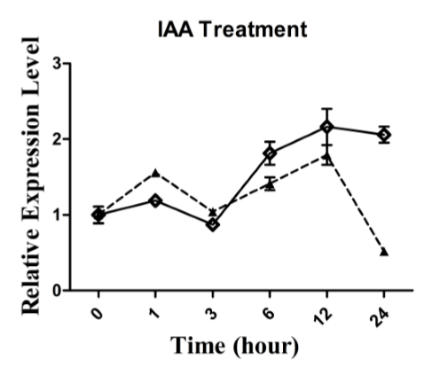


**F**


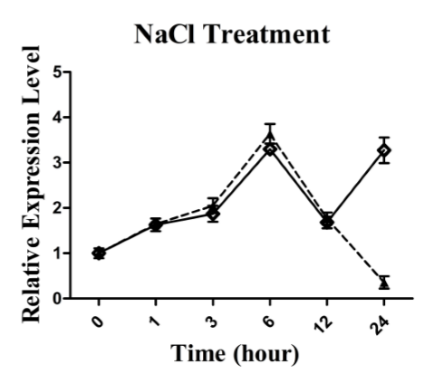


**G**


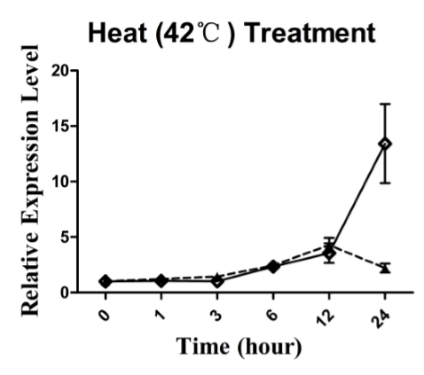


**H**


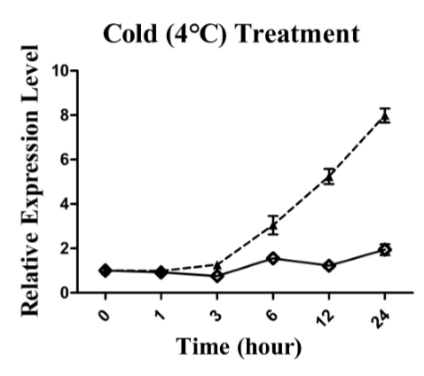


**I**

**(A)** Different transcripts presented in the following analyses. **Biotic stress: (B)**~**(F)** Hormone treatments (samples were harvested after 0, 1, 3, 6, 12 and 24 hours): ABA (100 μmol/L), C_2_H_4_ (10 μmol/L), SA (100 μmol/L), NAA (10 μmol/L), and IAA (10 μmol/L). **Abiotic stress:** **(G)~(I)** NaCl (200 mmol/L), Heat (42℃), and cold (4℃). All samples were harvested after 0, 1, 3, 6, 12 and 24 hours. The values in each column are the means of three independent replicates and error bars represent the SEM.


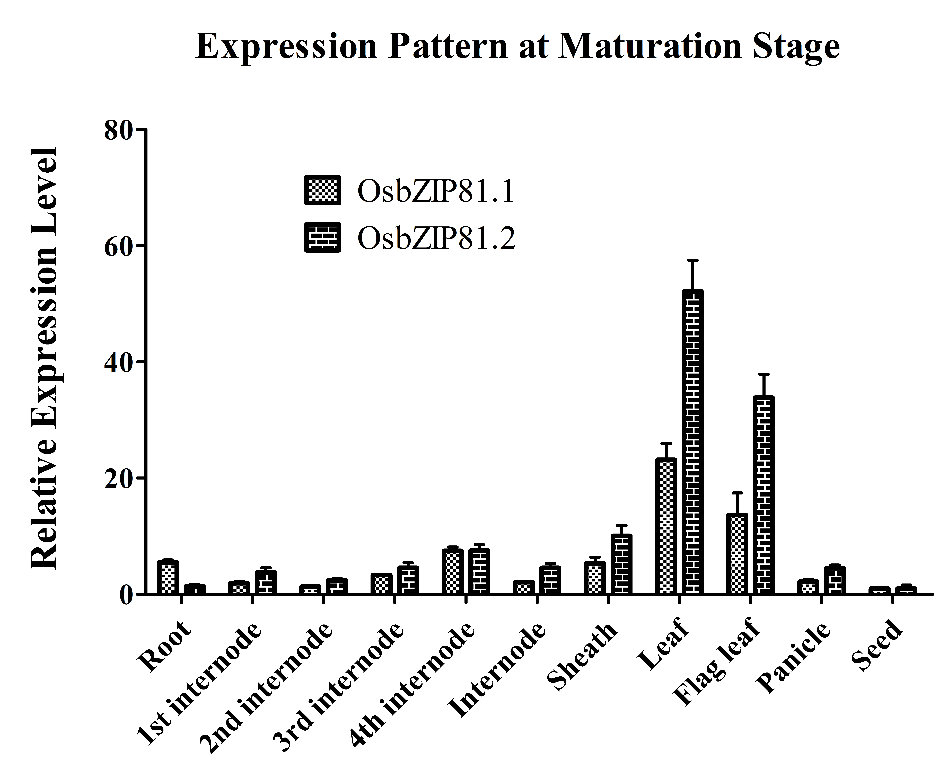
**Supplementary Figure 4. Expression profiles of *OsbZIP81.1* and *OsbZIP81.2*.**

Expression patterns of *OsbZIP81.1* and *OsbZIP81.2* in different tissues at maturation stage, including root, internode, sheath, leaf, glume and seeds.

**Supplementary Figure 5. Expression identification of OsbZIP81.1ox and OsbZIP81.2ox.**

**A**


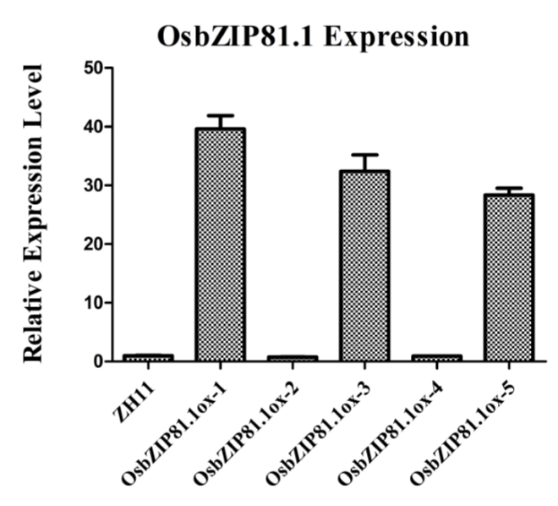


**B**


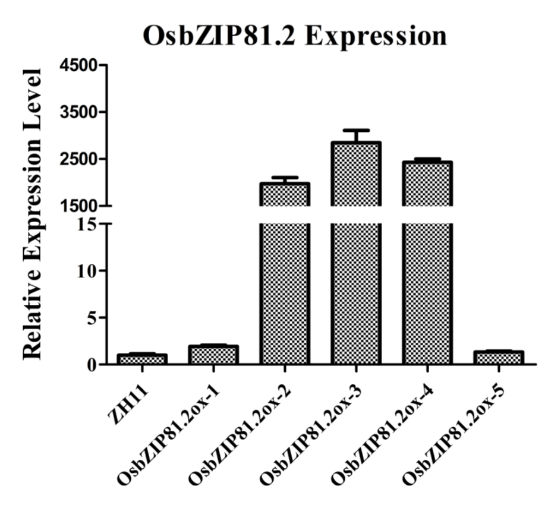


**(A)** The expression of *OsbZIP81.1* in OsbZIP81.1ox transgenic rice lines. **(B)** The expression of *OsbZIP81.2* in OsbZIP81.2ox transgenic rice lines.


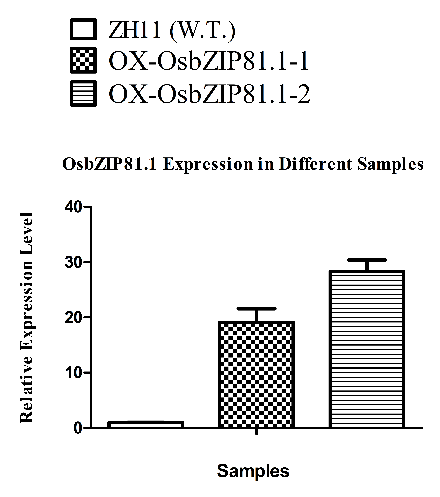


**A**


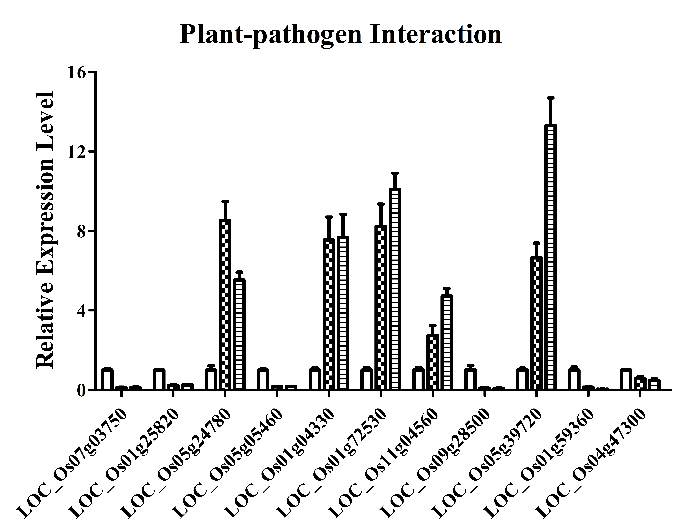


**B**


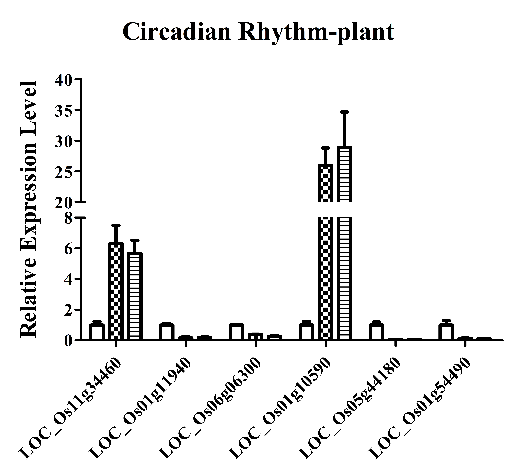


**F**


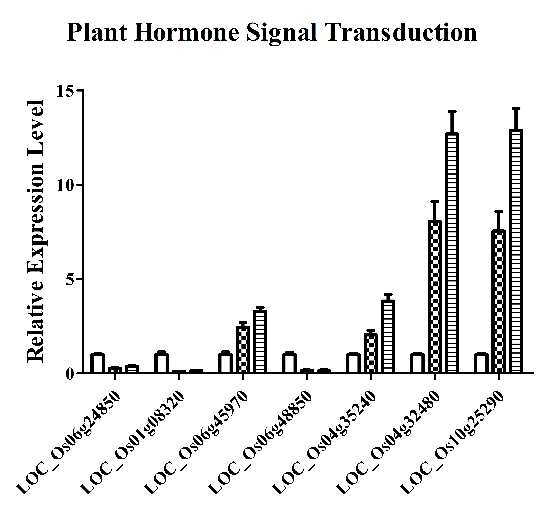


**G**


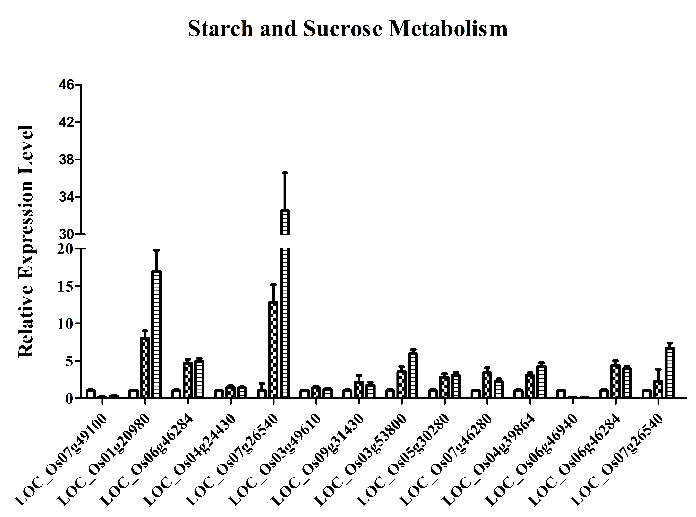


**H**


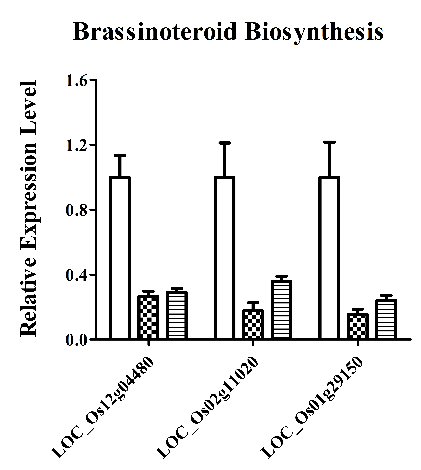


**D**


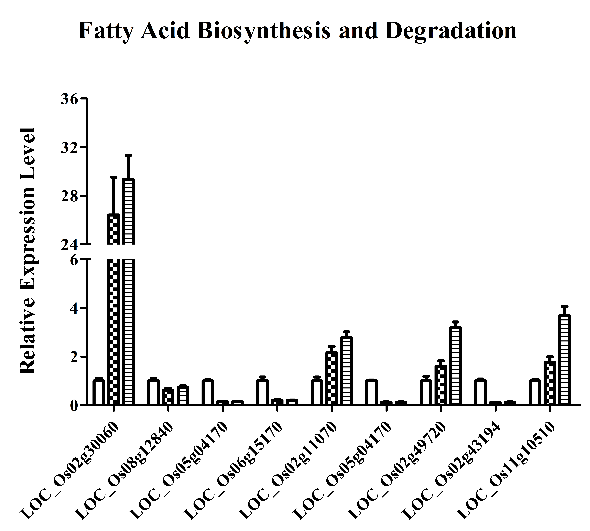


**C**


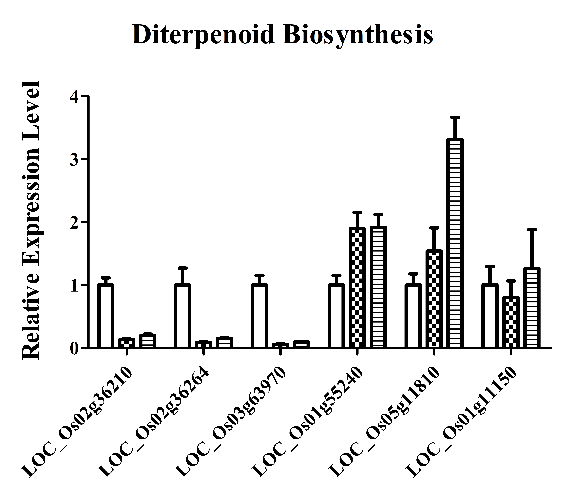


**E**

**Supplementary Figure 6. Validation of differential expressed genes in RNA-Seq by qRT-PCR.**


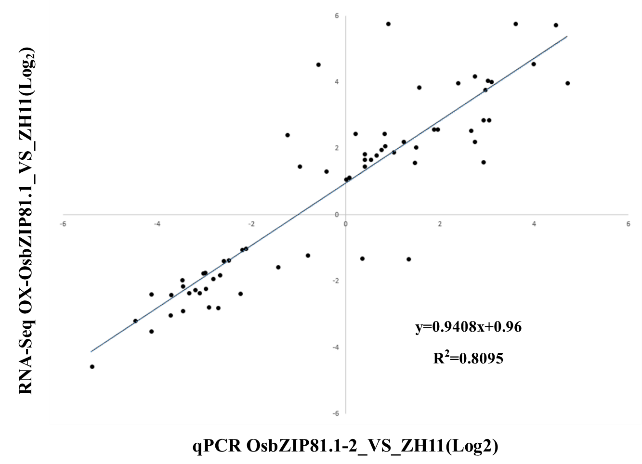

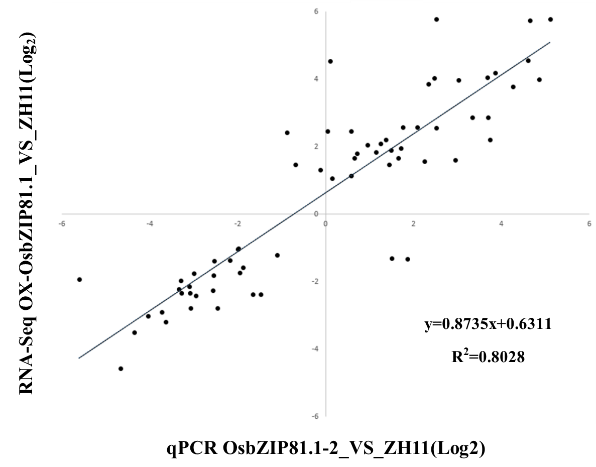


**I**

**(A)** The expressions of *OsbZIP81.1* in wild-type ZH11 and two OsbZIP81.1-overexpression samples (mixed with three lines of OsbZIP81.1ox, renamed as OX-OsbZIP81.1-1 and OX-OsbZIP81.1-2). **(B)** Eleven genes associated with plant-pathogen interaction. **(C)** Nine genes related to fatty acid biosynthesis and degradation mechanisms. **(D)** Three genes related to brassinosteroid biosynthesis. **(E)** Six genes related to diterpenoid biosynthesis. **(F)** Six genes related to circadian rhythm-plant mechanisms. **(G)** Eight genes involved in plant hormone signal transduction mechanisms. **(H)** Fourteen genes involved in starch and sucrose metabolism. **(I)** Comparison between the gene expression data obtained from RNA-Seq (y=axis; log_2_) and qRT-PCR (x-axis; log_2_).

**Supplementary Figure 7.** **Validation of the OsbZIP81.1 binding sites of partial genes by ChIP-qPCR.**


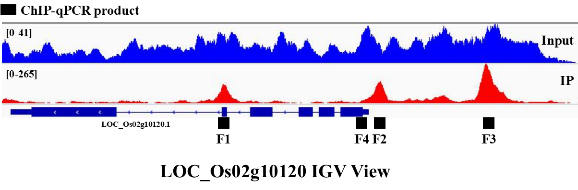


**A**


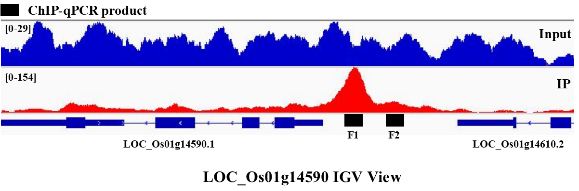


**E**


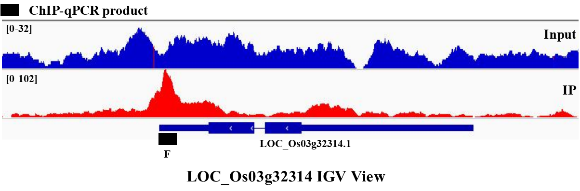


**B**


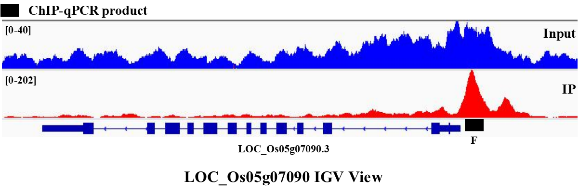


**C**


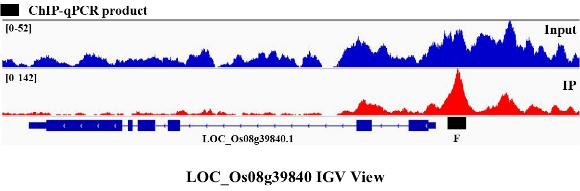


**D**


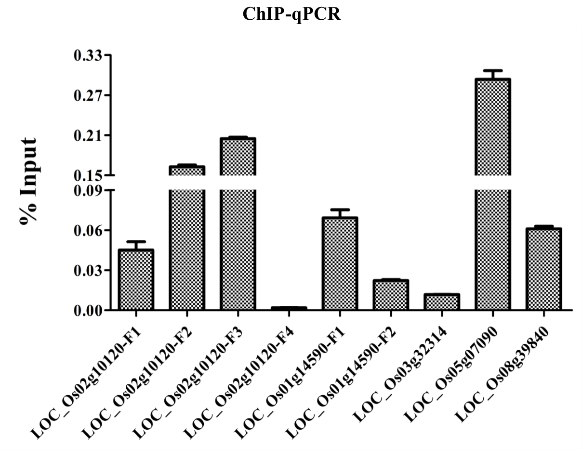


**F**

**(A～E)** The peaks in ChIP-Seq library showed in IGV window. The black boxes indicate the sequences used in ChIP-qPCR. these peaks were located at the promoter and CDS regions of the LOC_Os02g10120, at the 3’ UTR region of the LOC_Os03g32314, at the promoter regions of the LOC_Os05g07090, at the promoter region of the LOC_Os08g39840, and at the promoter region of the LOC_Os01g14590. **(E)** Validation of these peaks by ChIP-qPCR. The values in each column are the means of three independent replicates and error bars represent the SEM.


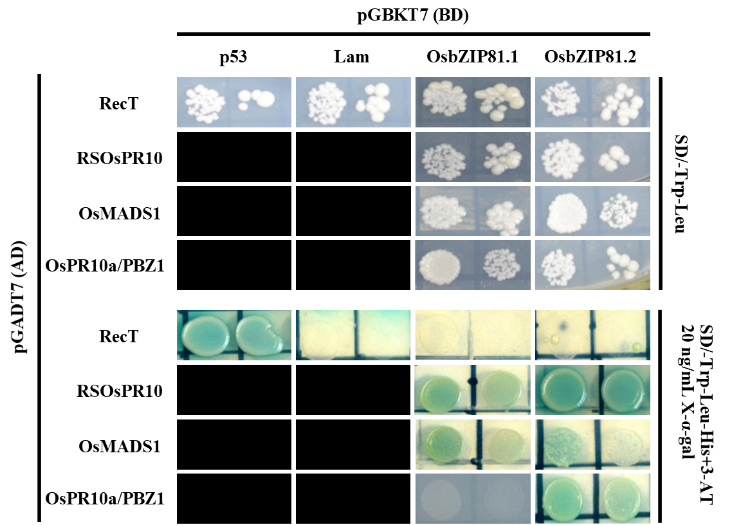
**Supplementary Figure 8. The interaction relationships between OsbZIP81.1 (or OsbZIP81.2) and the deduced proteins of the Y2H screened genes.**

In Y2H, the concentrations of 3-AT for BD-OsbZIP81.1 and OsbZIP81.2 are 80 mmol/L and 5 mmol/L, respectively.

**Supplementary Figure 9. The phenotype of *osbzip81* mutant.**


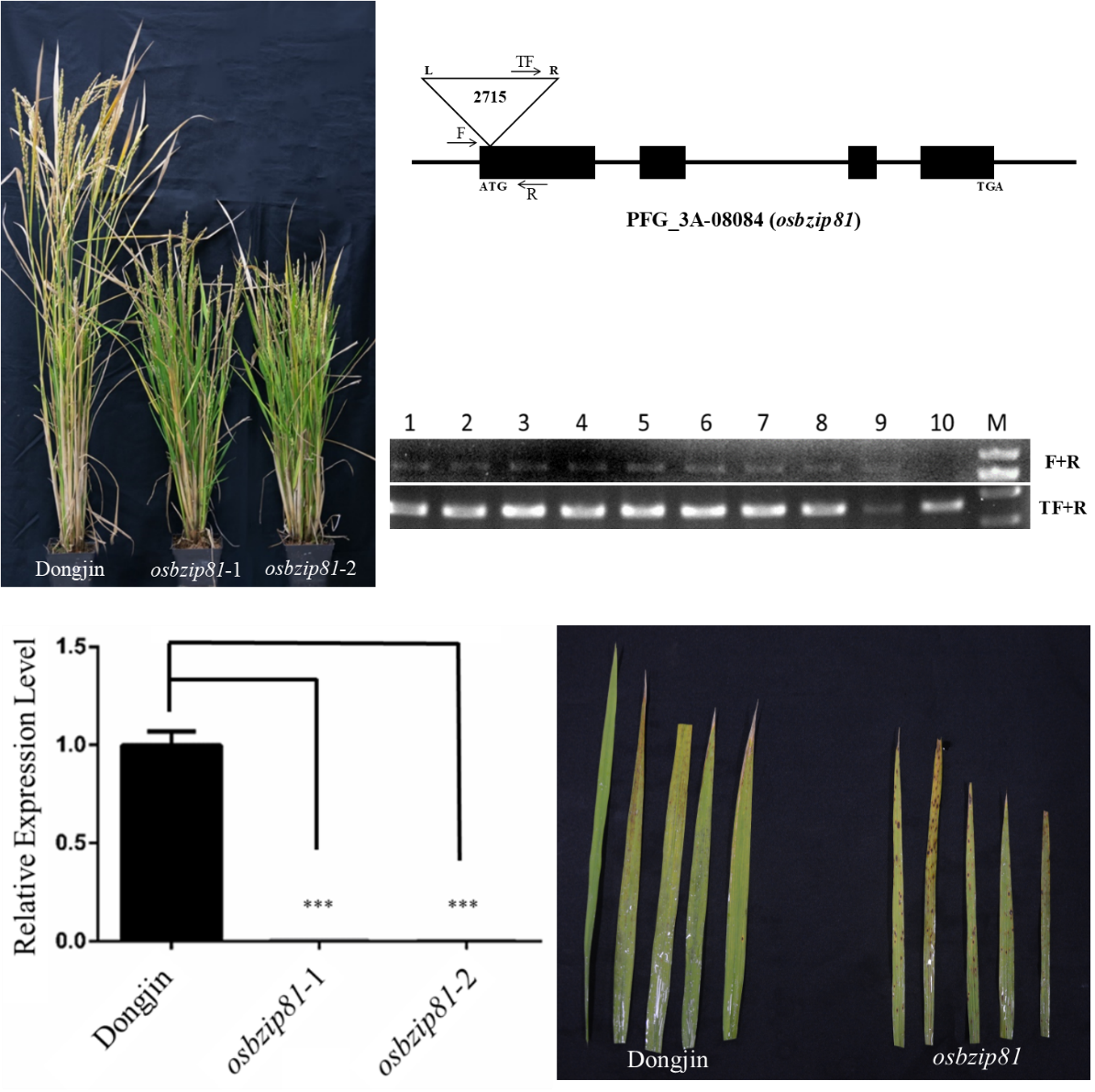


**D**

**A**

**C**

**E**

**B**

**(A)** The phenotypes of the *osbzip81* mutant and wild type. **(B)** The gene structure of the *osbzip81* mutant. **(C)** The genotype detection result of the *osbzip81* mutant. **(D)** The expression levels of *OsbZIP81* in the *osbzip81* mutant and wild type. **(E)** The leaf phenotypes of the *osbzip81* mutant and wild type.
