# Supplementary material for: OsbZIP81, A Homologue of Arabidopsis VIP1, May Positively Regulate JA Levels by Directly Targetting the Genes in JA Signaling and Metabolism Pathway in Rice
Source: Int J Mol Sci. 2019 May 13;20(9):2360. doi: 10.3390/ijms20092360 (PMC6539606; doi:10.3390/ijms20092360)
Supplement: Supplementary file 1 [file ijms-20-02360-s001.zip › Supplementary File 9.docx]

**Supplemental File 9**. List of genes in each of the enriched gene ontology (GO) categories.

GO_acc term_type Term queryitem querytotal bgitem bgtotal pvalue FDR entries

GO:0009889 P regulation of biosynthetic process 101 778 1616 24460 2.6e-10 3.1e-08 //

LOC_Os06g43090 // LOC_Os01g50720 // LOC_Os01g74020 // LOC_Os01g64790 // LOC_Os03g42630 // LOC_Os04g57720 // LOC_Os05g04210 // LOC_Os08g38210 // LOC_Os09g35010 // LOC_Os03g09170 // LOC_Os02g46940 // LOC_Os03g19120 // LOC_Os05g37080 // LOC_Os02g45170 // LOC_Os03g55990 // LOC_Os11g02470 // LOC_Os06g10780 // LOC_Os05g46610 // LOC_Os03g53340 // LOC_Os03g06630 // LOC_Os12g40920 // LOC_Os01g51140 // LOC_Os01g60600 // LOC_Os05g10620 // LOC_Os01g04800 // LOC_Os01g66270 // LOC_Os02g47810 // LOC_Os02g58210 // LOC_Os08g09840 // LOC_Os01g52514 // LOC_Os03g21030 // LOC_Os11g01480 // LOC_Os04g46020 // LOC_Os12g01490 // LOC_Os01g09100 // LOC_Os01g21120 // LOC_Os12g41680 // LOC_Os06g10880 // LOC_Os01g62310 // LOC_Os09g35030 // LOC_Os09g28354 // LOC_Os09g28210 // LOC_Os02g43790 // LOC_Os03g03164 // LOC_Os01g09590 // LOC_Os12g02400 // LOC_Os05g49100 // LOC_Os06g03670 // LOC_Os09g25060 // LOC_Os06g41060 // LOC_Os11g29870 // LOC_Os08g43334 // LOC_Os10g42490 // LOC_Os09g35020 // LOC_Os04g32620 // LOC_Os04g23550 // LOC_Os01g55340 // LOC_Os08g41320 // LOC_Os02g09480 // LOC_Os11g03300 // LOC_Os01g01840 // LOC_Os02g36880 // LOC_Os07g02060 // LOC_Os01g50940 // LOC_Os01g06640 // LOC_Os04g40630 // LOC_Os02g08440 // LOC_Os02g42850 // LOC_Os01g58760 // LOC_Os01g60020 // LOC_Os11g02520 // LOC_Os08g43210 // LOC_Os02g46500 // LOC_Os01g12440 // LOC_Os11g06170 // LOC_Os01g68020 // LOC_Os01g62950 // LOC_Os01g18584 // LOC_Os04g40140 // LOC_Os02g04640 // LOC_Os08g02300 // LOC_Os02g45450 // LOC_Os04g41570 // LOC_Os02g38130 // LOC_Os10g42230 // LOC_Os09g28440 // LOC_Os03g53150 // LOC_Os03g55590 // LOC_Os02g45420 // LOC_Os11g45740 // LOC_Os09g36910 // LOC_Os08g34360 // LOC_Os01g56690 // LOC_Os06g49040 // LOC_Os07g43530 // LOC_Os03g20090 // LOC_Os04g58190 // LOC_Os05g08570 // LOC_Os03g59670 // LOC_Os01g06740 // LOC_Os08g09900

GO:0010556 P regulation of macromolecule biosynthetic process 101 778 1616 24460 2.6e-10 3.1e-08 //

LOC_Os06g43090 // LOC_Os01g50720 // LOC_Os01g74020 // LOC_Os01g64790 // LOC_Os03g42630 // LOC_Os04g57720 // LOC_Os05g04210 // LOC_Os08g38210 // LOC_Os09g35010 // LOC_Os03g09170 // LOC_Os02g46940 // LOC_Os03g19120 // LOC_Os05g37080 // LOC_Os02g45170 // LOC_Os03g55990 // LOC_Os11g02470 // LOC_Os06g10780 // LOC_Os05g46610 // LOC_Os03g53340 // LOC_Os03g06630 // LOC_Os12g40920 // LOC_Os01g51140 // LOC_Os01g60600 // LOC_Os05g10620 // LOC_Os01g04800 // LOC_Os01g66270 // LOC_Os02g47810 // LOC_Os02g58210 // LOC_Os08g09840 // LOC_Os01g52514 // LOC_Os03g21030 // LOC_Os11g01480 // LOC_Os04g46020 // LOC_Os12g01490 // LOC_Os01g09100 // LOC_Os01g21120 // LOC_Os12g41680 // LOC_Os06g10880 // LOC_Os01g62310 // LOC_Os09g35030 // LOC_Os09g28354 // LOC_Os09g28210 // LOC_Os02g43790 // LOC_Os03g03164 // LOC_Os01g09590 // LOC_Os12g02400 // LOC_Os05g49100 // LOC_Os06g03670 // LOC_Os09g25060 // LOC_Os06g41060 // LOC_Os11g29870 // LOC_Os08g43334 // LOC_Os10g42490 // LOC_Os09g35020 // LOC_Os04g32620 // LOC_Os04g23550 // LOC_Os01g55340 // LOC_Os08g41320 // LOC_Os02g09480 // LOC_Os11g03300 // LOC_Os01g01840 // LOC_Os02g36880 // LOC_Os07g02060 // LOC_Os01g50940 // LOC_Os01g06640 // LOC_Os04g40630 // LOC_Os02g08440 // LOC_Os02g42850 // LOC_Os01g58760 // LOC_Os01g60020 // LOC_Os11g02520 // LOC_Os08g43210 // LOC_Os02g46500 // LOC_Os01g12440 // LOC_Os11g06170 // LOC_Os01g68020 // LOC_Os01g62950 // LOC_Os01g18584 // LOC_Os04g40140 // LOC_Os02g04640 // LOC_Os08g02300 // LOC_Os02g45450 // LOC_Os04g41570 // LOC_Os02g38130 // LOC_Os10g42230 // LOC_Os09g28440 // LOC_Os03g53150 // LOC_Os03g55590 // LOC_Os02g45420 // LOC_Os11g45740 // LOC_Os09g36910 // LOC_Os08g34360 // LOC_Os01g56690 // LOC_Os06g49040 // LOC_Os07g43530 // LOC_Os03g20090 // LOC_Os04g58190 // LOC_Os05g08570 // LOC_Os03g59670 // LOC_Os01g06740 // LOC_Os08g09900

GO:0031326 P regulation of cellular biosynthetic process 101 778 1616 24460 2.6e-10 3.1e-08 //

LOC_Os06g43090 // LOC_Os01g50720 // LOC_Os01g74020 // LOC_Os01g64790 // LOC_Os03g42630 // LOC_Os04g57720 // LOC_Os05g04210 // LOC_Os08g38210 // LOC_Os09g35010 // LOC_Os03g09170 // LOC_Os02g46940 // LOC_Os03g19120 // LOC_Os05g37080 // LOC_Os02g45170 // LOC_Os03g55990 // LOC_Os11g02470 // LOC_Os06g10780 // LOC_Os05g46610 // LOC_Os03g53340 // LOC_Os03g06630 // LOC_Os12g40920 // LOC_Os01g51140 // LOC_Os01g60600 // LOC_Os05g10620 // LOC_Os01g04800 // LOC_Os01g66270 // LOC_Os02g47810 // LOC_Os02g58210 // LOC_Os08g09840 // LOC_Os01g52514 // LOC_Os03g21030 // LOC_Os11g01480 // LOC_Os04g46020 // LOC_Os12g01490 // LOC_Os01g09100 // LOC_Os01g21120 // LOC_Os12g41680 // LOC_Os06g10880 // LOC_Os01g62310 // LOC_Os09g35030 // LOC_Os09g28354 // LOC_Os09g28210 // LOC_Os02g43790 // LOC_Os03g03164 // LOC_Os01g09590 // LOC_Os12g02400 // LOC_Os05g49100 // LOC_Os06g03670 // LOC_Os09g25060 // LOC_Os06g41060 // LOC_Os11g29870 // LOC_Os08g43334 // LOC_Os10g42490 // LOC_Os09g35020 // LOC_Os04g32620 // LOC_Os04g23550 // LOC_Os01g55340 // LOC_Os08g41320 // LOC_Os02g09480 // LOC_Os11g03300 // LOC_Os01g01840 // LOC_Os02g36880 // LOC_Os07g02060 // LOC_Os01g50940 // LOC_Os01g06640 // LOC_Os04g40630 // LOC_Os02g08440 // LOC_Os02g42850 // LOC_Os01g58760 // LOC_Os01g60020 // LOC_Os11g02520 // LOC_Os08g43210 // LOC_Os02g46500 // LOC_Os01g12440 // LOC_Os11g06170 // LOC_Os01g68020 // LOC_Os01g62950 // LOC_Os01g18584 // LOC_Os04g40140 // LOC_Os02g04640 // LOC_Os08g02300 // LOC_Os02g45450 // LOC_Os04g41570 // LOC_Os02g38130 // LOC_Os10g42230 // LOC_Os09g28440 // LOC_Os03g53150 // LOC_Os03g55590 // LOC_Os02g45420 // LOC_Os11g45740 // LOC_Os09g36910 // LOC_Os08g34360 // LOC_Os01g56690 // LOC_Os06g49040 // LOC_Os07g43530 // LOC_Os03g20090 // LOC_Os04g58190 // LOC_Os05g08570 // LOC_Os03g59670 // LOC_Os01g06740 // LOC_Os08g09900

GO:0031323 P regulation of cellular metabolic process 102 778 1639 24460 2.6e-10 3.1e-08 //

LOC_Os06g43090 // LOC_Os01g50720 // LOC_Os01g74020 // LOC_Os01g64790 // LOC_Os03g42630 // LOC_Os04g57720 // LOC_Os05g04210 // LOC_Os08g38210 // LOC_Os09g35010 // LOC_Os03g09170 // LOC_Os02g46940 // LOC_Os03g19120 // LOC_Os05g37080 // LOC_Os02g45170 // LOC_Os03g55990 // LOC_Os11g02470 // LOC_Os06g10780 // LOC_Os05g46610 // LOC_Os03g53340 // LOC_Os03g06630 // LOC_Os12g40920 // LOC_Os01g51140 // LOC_Os01g60600 // LOC_Os05g10620 // LOC_Os01g04800 // LOC_Os01g66270 // LOC_Os02g47810 // LOC_Os02g58210 // LOC_Os08g09840 // LOC_Os01g52514 // LOC_Os03g21030 // LOC_Os11g01480 // LOC_Os04g46020 // LOC_Os12g01490 // LOC_Os01g09100 // LOC_Os01g21120 // LOC_Os12g41680 // LOC_Os06g10880 // LOC_Os01g62310 // LOC_Os09g35030 // LOC_Os09g28354 // LOC_Os09g28210 // LOC_Os02g43790 // LOC_Os03g03164 // LOC_Os01g09590 // LOC_Os12g02400 // LOC_Os05g49100 // LOC_Os06g03670 // LOC_Os09g25060 // LOC_Os06g41060 // LOC_Os11g29870 // LOC_Os08g43334 // LOC_Os10g42490 // LOC_Os09g35020 // LOC_Os04g32620 // LOC_Os04g23550 // LOC_Os01g55340 // LOC_Os08g41320 // LOC_Os02g09480 // LOC_Os11g03300 // LOC_Os01g01840 // LOC_Os02g36880 // LOC_Os07g02060 // LOC_Os01g50940 // LOC_Os01g06640 // LOC_Os04g40630 // LOC_Os02g08440 // LOC_Os02g42850 // LOC_Os01g58760 // LOC_Os01g60020 // LOC_Os11g02520 // LOC_Os08g43210 // LOC_Os02g46500 // LOC_Os01g12440 // LOC_Os11g06170 // LOC_Os01g68020 // LOC_Os01g62950 // LOC_Os01g18584 // LOC_Os04g40140 // LOC_Os02g04640 // LOC_Os08g02300 // LOC_Os02g45450 // LOC_Os04g41570 // LOC_Os02g38130 // LOC_Os05g08570 // LOC_Os10g42230 // LOC_Os09g28440 // LOC_Os03g53150 // LOC_Os03g55590 // LOC_Os02g45420 // LOC_Os11g45740 // LOC_Os09g36910 // LOC_Os08g34360 // LOC_Os01g56690 // LOC_Os06g49040 // LOC_Os07g43530 // LOC_Os03g20090 // LOC_Os04g58190 // LOC_Os01g31690 // LOC_Os03g59670 // LOC_Os01g06740 // LOC_Os08g09900

GO:0045449 P regulation of transcription 100 778 1576 24460 1.5e-10 3.1e-08 //

LOC_Os06g43090 // LOC_Os01g50720 // LOC_Os01g74020 // LOC_Os01g64790 // LOC_Os03g42630 // LOC_Os04g57720 // LOC_Os05g04210 // LOC_Os08g38210 // LOC_Os09g35010 // LOC_Os03g09170 // LOC_Os02g46940 // LOC_Os03g19120 // LOC_Os05g37080 // LOC_Os02g45170 // LOC_Os03g55990 // LOC_Os11g02470 // LOC_Os06g10780 // LOC_Os05g46610 // LOC_Os03g53340 // LOC_Os03g06630 // LOC_Os12g40920 // LOC_Os01g51140 // LOC_Os01g60600 // LOC_Os05g10620 // LOC_Os01g04800 // LOC_Os01g66270 // LOC_Os02g47810 // LOC_Os02g58210 // LOC_Os08g09840 // LOC_Os01g52514 // LOC_Os03g21030 // LOC_Os11g01480 // LOC_Os04g46020 // LOC_Os12g01490 // LOC_Os01g09100 // LOC_Os01g21120 // LOC_Os12g41680 // LOC_Os06g10880 // LOC_Os01g62310 // LOC_Os09g35030 // LOC_Os09g28354 // LOC_Os09g28210 // LOC_Os02g43790 // LOC_Os03g03164 // LOC_Os01g09590 // LOC_Os12g02400 // LOC_Os05g49100 // LOC_Os06g03670 // LOC_Os09g25060 // LOC_Os06g41060 // LOC_Os11g29870 // LOC_Os08g43334 // LOC_Os10g42490 // LOC_Os09g35020 // LOC_Os04g32620 // LOC_Os04g23550 // LOC_Os01g55340 // LOC_Os08g41320 // LOC_Os02g09480 // LOC_Os11g03300 // LOC_Os01g01840 // LOC_Os02g36880 // LOC_Os07g02060 // LOC_Os01g50940 // LOC_Os01g06640 // LOC_Os04g40630 // LOC_Os02g08440 // LOC_Os02g42850 // LOC_Os01g58760 // LOC_Os01g60020 // LOC_Os11g02520 // LOC_Os08g43210 // LOC_Os02g46500 // LOC_Os01g12440 // LOC_Os11g06170 // LOC_Os01g68020 // LOC_Os01g62950 // LOC_Os01g18584 // LOC_Os04g40140 // LOC_Os02g04640 // LOC_Os08g02300 // LOC_Os02g45450 // LOC_Os04g41570 // LOC_Os02g38130 // LOC_Os10g42230 // LOC_Os09g28440 // LOC_Os03g53150 // LOC_Os03g55590 // LOC_Os02g45420 // LOC_Os11g45740 // LOC_Os09g36910 // LOC_Os08g34360 // LOC_Os01g56690 // LOC_Os06g49040 // LOC_Os07g43530 // LOC_Os03g20090 // LOC_Os04g58190 // LOC_Os05g08570 // LOC_Os03g59670 // LOC_Os08g09900

GO:0019219 P regulation of nucleobase, nucleoside, nucleotide and nucleic acid metabolic process 100 778 1582 24460 1.8e-10 3.1e-08 //

LOC_Os06g43090 // LOC_Os01g50720 // LOC_Os01g74020 // LOC_Os01g64790 // LOC_Os03g42630 // LOC_Os04g57720 // LOC_Os05g04210 // LOC_Os08g38210 // LOC_Os09g35010 // LOC_Os03g09170 // LOC_Os02g46940 // LOC_Os03g19120 // LOC_Os05g37080 // LOC_Os02g45170 // LOC_Os03g55990 // LOC_Os11g02470 // LOC_Os06g10780 // LOC_Os05g46610 // LOC_Os03g53340 // LOC_Os03g06630 // LOC_Os12g40920 // LOC_Os01g51140 // LOC_Os01g60600 // LOC_Os05g10620 // LOC_Os01g04800 // LOC_Os01g66270 // LOC_Os02g47810 // LOC_Os02g58210 // LOC_Os08g09840 // LOC_Os01g52514 // LOC_Os03g21030 // LOC_Os11g01480 // LOC_Os04g46020 // LOC_Os12g01490 // LOC_Os01g09100 // LOC_Os01g21120 // LOC_Os12g41680 // LOC_Os06g10880 // LOC_Os01g62310 // LOC_Os09g35030 // LOC_Os09g28354 // LOC_Os09g28210 // LOC_Os02g43790 // LOC_Os03g03164 // LOC_Os01g09590 // LOC_Os12g02400 // LOC_Os05g49100 // LOC_Os06g03670 // LOC_Os09g25060 // LOC_Os06g41060 // LOC_Os11g29870 // LOC_Os08g43334 // LOC_Os10g42490 // LOC_Os09g35020 // LOC_Os04g32620 // LOC_Os04g23550 // LOC_Os01g55340 // LOC_Os08g41320 // LOC_Os02g09480 // LOC_Os11g03300 // LOC_Os01g01840 // LOC_Os02g36880 // LOC_Os07g02060 // LOC_Os01g50940 // LOC_Os01g06640 // LOC_Os04g40630 // LOC_Os02g08440 // LOC_Os02g42850 // LOC_Os01g58760 // LOC_Os01g60020 // LOC_Os11g02520 // LOC_Os08g43210 // LOC_Os02g46500 // LOC_Os01g12440 // LOC_Os11g06170 // LOC_Os01g68020 // LOC_Os01g62950 // LOC_Os01g18584 // LOC_Os04g40140 // LOC_Os02g04640 // LOC_Os08g02300 // LOC_Os02g45450 // LOC_Os04g41570 // LOC_Os02g38130 // LOC_Os10g42230 // LOC_Os09g28440 // LOC_Os03g53150 // LOC_Os03g55590 // LOC_Os02g45420 // LOC_Os11g45740 // LOC_Os09g36910 // LOC_Os08g34360 // LOC_Os01g56690 // LOC_Os06g49040 // LOC_Os07g43530 // LOC_Os03g20090 // LOC_Os04g58190 // LOC_Os05g08570 // LOC_Os03g59670 // LOC_Os08g09900

GO:0051171 P regulation of nitrogen compound metabolic process 100 778 1582 24460 1.8e-10 3.1e-08 //

LOC_Os06g43090 // LOC_Os01g50720 // LOC_Os01g74020 // LOC_Os01g64790 // LOC_Os03g42630 // LOC_Os04g57720 // LOC_Os05g04210 // LOC_Os08g38210 // LOC_Os09g35010 // LOC_Os03g09170 // LOC_Os02g46940 // LOC_Os03g19120 // LOC_Os05g37080 // LOC_Os02g45170 // LOC_Os03g55990 // LOC_Os11g02470 // LOC_Os06g10780 // LOC_Os05g46610 // LOC_Os03g53340 // LOC_Os03g06630 // LOC_Os12g40920 // LOC_Os01g51140 // LOC_Os01g60600 // LOC_Os05g10620 // LOC_Os01g04800 // LOC_Os01g66270 // LOC_Os02g47810 // LOC_Os02g58210 // LOC_Os08g09840 // LOC_Os01g52514 // LOC_Os03g21030 // LOC_Os11g01480 // LOC_Os04g46020 // LOC_Os12g01490 // LOC_Os01g09100 // LOC_Os01g21120 // LOC_Os12g41680 // LOC_Os06g10880 // LOC_Os01g62310 // LOC_Os09g35030 // LOC_Os09g28354 // LOC_Os09g28210 // LOC_Os02g43790 // LOC_Os03g03164 // LOC_Os01g09590 // LOC_Os12g02400 // LOC_Os05g49100 // LOC_Os06g03670 // LOC_Os09g25060 // LOC_Os06g41060 // LOC_Os11g29870 // LOC_Os08g43334 // LOC_Os10g42490 // LOC_Os09g35020 // LOC_Os04g32620 // LOC_Os04g23550 // LOC_Os01g55340 // LOC_Os08g41320 // LOC_Os02g09480 // LOC_Os11g03300 // LOC_Os01g01840 // LOC_Os02g36880 // LOC_Os07g02060 // LOC_Os01g50940 // LOC_Os01g06640 // LOC_Os04g40630 // LOC_Os02g08440 // LOC_Os02g42850 // LOC_Os01g58760 // LOC_Os01g60020 // LOC_Os11g02520 // LOC_Os08g43210 // LOC_Os02g46500 // LOC_Os01g12440 // LOC_Os11g06170 // LOC_Os01g68020 // LOC_Os01g62950 // LOC_Os01g18584 // LOC_Os04g40140 // LOC_Os02g04640 // LOC_Os08g02300 // LOC_Os02g45450 // LOC_Os04g41570 // LOC_Os02g38130 // LOC_Os10g42230 // LOC_Os09g28440 // LOC_Os03g53150 // LOC_Os03g55590 // LOC_Os02g45420 // LOC_Os11g45740 // LOC_Os09g36910 // LOC_Os08g34360 // LOC_Os01g56690 // LOC_Os06g49040 // LOC_Os07g43530 // LOC_Os03g20090 // LOC_Os04g58190 // LOC_Os05g08570 // LOC_Os03g59670 // LOC_Os08g09900

GO:0005975 P carbohydrate metabolic process 67 778 864 24460 1.2e-10 3.1e-08 //

LOC_Os08g41100 // LOC_Os01g71474 // LOC_Os11g43750 // LOC_Os09g29600 // LOC_Os07g46280 // LOC_Os01g70170 // LOC_Os03g59340 // LOC_Os01g61380 // LOC_Os01g39830 // LOC_Os10g39680 // LOC_Os09g31270 // LOC_Os04g39900 // LOC_Os06g25010 // LOC_Os02g37590 // LOC_Os09g12660 // LOC_Os04g40874 // LOC_Os03g55090 // LOC_Os04g51460 // LOC_Os06g51060 // LOC_Os01g64170 // LOC_Os06g48200 // LOC_Os05g15510 // LOC_Os06g48160 // LOC_Os04g41970 // LOC_Os02g47020 // LOC_Os04g39880 // LOC_Os03g49610 // LOC_Os01g03950 // LOC_Os05g08370 // LOC_Os09g29404 // LOC_Os12g05110 // LOC_Os06g06560 // LOC_Os02g05744 // LOC_Os03g13570 // LOC_Os05g32710 // LOC_Os05g15770 // LOC_Os06g46284 // LOC_Os09g32080 // LOC_Os03g25790 // LOC_Os01g54620 // LOC_Os05g44210 // LOC_Os06g51050 // LOC_Os10g39700 // LOC_Os05g31140 // LOC_Os10g41550 // LOC_Os08g33720 // LOC_Os09g36350 // LOC_Os02g46910 // LOC_Os11g37970 // LOC_Os10g35070 // LOC_Os11g07020 // LOC_Os11g37950 // LOC_Os03g53800 // LOC_Os06g40640 // LOC_Os01g71810 // LOC_Os09g23084 // LOC_Os01g43160 // LOC_Os05g41590 // LOC_Os02g48360 // LOC_Os06g46940 // LOC_Os02g50490 // LOC_Os08g34050 // LOC_Os07g10770 // LOC_Os09g33690 // LOC_Os03g12140 // LOC_Os04g52730 // LOC_Os03g49600

GO:0065007 P biological regulation 124 778 2175 24460 4.6e-10 4.4e-08 //

LOC_Os06g43090 // LOC_Os01g50720 // LOC_Os01g74020 // LOC_Os01g64790 // LOC_Os03g42630 // LOC_Os11g09280 // LOC_Os03g02750 // LOC_Os05g04210 // LOC_Os08g38210 // LOC_Os09g35010 // LOC_Os03g09170 // LOC_Os02g46940 // LOC_Os03g29240 // LOC_Os03g19120 // LOC_Os05g37080 // LOC_Os03g55990 // LOC_Os11g02470 // LOC_Os06g10780 // LOC_Os05g46610 // LOC_Os03g53340 // LOC_Os03g06630 // LOC_Os01g52750 // LOC_Os01g51140 // LOC_Os01g60600 // LOC_Os05g10620 // LOC_Os01g04800 // LOC_Os01g66270 // LOC_Os02g47810 // LOC_Os02g45170 // LOC_Os03g22780 // LOC_Os08g41340 // LOC_Os02g06480 // LOC_Os08g09840 // LOC_Os01g52514 // LOC_Os03g21030 // LOC_Os11g01480 // LOC_Os04g57720 // LOC_Os04g46020 // LOC_Os12g01490 // LOC_Os06g28550 // LOC_Os01g09100 // LOC_Os01g21120 // LOC_Os12g41680 // LOC_Os06g10880 // LOC_Os01g62310 // LOC_Os09g35030 // LOC_Os09g28354 // LOC_Os09g28210 // LOC_Os02g43790 // LOC_Os11g01530 // LOC_Os01g09590 // LOC_Os12g02400 // LOC_Os05g49100 // LOC_Os06g03670 // LOC_Os09g25060 // LOC_Os06g41060 // LOC_Os11g29870 // LOC_Os08g43334 // LOC_Os10g42490 // LOC_Os09g35020 // LOC_Os04g32620 // LOC_Os04g23550 // LOC_Os01g55340 // LOC_Os11g34460 // LOC_Os08g41320 // LOC_Os02g09480 // LOC_Os11g03300 // LOC_Os01g01840 // LOC_Os07g48760 // LOC_Os02g36880 // LOC_Os07g02060 // LOC_Os01g50940 // LOC_Os01g06640 // LOC_Os04g40630 // LOC_Os06g06790 // LOC_Os02g08440 // LOC_Os12g01530 // LOC_Os02g42850 // LOC_Os01g58760 // LOC_Os01g60020 // LOC_Os11g02520 // LOC_Os02g58210 // LOC_Os11g02240 // LOC_Os02g51370 // LOC_Os08g43210 // LOC_Os02g46500 // LOC_Os01g12440 // LOC_Os07g29410 // LOC_Os12g02200 // LOC_Os11g06170 // LOC_Os06g12790 // LOC_Os01g68020 // LOC_Os09g30458 // LOC_Os01g62950 // LOC_Os01g18584 // LOC_Os04g40140 // LOC_Os02g04640 // LOC_Os08g02300 // LOC_Os02g45450 // LOC_Os04g41570 // LOC_Os02g38130 // LOC_Os05g48930 // LOC_Os05g08570 // LOC_Os10g42230 // LOC_Os09g28440 // LOC_Os03g53150 // LOC_Os03g18010 // LOC_Os03g21000 // LOC_Os03g55590 // LOC_Os02g45420 // LOC_Os12g40920 // LOC_Os11g45740 // LOC_Os09g36910 // LOC_Os08g34360 // LOC_Os01g56690 // LOC_Os06g49040 // LOC_Os07g43530 // LOC_Os03g03164 // LOC_Os03g20090 // LOC_Os04g58190 // LOC_Os01g31690 // LOC_Os03g59670 // LOC_Os01g06740 // LOC_Os08g09900

GO:0050794 P regulation of cellular process 116 778 1983 24460 4.5e-10 4.4e-08 //

LOC_Os06g43090 // LOC_Os01g50720 // LOC_Os01g74020 // LOC_Os01g64790 // LOC_Os03g42630 // LOC_Os11g09280 // LOC_Os04g57720 // LOC_Os05g04210 // LOC_Os08g38210 // LOC_Os09g35010 // LOC_Os03g09170 // LOC_Os02g46940 // LOC_Os03g29240 // LOC_Os03g19120 // LOC_Os05g37080 // LOC_Os03g55990 // LOC_Os11g02470 // LOC_Os06g10780 // LOC_Os05g46610 // LOC_Os03g53340 // LOC_Os03g06630 // LOC_Os12g40920 // LOC_Os01g51140 // LOC_Os01g60600 // LOC_Os05g10620 // LOC_Os01g04800 // LOC_Os01g66270 // LOC_Os02g47810 // LOC_Os02g45170 // LOC_Os08g41340 // LOC_Os08g09840 // LOC_Os01g52514 // LOC_Os03g21030 // LOC_Os11g01480 // LOC_Os04g46020 // LOC_Os12g01490 // LOC_Os01g09100 // LOC_Os01g21120 // LOC_Os12g41680 // LOC_Os06g10880 // LOC_Os01g62310 // LOC_Os09g35030 // LOC_Os09g28354 // LOC_Os09g28210 // LOC_Os02g43790 // LOC_Os03g03164 // LOC_Os01g09590 // LOC_Os12g02400 // LOC_Os05g49100 // LOC_Os06g03670 // LOC_Os09g25060 // LOC_Os06g41060 // LOC_Os11g29870 // LOC_Os08g43334 // LOC_Os10g42490 // LOC_Os09g35020 // LOC_Os04g32620 // LOC_Os04g23550 // LOC_Os01g55340 // LOC_Os11g34460 // LOC_Os08g41320 // LOC_Os02g09480 // LOC_Os11g03300 // LOC_Os01g01840 // LOC_Os07g48760 // LOC_Os02g36880 // LOC_Os07g02060 // LOC_Os01g50940 // LOC_Os01g06640 // LOC_Os04g40630 // LOC_Os06g06790 // LOC_Os02g08440 // LOC_Os02g42850 // LOC_Os01g58760 // LOC_Os01g60020 // LOC_Os11g02520 // LOC_Os02g58210 // LOC_Os11g02240 // LOC_Os02g51370 // LOC_Os08g43210 // LOC_Os02g46500 // LOC_Os01g12440 // LOC_Os07g29410 // LOC_Os12g02200 // LOC_Os11g06170 // LOC_Os06g12790 // LOC_Os01g68020 // LOC_Os01g62950 // LOC_Os01g18584 // LOC_Os04g40140 // LOC_Os02g04640 // LOC_Os08g02300 // LOC_Os02g45450 // LOC_Os04g41570 // LOC_Os02g38130 // LOC_Os05g48930 // LOC_Os05g08570 // LOC_Os10g42230 // LOC_Os09g28440 // LOC_Os03g53150 // LOC_Os03g18010 // LOC_Os03g21000 // LOC_Os03g55590 // LOC_Os02g45420 // LOC_Os11g45740 // LOC_Os09g36910 // LOC_Os08g34360 // LOC_Os01g56690 // LOC_Os06g49040 // LOC_Os07g43530 // LOC_Os03g20090 // LOC_Os04g58190 // LOC_Os01g31690 // LOC_Os03g59670 // LOC_Os01g06740 // LOC_Os08g09900

GO:0010468 P regulation of gene expression 101 778 1638 24460 5.2e-10 4.5e-08 //

LOC_Os06g43090 // LOC_Os01g50720 // LOC_Os01g74020 // LOC_Os01g64790 // LOC_Os03g42630 // LOC_Os04g57720 // LOC_Os05g04210 // LOC_Os08g38210 // LOC_Os09g35010 // LOC_Os03g09170 // LOC_Os02g46940 // LOC_Os03g19120 // LOC_Os05g37080 // LOC_Os02g45170 // LOC_Os03g55990 // LOC_Os11g02470 // LOC_Os06g10780 // LOC_Os05g46610 // LOC_Os03g53340 // LOC_Os03g06630 // LOC_Os12g40920 // LOC_Os01g51140 // LOC_Os01g60600 // LOC_Os05g10620 // LOC_Os01g04800 // LOC_Os01g66270 // LOC_Os02g47810 // LOC_Os02g58210 // LOC_Os08g09840 // LOC_Os01g52514 // LOC_Os03g21030 // LOC_Os11g01480 // LOC_Os04g46020 // LOC_Os12g01490 // LOC_Os01g09100 // LOC_Os01g21120 // LOC_Os12g41680 // LOC_Os06g10880 // LOC_Os01g62310 // LOC_Os09g35030 // LOC_Os09g28354 // LOC_Os09g28210 // LOC_Os02g43790 // LOC_Os03g03164 // LOC_Os01g09590 // LOC_Os12g02400 // LOC_Os05g49100 // LOC_Os06g03670 // LOC_Os09g25060 // LOC_Os06g41060 // LOC_Os11g29870 // LOC_Os08g43334 // LOC_Os10g42490 // LOC_Os09g35020 // LOC_Os04g32620 // LOC_Os04g23550 // LOC_Os01g55340 // LOC_Os08g41320 // LOC_Os02g09480 // LOC_Os11g03300 // LOC_Os01g01840 // LOC_Os02g36880 // LOC_Os07g02060 // LOC_Os01g50940 // LOC_Os01g06640 // LOC_Os04g40630 // LOC_Os02g08440 // LOC_Os02g42850 // LOC_Os01g58760 // LOC_Os01g60020 // LOC_Os11g02520 // LOC_Os08g43210 // LOC_Os02g46500 // LOC_Os01g12440 // LOC_Os11g06170 // LOC_Os01g68020 // LOC_Os01g62950 // LOC_Os01g18584 // LOC_Os04g40140 // LOC_Os02g04640 // LOC_Os08g02300 // LOC_Os02g45450 // LOC_Os04g41570 // LOC_Os02g38130 // LOC_Os10g42230 // LOC_Os09g28440 // LOC_Os03g53150 // LOC_Os03g55590 // LOC_Os02g45420 // LOC_Os11g45740 // LOC_Os09g36910 // LOC_Os08g34360 // LOC_Os01g56690 // LOC_Os06g49040 // LOC_Os07g43530 // LOC_Os03g20090 // LOC_Os04g58190 // LOC_Os05g08570 // LOC_Os03g59670 // LOC_Os01g06740 // LOC_Os08g09900

GO:0080090 P regulation of primary metabolic process 101 778 1673 24460 1.5e-09 1.2e-07 //

LOC_Os06g43090 // LOC_Os01g50720 // LOC_Os01g74020 // LOC_Os01g64790 // LOC_Os03g42630 // LOC_Os04g57720 // LOC_Os05g04210 // LOC_Os08g38210 // LOC_Os09g35010 // LOC_Os03g09170 // LOC_Os02g46940 // LOC_Os03g19120 // LOC_Os05g37080 // LOC_Os02g45170 // LOC_Os03g55990 // LOC_Os11g02470 // LOC_Os06g10780 // LOC_Os05g46610 // LOC_Os03g53340 // LOC_Os03g06630 // LOC_Os12g40920 // LOC_Os01g51140 // LOC_Os01g60600 // LOC_Os05g10620 // LOC_Os01g04800 // LOC_Os01g66270 // LOC_Os02g47810 // LOC_Os02g58210 // LOC_Os08g09840 // LOC_Os01g52514 // LOC_Os03g21030 // LOC_Os11g01480 // LOC_Os04g46020 // LOC_Os12g01490 // LOC_Os01g09100 // LOC_Os01g21120 // LOC_Os12g41680 // LOC_Os06g10880 // LOC_Os01g62310 // LOC_Os09g35030 // LOC_Os09g28354 // LOC_Os09g28210 // LOC_Os02g43790 // LOC_Os03g03164 // LOC_Os01g09590 // LOC_Os12g02400 // LOC_Os05g49100 // LOC_Os06g03670 // LOC_Os09g25060 // LOC_Os06g41060 // LOC_Os11g29870 // LOC_Os08g43334 // LOC_Os10g42490 // LOC_Os09g35020 // LOC_Os04g32620 // LOC_Os04g23550 // LOC_Os01g55340 // LOC_Os08g41320 // LOC_Os02g09480 // LOC_Os11g03300 // LOC_Os01g01840 // LOC_Os02g36880 // LOC_Os07g02060 // LOC_Os01g50940 // LOC_Os01g06640 // LOC_Os04g40630 // LOC_Os02g08440 // LOC_Os02g42850 // LOC_Os01g58760 // LOC_Os01g60020 // LOC_Os11g02520 // LOC_Os08g43210 // LOC_Os02g46500 // LOC_Os01g12440 // LOC_Os11g06170 // LOC_Os01g68020 // LOC_Os01g62950 // LOC_Os01g18584 // LOC_Os04g40140 // LOC_Os02g04640 // LOC_Os08g02300 // LOC_Os02g45450 // LOC_Os04g41570 // LOC_Os02g38130 // LOC_Os10g42230 // LOC_Os09g28440 // LOC_Os03g53150 // LOC_Os03g55590 // LOC_Os02g45420 // LOC_Os11g45740 // LOC_Os09g36910 // LOC_Os08g34360 // LOC_Os01g56690 // LOC_Os06g49040 // LOC_Os07g43530 // LOC_Os03g20090 // LOC_Os04g58190 // LOC_Os05g08570 // LOC_Os03g59670 // LOC_Os01g06740 // LOC_Os08g09900

GO:0050789 P regulation of biological process 118 778 2081 24460 1.8e-09 1.3e-07 //

LOC_Os06g43090 // LOC_Os01g50720 // LOC_Os01g74020 // LOC_Os01g64790 // LOC_Os03g42630 // LOC_Os11g09280 // LOC_Os04g57720 // LOC_Os05g04210 // LOC_Os08g38210 // LOC_Os09g35010 // LOC_Os03g09170 // LOC_Os02g46940 // LOC_Os03g29240 // LOC_Os03g19120 // LOC_Os05g37080 // LOC_Os03g55990 // LOC_Os11g02470 // LOC_Os06g10780 // LOC_Os05g46610 // LOC_Os03g53340 // LOC_Os03g06630 // LOC_Os12g40920 // LOC_Os01g51140 // LOC_Os01g60600 // LOC_Os05g10620 // LOC_Os01g04800 // LOC_Os01g66270 // LOC_Os02g47810 // LOC_Os02g45170 // LOC_Os03g22780 // LOC_Os08g41340 // LOC_Os08g09840 // LOC_Os01g52514 // LOC_Os03g21030 // LOC_Os11g01480 // LOC_Os04g46020 // LOC_Os12g01490 // LOC_Os06g28550 // LOC_Os01g09100 // LOC_Os01g21120 // LOC_Os12g41680 // LOC_Os06g10880 // LOC_Os01g62310 // LOC_Os09g35030 // LOC_Os09g28354 // LOC_Os09g28210 // LOC_Os02g43790 // LOC_Os03g03164 // LOC_Os01g09590 // LOC_Os12g02400 // LOC_Os05g49100 // LOC_Os06g03670 // LOC_Os09g25060 // LOC_Os06g41060 // LOC_Os11g29870 // LOC_Os08g43334 // LOC_Os10g42490 // LOC_Os09g35020 // LOC_Os04g32620 // LOC_Os04g23550 // LOC_Os01g55340 // LOC_Os11g34460 // LOC_Os08g41320 // LOC_Os02g09480 // LOC_Os11g03300 // LOC_Os01g01840 // LOC_Os07g48760 // LOC_Os02g36880 // LOC_Os07g02060 // LOC_Os01g50940 // LOC_Os01g06640 // LOC_Os04g40630 // LOC_Os06g06790 // LOC_Os02g08440 // LOC_Os02g42850 // LOC_Os01g58760 // LOC_Os01g60020 // LOC_Os11g02520 // LOC_Os02g58210 // LOC_Os11g02240 // LOC_Os02g51370 // LOC_Os08g43210 // LOC_Os02g46500 // LOC_Os01g12440 // LOC_Os07g29410 // LOC_Os12g02200 // LOC_Os11g06170 // LOC_Os06g12790 // LOC_Os01g68020 // LOC_Os01g62950 // LOC_Os01g18584 // LOC_Os04g40140 // LOC_Os02g04640 // LOC_Os08g02300 // LOC_Os02g45450 // LOC_Os04g41570 // LOC_Os02g38130 // LOC_Os05g48930 // LOC_Os05g08570 // LOC_Os10g42230 // LOC_Os09g28440 // LOC_Os03g53150 // LOC_Os03g18010 // LOC_Os03g21000 // LOC_Os03g55590 // LOC_Os02g45420 // LOC_Os11g45740 // LOC_Os09g36910 // LOC_Os08g34360 // LOC_Os01g56690 // LOC_Os06g49040 // LOC_Os07g43530 // LOC_Os03g20090 // LOC_Os04g58190 // LOC_Os01g31690 // LOC_Os03g59670 // LOC_Os01g06740 // LOC_Os08g09900

GO:0019222 P regulation of metabolic process 102 778 1715 24460 2.6e-09 1.8e-07 //

LOC_Os06g43090 // LOC_Os01g50720 // LOC_Os01g74020 // LOC_Os01g64790 // LOC_Os03g42630 // LOC_Os04g57720 // LOC_Os05g04210 // LOC_Os08g38210 // LOC_Os09g35010 // LOC_Os03g09170 // LOC_Os02g46940 // LOC_Os03g19120 // LOC_Os05g37080 // LOC_Os02g45170 // LOC_Os03g55990 // LOC_Os11g02470 // LOC_Os06g10780 // LOC_Os05g46610 // LOC_Os03g53340 // LOC_Os03g06630 // LOC_Os12g40920 // LOC_Os01g51140 // LOC_Os01g60600 // LOC_Os05g10620 // LOC_Os01g04800 // LOC_Os01g66270 // LOC_Os02g47810 // LOC_Os02g58210 // LOC_Os08g09840 // LOC_Os01g52514 // LOC_Os03g21030 // LOC_Os11g01480 // LOC_Os04g46020 // LOC_Os12g01490 // LOC_Os01g09100 // LOC_Os01g21120 // LOC_Os12g41680 // LOC_Os06g10880 // LOC_Os01g62310 // LOC_Os09g35030 // LOC_Os09g28354 // LOC_Os09g28210 // LOC_Os02g43790 // LOC_Os03g03164 // LOC_Os01g09590 // LOC_Os12g02400 // LOC_Os05g49100 // LOC_Os06g03670 // LOC_Os09g25060 // LOC_Os06g41060 // LOC_Os11g29870 // LOC_Os08g43334 // LOC_Os10g42490 // LOC_Os09g35020 // LOC_Os04g32620 // LOC_Os04g23550 // LOC_Os01g55340 // LOC_Os08g41320 // LOC_Os02g09480 // LOC_Os11g03300 // LOC_Os01g01840 // LOC_Os02g36880 // LOC_Os07g02060 // LOC_Os01g50940 // LOC_Os01g06640 // LOC_Os04g40630 // LOC_Os02g08440 // LOC_Os02g42850 // LOC_Os01g58760 // LOC_Os01g60020 // LOC_Os11g02520 // LOC_Os08g43210 // LOC_Os02g46500 // LOC_Os01g12440 // LOC_Os11g06170 // LOC_Os01g68020 // LOC_Os01g62950 // LOC_Os01g18584 // LOC_Os04g40140 // LOC_Os02g04640 // LOC_Os08g02300 // LOC_Os02g45450 // LOC_Os04g41570 // LOC_Os02g38130 // LOC_Os05g08570 // LOC_Os10g42230 // LOC_Os09g28440 // LOC_Os03g53150 // LOC_Os03g55590 // LOC_Os02g45420 // LOC_Os11g45740 // LOC_Os09g36910 // LOC_Os08g34360 // LOC_Os01g56690 // LOC_Os06g49040 // LOC_Os07g43530 // LOC_Os03g20090 // LOC_Os04g58190 // LOC_Os01g31690 // LOC_Os03g59670 // LOC_Os01g06740 // LOC_Os08g09900

GO:0060255 P regulation of macromolecule metabolic process 101 778 1697 24460 3.1e-09 2e-07 //

LOC_Os06g43090 // LOC_Os01g50720 // LOC_Os01g74020 // LOC_Os01g64790 // LOC_Os03g42630 // LOC_Os04g57720 // LOC_Os05g04210 // LOC_Os08g38210 // LOC_Os09g35010 // LOC_Os03g09170 // LOC_Os02g46940 // LOC_Os03g19120 // LOC_Os05g37080 // LOC_Os02g45170 // LOC_Os03g55990 // LOC_Os11g02470 // LOC_Os06g10780 // LOC_Os05g46610 // LOC_Os03g53340 // LOC_Os03g06630 // LOC_Os12g40920 // LOC_Os01g51140 // LOC_Os01g60600 // LOC_Os05g10620 // LOC_Os01g04800 // LOC_Os01g66270 // LOC_Os02g47810 // LOC_Os02g58210 // LOC_Os08g09840 // LOC_Os01g52514 // LOC_Os03g21030 // LOC_Os11g01480 // LOC_Os04g46020 // LOC_Os12g01490 // LOC_Os01g09100 // LOC_Os01g21120 // LOC_Os12g41680 // LOC_Os06g10880 // LOC_Os01g62310 // LOC_Os09g35030 // LOC_Os09g28354 // LOC_Os09g28210 // LOC_Os02g43790 // LOC_Os03g03164 // LOC_Os01g09590 // LOC_Os12g02400 // LOC_Os05g49100 // LOC_Os06g03670 // LOC_Os09g25060 // LOC_Os06g41060 // LOC_Os11g29870 // LOC_Os08g43334 // LOC_Os10g42490 // LOC_Os09g35020 // LOC_Os04g32620 // LOC_Os04g23550 // LOC_Os01g55340 // LOC_Os08g41320 // LOC_Os02g09480 // LOC_Os11g03300 // LOC_Os01g01840 // LOC_Os02g36880 // LOC_Os07g02060 // LOC_Os01g50940 // LOC_Os01g06640 // LOC_Os04g40630 // LOC_Os02g08440 // LOC_Os02g42850 // LOC_Os01g58760 // LOC_Os01g60020 // LOC_Os11g02520 // LOC_Os08g43210 // LOC_Os02g46500 // LOC_Os01g12440 // LOC_Os11g06170 // LOC_Os01g68020 // LOC_Os01g62950 // LOC_Os01g18584 // LOC_Os04g40140 // LOC_Os02g04640 // LOC_Os08g02300 // LOC_Os02g45450 // LOC_Os04g41570 // LOC_Os02g38130 // LOC_Os10g42230 // LOC_Os09g28440 // LOC_Os03g53150 // LOC_Os03g55590 // LOC_Os02g45420 // LOC_Os11g45740 // LOC_Os09g36910 // LOC_Os08g34360 // LOC_Os01g56690 // LOC_Os06g49040 // LOC_Os07g43530 // LOC_Os03g20090 // LOC_Os04g58190 // LOC_Os05g08570 // LOC_Os03g59670 // LOC_Os01g06740 // LOC_Os08g09900

GO:0006350 P transcription 100 778 1698 24460 6.2e-09 3.7e-07 // LOC_Os06g43090 //

LOC_Os01g50720 // LOC_Os01g74020 // LOC_Os01g64790 // LOC_Os03g42630 // LOC_Os04g57720 // LOC_Os05g04210 // LOC_Os08g38210 // LOC_Os09g35010 // LOC_Os03g09170 // LOC_Os02g46940 // LOC_Os03g19120 // LOC_Os05g37080 // LOC_Os02g45170 // LOC_Os03g55990 // LOC_Os11g02470 // LOC_Os06g10780 // LOC_Os05g46610 // LOC_Os03g53340 // LOC_Os03g06630 // LOC_Os12g40920 // LOC_Os01g51140 // LOC_Os01g60600 // LOC_Os05g10620 // LOC_Os01g04800 // LOC_Os01g66270 // LOC_Os02g47810 // LOC_Os02g58210 // LOC_Os08g09840 // LOC_Os01g52514 // LOC_Os03g21030 // LOC_Os11g01480 // LOC_Os04g46020 // LOC_Os12g01490 // LOC_Os01g09100 // LOC_Os01g21120 // LOC_Os12g41680 // LOC_Os06g10880 // LOC_Os01g62310 // LOC_Os09g35030 // LOC_Os09g28354 // LOC_Os09g28210 // LOC_Os02g43790 // LOC_Os03g03164 // LOC_Os01g09590 // LOC_Os12g02400 // LOC_Os05g49100 // LOC_Os06g03670 // LOC_Os09g25060 // LOC_Os06g41060 // LOC_Os11g29870 // LOC_Os08g43334 // LOC_Os10g42490 // LOC_Os09g35020 // LOC_Os04g32620 // LOC_Os04g23550 // LOC_Os01g55340 // LOC_Os08g41320 // LOC_Os02g09480 // LOC_Os11g03300 // LOC_Os01g01840 // LOC_Os02g36880 // LOC_Os07g02060 // LOC_Os01g50940 // LOC_Os01g06640 // LOC_Os04g40630 // LOC_Os02g08440 // LOC_Os02g42850 // LOC_Os01g58760 // LOC_Os01g60020 // LOC_Os11g02520 // LOC_Os08g43210 // LOC_Os02g46500 // LOC_Os01g12440 // LOC_Os11g06170 // LOC_Os01g68020 // LOC_Os01g62950 // LOC_Os01g18584 // LOC_Os04g40140 // LOC_Os02g04640 // LOC_Os08g02300 // LOC_Os02g45450 // LOC_Os04g41570 // LOC_Os02g38130 // LOC_Os10g42230 // LOC_Os09g28440 // LOC_Os03g53150 // LOC_Os03g55590 // LOC_Os02g45420 // LOC_Os11g45740 // LOC_Os09g36910 // LOC_Os08g34360 // LOC_Os01g56690 // LOC_Os06g49040 // LOC_Os07g43530 // LOC_Os03g20090 // LOC_Os04g58190 // LOC_Os05g08570 // LOC_Os03g59670 // LOC_Os08g09900

GO:0005976 P polysaccharide metabolic process 20 778 140 24460 1.2e-07 6.7e-06 //

LOC_Os01g54620 // LOC_Os11g37950 // LOC_Os06g06560 // LOC_Os03g13570 // LOC_Os09g32080 // LOC_Os06g51060 // LOC_Os06g48200 // LOC_Os08g41100 // LOC_Os03g59340 // LOC_Os09g12660 // LOC_Os04g51460 // LOC_Os06g48160 // LOC_Os06g51050 // LOC_Os07g10770 // LOC_Os10g39680 // LOC_Os10g41550 // LOC_Os10g39700 // LOC_Os02g46910 // LOC_Os05g08370 // LOC_Os11g37970

GO:0006026 P aminoglycan catabolic process 8 778 26 24460 7e-06 0.00032 //

LOC_Os11g37950 // LOC_Os06g51050 // LOC_Os06g51060 // LOC_Os09g32080 // LOC_Os08g41100 // LOC_Os10g39700 // LOC_Os10g39680 // LOC_Os11g37970

GO:0006030 P chitin metabolic process 8 778 26 24460 7e-06 0.00032 //

LOC_Os11g37950 // LOC_Os06g51050 // LOC_Os06g51060 // LOC_Os09g32080 // LOC_Os08g41100 // LOC_Os10g39700 // LOC_Os10g39680 // LOC_Os11g37970

GO:0006032 P chitin catabolic process 8 778 26 24460 7e-06 0.00032 //

LOC_Os11g37950 // LOC_Os06g51050 // LOC_Os06g51060 // LOC_Os09g32080 // LOC_Os08g41100 // LOC_Os10g39700 // LOC_Os10g39680 // LOC_Os11g37970

GO:0006468 P protein amino acid phosphorylation 84 778 1584 24460 6.2e-06 0.00032 //

LOC_Os03g51440 // LOC_Os07g48760 // LOC_Os11g10280 // LOC_Os01g04480 // LOC_Os07g35300 // LOC_Os07g29330 // LOC_Os07g48310 // LOC_Os03g17700 // LOC_Os01g65650 // LOC_Os05g51190 // LOC_Os01g47470 // LOC_Os05g42210 // LOC_Os11g03840 // LOC_Os01g70130 // LOC_Os12g02200 // LOC_Os06g03970 // LOC_Os02g34600 // LOC_Os03g60810 // LOC_Os09g18360 // LOC_Os07g35790 // LOC_Os01g53640 // LOC_Os07g39520 // LOC_Os01g04230 // LOC_Os02g48210 // LOC_Os10g02250 // LOC_Os03g21510 // LOC_Os02g30540 // LOC_Os01g50410 // LOC_Os02g48080 // LOC_Os07g05740 // LOC_Os01g02770 // LOC_Os07g36544 // LOC_Os11g10710 // LOC_Os04g24510 // LOC_Os01g57560 // LOC_Os01g02790 // LOC_Os02g41580 // LOC_Os11g02240 // LOC_Os01g54700 // LOC_Os01g02840 // LOC_Os05g07090 // LOC_Os02g57700 // LOC_Os03g16010 // LOC_Os01g02760 // LOC_Os07g35140 // LOC_Os04g52630 // LOC_Os04g01310 // LOC_Os04g35240 // LOC_Os07g35004 // LOC_Os03g16260 // LOC_Os07g48730 // LOC_Os01g12290 // LOC_Os10g35040 // LOC_Os06g38970 // LOC_Os01g68870 // LOC_Os09g39650 // LOC_Os01g56330 // LOC_Os12g27520 // LOC_Os03g17980 // LOC_Os03g12520 // LOC_Os03g38710 // LOC_Os09g39620 // LOC_Os01g12430 // LOC_Os09g38910 // LOC_Os07g43570 // LOC_Os01g59580 // LOC_Os01g70410 // LOC_Os12g41410 // LOC_Os03g35600 // LOC_Os07g35540 // LOC_Os01g02400 // LOC_Os11g36160 // LOC_Os10g30540 // LOC_Os06g10790 // LOC_Os03g08550 // LOC_Os01g04450 // LOC_Os01g02390 // LOC_Os04g39930 // LOC_Os03g03410 // LOC_Os03g06330 // LOC_Os10g30530 // LOC_Os12g03650 // LOC_Os09g07730 // LOC_Os01g44110

GO:0000272 P polysaccharide catabolic process 9 778 36 24460 7.9e-06 0.00034 //

LOC_Os11g37950 // LOC_Os06g51050 // LOC_Os06g51060 // LOC_Os09g32080 // LOC_Os08g41100 // LOC_Os10g39700 // LOC_Os10g39680 // LOC_Os10g41550 // LOC_Os11g37970

GO:0006022 P aminoglycan metabolic process 8 778 29 24460 1.4e-05 0.00058 //

LOC_Os11g37950 // LOC_Os06g51050 // LOC_Os06g51060 // LOC_Os09g32080 // LOC_Os08g41100 // LOC_Os10g39700 // LOC_Os10g39680 // LOC_Os11g37970

GO:0016052 P carbohydrate catabolic process 16 778 135 24460 1.8e-05 0.00073 //

LOC_Os12g05110 // LOC_Os11g07020 // LOC_Os11g37950 // LOC_Os06g51050 // LOC_Os06g40640 // LOC_Os02g48360 // LOC_Os05g41590 // LOC_Os06g51060 // LOC_Os09g32080 // LOC_Os08g41100 // LOC_Os10g39700 // LOC_Os10g39680 // LOC_Os10g41550 // LOC_Os08g34050 // LOC_Os11g37970 // LOC_Os01g70170

GO:0043687 P post-translational protein modification 90 778 1818 24460 3.5e-05 0.0014 //

LOC_Os01g68870 // LOC_Os02g33680 // LOC_Os05g07090 // LOC_Os03g17700 // LOC_Os05g42210 // LOC_Os11g03840 // LOC_Os01g70130 // LOC_Os02g48210 // LOC_Os07g29330 // LOC_Os06g03970 // LOC_Os01g02790 // LOC_Os03g16010 // LOC_Os01g04230 // LOC_Os03g16260 // LOC_Os09g18360 // LOC_Os06g38970 // LOC_Os04g39930 // LOC_Os10g02250 // LOC_Os03g51440 // LOC_Os02g34600 // LOC_Os11g36160 // LOC_Os01g02760 // LOC_Os03g35600 // LOC_Os03g06330 // LOC_Os10g30530 // LOC_Os12g41410 // LOC_Os01g02400 // LOC_Os03g21510 // LOC_Os01g50410 // LOC_Os01g02770 // LOC_Os07g36544 // LOC_Os01g57560 // LOC_Os02g41580 // LOC_Os04g01310 // LOC_Os03g03410 // LOC_Os10g30540 // LOC_Os01g54700 // LOC_Os09g39650 // LOC_Os12g27520 // LOC_Os03g17980 // LOC_Os06g13870 // LOC_Os07g43570 // LOC_Os06g10790 // LOC_Os01g04450 // LOC_Os07g48760 // LOC_Os07g35300 // LOC_Os07g48310 // LOC_Os07g39520 // LOC_Os04g24510 // LOC_Os11g02240 // LOC_Os02g57700 // LOC_Os02g46500 // LOC_Os07g35140 // LOC_Os04g35240 // LOC_Os07g35004 // LOC_Os03g12520 // LOC_Os01g70410 // LOC_Os07g35540 // LOC_Os01g56330 // LOC_Os12g02200 // LOC_Os01g59580 // LOC_Os05g51190 // LOC_Os02g49950 // LOC_Os03g08550 // LOC_Os01g12430 // LOC_Os03g13010 // LOC_Os03g60810 // LOC_Os01g65650 // LOC_Os01g47470 // LOC_Os07g35790 // LOC_Os09g39620 // LOC_Os02g48080 // LOC_Os07g05740 // LOC_Os11g10710 // LOC_Os01g02840 // LOC_Os01g04480 // LOC_Os04g52630 // LOC_Os01g12290 // LOC_Os07g48730 // LOC_Os01g53640 // LOC_Os03g38710 // LOC_Os09g38910 // LOC_Os05g39930 // LOC_Os11g10280 // LOC_Os10g35040 // LOC_Os01g02390 // LOC_Os02g30540 // LOC_Os12g03650 // LOC_Os09g07730 // LOC_Os01g44110

GO:0016998 P cell wall macromolecule catabolic process 9 778 45 24460 3.7e-05 0.0014 //

LOC_Os10g38040 // LOC_Os11g37950 // LOC_Os06g51050 // LOC_Os06g51060 // LOC_Os09g32080 // LOC_Os08g41100 // LOC_Os10g39700 // LOC_Os10g39680 // LOC_Os11g37970

GO:0016310 P phosphorylation 87 778 1761 24460 5.2e-05 0.0018 //

LOC_Os01g68870 // LOC_Os05g07090 // LOC_Os03g17700 // LOC_Os05g42210 // LOC_Os11g03840 // LOC_Os01g70130 // LOC_Os02g48210 // LOC_Os07g29330 // LOC_Os06g03970 // LOC_Os01g02790 // LOC_Os03g16010 // LOC_Os01g04230 // LOC_Os03g16260 // LOC_Os09g18360 // LOC_Os06g38970 // LOC_Os04g39930 // LOC_Os10g02250 // LOC_Os03g51440 // LOC_Os02g34600 // LOC_Os11g36160 // LOC_Os01g02760 // LOC_Os03g35600 // LOC_Os03g06330 // LOC_Os10g30530 // LOC_Os12g41410 // LOC_Os01g02400 // LOC_Os03g21510 // LOC_Os01g50410 // LOC_Os04g52630 // LOC_Os01g02770 // LOC_Os10g35040 // LOC_Os07g36544 // LOC_Os01g57560 // LOC_Os02g41580 // LOC_Os04g01310 // LOC_Os03g03410 // LOC_Os10g30540 // LOC_Os01g54700 // LOC_Os09g39650 // LOC_Os12g27520 // LOC_Os03g17980 // LOC_Os07g43570 // LOC_Os06g10790 // LOC_Os01g04450 // LOC_Os07g48760 // LOC_Os07g35300 // LOC_Os07g48310 // LOC_Os07g39520 // LOC_Os04g24510 // LOC_Os11g02240 // LOC_Os02g57700 // LOC_Os07g35140 // LOC_Os04g35240 // LOC_Os07g35004 // LOC_Os03g12520 // LOC_Os01g70410 // LOC_Os07g35540 // LOC_Os01g56330 // LOC_Os12g02200 // LOC_Os01g59580 // LOC_Os05g33570 // LOC_Os05g51190 // LOC_Os03g08550 // LOC_Os01g12430 // LOC_Os03g60810 // LOC_Os01g65650 // LOC_Os01g47470 // LOC_Os09g39620 // LOC_Os10g21236 // LOC_Os02g48080 // LOC_Os07g05740 // LOC_Os11g10710 // LOC_Os01g02840 // LOC_Os01g04480 // LOC_Os05g31140 // LOC_Os01g12290 // LOC_Os07g48730 // LOC_Os01g53640 // LOC_Os03g38710 // LOC_Os09g38910 // LOC_Os11g10280 // LOC_Os07g35790 // LOC_Os01g02390 // LOC_Os02g30540 // LOC_Os12g03650 // LOC_Os09g07730 // LOC_Os01g44110

GO:0044036 P cell wall macromolecule metabolic process 9 778 48 24460 5.7e-05 0.002 //

LOC_Os10g38040 // LOC_Os11g37950 // LOC_Os06g51050 // LOC_Os06g51060 // LOC_Os09g32080 // LOC_Os08g41100 // LOC_Os10g39700 // LOC_Os10g39680 // LOC_Os11g37970

GO:0006796 P phosphate metabolic process 89 778 1879 24460 0.00017 0.0055 //

LOC_Os01g68870 // LOC_Os05g07090 // LOC_Os03g17700 // LOC_Os05g42210 // LOC_Os11g03840 // LOC_Os01g70130 // LOC_Os02g48210 // LOC_Os07g29330 // LOC_Os06g03970 // LOC_Os01g02790 // LOC_Os03g16010 // LOC_Os01g04230 // LOC_Os03g16260 // LOC_Os09g18360 // LOC_Os06g38970 // LOC_Os04g39930 // LOC_Os10g02250 // LOC_Os03g51440 // LOC_Os02g34600 // LOC_Os11g36160 // LOC_Os01g02760 // LOC_Os03g35600 // LOC_Os03g06330 // LOC_Os10g30530 // LOC_Os12g41410 // LOC_Os01g02400 // LOC_Os03g21510 // LOC_Os01g50410 // LOC_Os04g52630 // LOC_Os01g02770 // LOC_Os10g35040 // LOC_Os07g36544 // LOC_Os01g57560 // LOC_Os02g41580 // LOC_Os04g01310 // LOC_Os03g03410 // LOC_Os10g30540 // LOC_Os01g54700 // LOC_Os09g39650 // LOC_Os12g27520 // LOC_Os03g17980 // LOC_Os07g43570 // LOC_Os06g10790 // LOC_Os01g04450 // LOC_Os07g48760 // LOC_Os07g35300 // LOC_Os07g48310 // LOC_Os07g39520 // LOC_Os04g24510 // LOC_Os11g02240 // LOC_Os02g57700 // LOC_Os07g35140 // LOC_Os04g35240 // LOC_Os07g35004 // LOC_Os03g12520 // LOC_Os01g70410 // LOC_Os07g35540 // LOC_Os01g56330 // LOC_Os12g02200 // LOC_Os01g59580 // LOC_Os05g33570 // LOC_Os05g41590 // LOC_Os05g51190 // LOC_Os12g23190 // LOC_Os03g08550 // LOC_Os01g12430 // LOC_Os03g60810 // LOC_Os01g65650 // LOC_Os01g47470 // LOC_Os09g39620 // LOC_Os10g21236 // LOC_Os02g48080 // LOC_Os07g05740 // LOC_Os11g10710 // LOC_Os01g02840 // LOC_Os01g04480 // LOC_Os05g31140 // LOC_Os01g12290 // LOC_Os07g48730 // LOC_Os01g53640 // LOC_Os03g38710 // LOC_Os09g38910 // LOC_Os11g10280 // LOC_Os07g35790 // LOC_Os01g02390 // LOC_Os02g30540 // LOC_Os12g03650 // LOC_Os09g07730 // LOC_Os01g44110

GO:0006793 P phosphorus metabolic process 89 778 1879 24460 0.00017 0.0055 //

LOC_Os01g68870 // LOC_Os05g07090 // LOC_Os03g17700 // LOC_Os05g42210 // LOC_Os11g03840 // LOC_Os01g70130 // LOC_Os02g48210 // LOC_Os07g29330 // LOC_Os06g03970 // LOC_Os01g02790 // LOC_Os03g16010 // LOC_Os01g04230 // LOC_Os03g16260 // LOC_Os09g18360 // LOC_Os06g38970 // LOC_Os04g39930 // LOC_Os10g02250 // LOC_Os03g51440 // LOC_Os02g34600 // LOC_Os11g36160 // LOC_Os01g02760 // LOC_Os03g35600 // LOC_Os03g06330 // LOC_Os10g30530 // LOC_Os12g41410 // LOC_Os01g02400 // LOC_Os03g21510 // LOC_Os01g50410 // LOC_Os04g52630 // LOC_Os01g02770 // LOC_Os10g35040 // LOC_Os07g36544 // LOC_Os01g57560 // LOC_Os02g41580 // LOC_Os04g01310 // LOC_Os03g03410 // LOC_Os10g30540 // LOC_Os01g54700 // LOC_Os09g39650 // LOC_Os12g27520 // LOC_Os03g17980 // LOC_Os07g43570 // LOC_Os06g10790 // LOC_Os01g04450 // LOC_Os07g48760 // LOC_Os07g35300 // LOC_Os07g48310 // LOC_Os07g39520 // LOC_Os04g24510 // LOC_Os11g02240 // LOC_Os02g57700 // LOC_Os07g35140 // LOC_Os04g35240 // LOC_Os07g35004 // LOC_Os03g12520 // LOC_Os01g70410 // LOC_Os07g35540 // LOC_Os01g56330 // LOC_Os12g02200 // LOC_Os01g59580 // LOC_Os05g33570 // LOC_Os05g41590 // LOC_Os05g51190 // LOC_Os12g23190 // LOC_Os03g08550 // LOC_Os01g12430 // LOC_Os03g60810 // LOC_Os01g65650 // LOC_Os01g47470 // LOC_Os09g39620 // LOC_Os10g21236 // LOC_Os02g48080 // LOC_Os07g05740 // LOC_Os11g10710 // LOC_Os01g02840 // LOC_Os01g04480 // LOC_Os05g31140 // LOC_Os01g12290 // LOC_Os07g48730 // LOC_Os01g53640 // LOC_Os03g38710 // LOC_Os09g38910 // LOC_Os11g10280 // LOC_Os07g35790 // LOC_Os01g02390 // LOC_Os02g30540 // LOC_Os12g03650 // LOC_Os09g07730 // LOC_Os01g44110

GO:0009056 P catabolic process 29 778 438 24460 0.00032 0.0097 //

LOC_Os01g62500 // LOC_Os08g41100 // LOC_Os08g34050 // LOC_Os01g09220 // LOC_Os01g70170 // LOC_Os02g46650 // LOC_Os01g40070 // LOC_Os10g39680 // LOC_Os05g41180 // LOC_Os10g38040 // LOC_Os06g51060 // LOC_Os01g50980 // LOC_Os01g03730 // LOC_Os09g20440 // LOC_Os12g05110 // LOC_Os08g41630 // LOC_Os09g32080 // LOC_Os10g39700 // LOC_Os10g41550 // LOC_Os02g41650 // LOC_Os11g37970 // LOC_Os11g07020 // LOC_Os11g37950 // LOC_Os06g51050 // LOC_Os06g40640 // LOC_Os02g48360 // LOC_Os05g41590 // LOC_Os08g01940 // LOC_Os01g03740

GO:0009057 P macromolecule catabolic process 23 778 312 24460 0.00032 0.0097 //

LOC_Os01g62500 // LOC_Os08g41100 // LOC_Os08g34050 // LOC_Os01g70170 // LOC_Os02g46650 // LOC_Os10g39680 // LOC_Os06g51060 // LOC_Os01g50980 // LOC_Os01g03730 // LOC_Os05g41180 // LOC_Os12g05110 // LOC_Os08g41630 // LOC_Os09g32080 // LOC_Os10g39700 // LOC_Os10g41550 // LOC_Os11g37970 // LOC_Os11g07020 // LOC_Os11g37950 // LOC_Os06g51050 // LOC_Os06g40640 // LOC_Os02g48360 // LOC_Os05g41590 // LOC_Os01g03740

GO:0006073 P cellular glucan metabolic process 11 778 95 24460 0.00043 0.012 //

LOC_Os09g12660 // LOC_Os06g06560 // LOC_Os03g13570 // LOC_Os06g48200 // LOC_Os03g59340 // LOC_Os01g54620 // LOC_Os06g48160 // LOC_Os07g10770 // LOC_Os02g46910 // LOC_Os05g08370 // LOC_Os04g51460

GO:0044042 P glucan metabolic process 11 778 95 24460 0.00043 0.012 //

LOC_Os09g12660 // LOC_Os06g06560 // LOC_Os03g13570 // LOC_Os06g48200 // LOC_Os03g59340 // LOC_Os01g54620 // LOC_Os06g48160 // LOC_Os07g10770 // LOC_Os02g46910 // LOC_Os05g08370 // LOC_Os04g51460

GO:0044264 P cellular polysaccharide metabolic process 11 778 100 24460 0.00064 0.018 //

LOC_Os09g12660 // LOC_Os06g06560 // LOC_Os03g13570 // LOC_Os06g48200 // LOC_Os03g59340 // LOC_Os01g54620 // LOC_Os06g48160 // LOC_Os07g10770 // LOC_Os02g46910 // LOC_Os05g08370 // LOC_Os04g51460

GO:0006464 P protein modification process 90 778 1997 24460 0.00074 0.02 //

LOC_Os01g68870 // LOC_Os02g33680 // LOC_Os05g07090 // LOC_Os03g17700 // LOC_Os05g42210 // LOC_Os11g03840 // LOC_Os01g70130 // LOC_Os02g48210 // LOC_Os07g29330 // LOC_Os06g03970 // LOC_Os01g02790 // LOC_Os03g16010 // LOC_Os01g04230 // LOC_Os03g16260 // LOC_Os09g18360 // LOC_Os06g38970 // LOC_Os04g39930 // LOC_Os10g02250 // LOC_Os03g51440 // LOC_Os02g34600 // LOC_Os11g36160 // LOC_Os01g02760 // LOC_Os03g35600 // LOC_Os03g06330 // LOC_Os10g30530 // LOC_Os12g41410 // LOC_Os01g02400 // LOC_Os03g21510 // LOC_Os01g50410 // LOC_Os01g02770 // LOC_Os07g36544 // LOC_Os01g57560 // LOC_Os02g41580 // LOC_Os04g01310 // LOC_Os03g03410 // LOC_Os10g30540 // LOC_Os01g54700 // LOC_Os09g39650 // LOC_Os12g27520 // LOC_Os03g17980 // LOC_Os06g13870 // LOC_Os07g43570 // LOC_Os06g10790 // LOC_Os01g04450 // LOC_Os07g48760 // LOC_Os07g35300 // LOC_Os07g48310 // LOC_Os07g39520 // LOC_Os04g24510 // LOC_Os11g02240 // LOC_Os02g57700 // LOC_Os02g46500 // LOC_Os07g35140 // LOC_Os04g35240 // LOC_Os07g35004 // LOC_Os03g12520 // LOC_Os01g70410 // LOC_Os07g35540 // LOC_Os01g56330 // LOC_Os12g02200 // LOC_Os01g59580 // LOC_Os05g51190 // LOC_Os02g49950 // LOC_Os03g08550 // LOC_Os01g12430 // LOC_Os03g13010 // LOC_Os03g60810 // LOC_Os01g65650 // LOC_Os01g47470 // LOC_Os07g35790 // LOC_Os09g39620 // LOC_Os02g48080 // LOC_Os07g05740 // LOC_Os11g10710 // LOC_Os01g02840 // LOC_Os01g04480 // LOC_Os04g52630 // LOC_Os01g12290 // LOC_Os07g48730 // LOC_Os01g53640 // LOC_Os03g38710 // LOC_Os09g38910 // LOC_Os05g39930 // LOC_Os11g10280 // LOC_Os10g35040 // LOC_Os01g02390 // LOC_Os02g30540 // LOC_Os12g03650 // LOC_Os09g07730 // LOC_Os01g44110

GO:0043412 P macromolecule modification 90 778 2046 24460 0.0015 0.039 //

LOC_Os01g68870 // LOC_Os02g33680 // LOC_Os05g07090 // LOC_Os03g17700 // LOC_Os05g42210 // LOC_Os11g03840 // LOC_Os01g70130 // LOC_Os02g48210 // LOC_Os07g29330 // LOC_Os06g03970 // LOC_Os01g02790 // LOC_Os03g16010 // LOC_Os01g04230 // LOC_Os03g16260 // LOC_Os09g18360 // LOC_Os06g38970 // LOC_Os04g39930 // LOC_Os10g02250 // LOC_Os03g51440 // LOC_Os02g34600 // LOC_Os11g36160 // LOC_Os01g02760 // LOC_Os03g35600 // LOC_Os03g06330 // LOC_Os10g30530 // LOC_Os12g41410 // LOC_Os01g02400 // LOC_Os03g21510 // LOC_Os01g50410 // LOC_Os01g02770 // LOC_Os07g36544 // LOC_Os01g57560 // LOC_Os02g41580 // LOC_Os04g01310 // LOC_Os03g03410 // LOC_Os10g30540 // LOC_Os01g54700 // LOC_Os09g39650 // LOC_Os12g27520 // LOC_Os03g17980 // LOC_Os06g13870 // LOC_Os07g43570 // LOC_Os06g10790 // LOC_Os01g04450 // LOC_Os07g48760 // LOC_Os07g35300 // LOC_Os07g48310 // LOC_Os07g39520 // LOC_Os04g24510 // LOC_Os11g02240 // LOC_Os02g57700 // LOC_Os02g46500 // LOC_Os07g35140 // LOC_Os04g35240 // LOC_Os07g35004 // LOC_Os03g12520 // LOC_Os01g70410 // LOC_Os07g35540 // LOC_Os01g56330 // LOC_Os12g02200 // LOC_Os01g59580 // LOC_Os05g51190 // LOC_Os02g49950 // LOC_Os03g08550 // LOC_Os01g12430 // LOC_Os03g13010 // LOC_Os03g60810 // LOC_Os01g65650 // LOC_Os01g47470 // LOC_Os07g35790 // LOC_Os09g39620 // LOC_Os02g48080 // LOC_Os07g05740 // LOC_Os11g10710 // LOC_Os01g02840 // LOC_Os01g04480 // LOC_Os04g52630 // LOC_Os01g12290 // LOC_Os07g48730 // LOC_Os01g53640 // LOC_Os03g38710 // LOC_Os09g38910 // LOC_Os05g39930 // LOC_Os11g10280 // LOC_Os10g35040 // LOC_Os01g02390 // LOC_Os02g30540 // LOC_Os12g03650 // LOC_Os09g07730 // LOC_Os01g44110

GO:0051704 P multi-organism process 11 778 113 24460 0.0016 0.041 //

LOC_Os11g37950 // LOC_Os03g35600 // LOC_Os11g10280 // LOC_Os05g42210 // LOC_Os11g03840 // LOC_Os07g36544 // LOC_Os01g65030 // LOC_Os01g57560 // LOC_Os11g37970 // LOC_Os12g03650 // LOC_Os04g01310

GO:0010467 P gene expression 108 778 2581 24460 0.0025 0.061 //

LOC_Os06g43090 // LOC_Os01g50720 // LOC_Os01g74020 // LOC_Os01g64790 // LOC_Os03g42630 // LOC_Os08g17784 // LOC_Os04g57720 // LOC_Os05g04210 // LOC_Os08g38210 // LOC_Os09g35010 // LOC_Os03g09170 // LOC_Os02g46940 // LOC_Os11g06170 // LOC_Os03g19120 // LOC_Os05g37080 // LOC_Os03g55990 // LOC_Os11g02470 // LOC_Os06g10780 // LOC_Os05g46610 // LOC_Os03g53340 // LOC_Os03g06630 // LOC_Os12g40920 // LOC_Os01g51140 // LOC_Os01g60600 // LOC_Os05g10620 // LOC_Os01g04800 // LOC_Os01g66270 // LOC_Os02g47810 // LOC_Os02g45170 // LOC_Os08g09840 // LOC_Os01g52514 // LOC_Os03g21030 // LOC_Os11g01480 // LOC_Os04g46020 // LOC_Os12g01490 // LOC_Os01g09100 // LOC_Os01g21120 // LOC_Os12g41680 // LOC_Os06g10880 // LOC_Os01g62310 // LOC_Os09g35030 // LOC_Os09g28354 // LOC_Os09g28210 // LOC_Os02g43790 // LOC_Os03g03164 // LOC_Os01g09590 // LOC_Os12g02400 // LOC_Os05g49100 // LOC_Os06g03670 // LOC_Os09g25060 // LOC_Os04g16728 // LOC_Os06g41060 // LOC_Os11g29870 // LOC_Os08g43334 // LOC_Os10g42490 // LOC_Os09g35020 // LOC_Os04g32620 // LOC_Os03g53800 // LOC_Os04g23550 // LOC_Os01g55340 // LOC_Os07g34620 // LOC_Os08g41320 // LOC_Os02g09480 // LOC_Os11g03300 // LOC_Os01g01840 // LOC_Os02g36880 // LOC_Os07g02060 // LOC_Os01g50940 // LOC_Os01g06640 // LOC_Os04g40630 // LOC_Os02g08440 // LOC_Os02g42850 // LOC_Os01g58760 // LOC_Os01g60020 // LOC_Os11g02520 // LOC_Os05g37520 // LOC_Os02g58210 // LOC_Os08g43210 // LOC_Os02g46500 // LOC_Os01g12440 // LOC_Os02g09100 // LOC_Os01g68020 // LOC_Os01g62950 // LOC_Os01g18584 // LOC_Os04g40140 // LOC_Os02g04640 // LOC_Os08g02300 // LOC_Os02g45450 // LOC_Os04g41570 // LOC_Os02g38130 // LOC_Os10g42230 // LOC_Os09g28440 // LOC_Os03g53150 // LOC_Os03g55590 // LOC_Os02g45420 // LOC_Os11g45740 // LOC_Os09g36910 // LOC_Os05g23260 // LOC_Os08g34360 // LOC_Os01g56690 // LOC_Os06g49040 // LOC_Os07g43530 // LOC_Os03g20090 // LOC_Os04g58190 // LOC_Os05g08570 // LOC_Os03g59670 // LOC_Os01g06740 // LOC_Os08g09900

GO:0071554 P cell wall organization or biogenesis 12 778 146 24460 0.0037 0.089 //

LOC_Os10g38040 // LOC_Os11g37950 // LOC_Os06g51050 // LOC_Os09g26360 // LOC_Os06g51060 // LOC_Os09g32080 // LOC_Os08g41100 // LOC_Os10g39700 // LOC_Os10g39680 // LOC_Os04g38560 // LOC_Os11g37970 // LOC_Os02g17534

GO:0006355 P regulation of transcription, DNA-dependent 40 778 806 24460 0.0052 0.12 //

LOC_Os04g46020 // LOC_Os08g34360 // LOC_Os01g64790 // LOC_Os02g45450 // LOC_Os01g21120 // LOC_Os06g10880 // LOC_Os01g62310 // LOC_Os09g35030 // LOC_Os03g53340 // LOC_Os09g35010 // LOC_Os09g28354 // LOC_Os03g09170 // LOC_Os09g36910 // LOC_Os02g43790 // LOC_Os01g58760 // LOC_Os10g42230 // LOC_Os09g28440 // LOC_Os03g55990 // LOC_Os06g03670 // LOC_Os06g10780 // LOC_Os02g58210 // LOC_Os02g45420 // LOC_Os08g43210 // LOC_Os02g46500 // LOC_Os03g06630 // LOC_Os08g43334 // LOC_Os12g40920 // LOC_Os10g42490 // LOC_Os01g04800 // LOC_Os01g66270 // LOC_Os09g35020 // LOC_Os04g40630 // LOC_Os11g06170 // LOC_Os03g03164 // LOC_Os04g32620 // LOC_Os01g68020 // LOC_Os01g52514 // LOC_Os01g12440 // LOC_Os01g62950 // LOC_Os04g57720

GO:0051252 P regulation of RNA metabolic process 40 778 809 24460 0.0055 0.13 //

LOC_Os04g46020 // LOC_Os08g34360 // LOC_Os01g64790 // LOC_Os02g45450 // LOC_Os01g21120 // LOC_Os06g10880 // LOC_Os01g62310 // LOC_Os09g35030 // LOC_Os03g53340 // LOC_Os09g35010 // LOC_Os09g28354 // LOC_Os03g09170 // LOC_Os09g36910 // LOC_Os02g43790 // LOC_Os01g58760 // LOC_Os10g42230 // LOC_Os09g28440 // LOC_Os03g55990 // LOC_Os06g03670 // LOC_Os06g10780 // LOC_Os02g58210 // LOC_Os02g45420 // LOC_Os08g43210 // LOC_Os02g46500 // LOC_Os03g06630 // LOC_Os08g43334 // LOC_Os12g40920 // LOC_Os10g42490 // LOC_Os01g04800 // LOC_Os01g66270 // LOC_Os09g35020 // LOC_Os04g40630 // LOC_Os11g06170 // LOC_Os03g03164 // LOC_Os04g32620 // LOC_Os01g68020 // LOC_Os01g52514 // LOC_Os01g12440 // LOC_Os01g62950 // LOC_Os04g57720

GO:0009875 P pollen-pistil interaction 9 778 99 24460 0.0062 0.13 //

LOC_Os03g35600 // LOC_Os11g10280 // LOC_Os05g42210 // LOC_Os11g03840 // LOC_Os07g36544 // LOC_Os01g65030 // LOC_Os01g57560 // LOC_Os12g03650 // LOC_Os04g01310

GO:0008037 P cell recognition 9 778 99 24460 0.0062 0.13 //

LOC_Os03g35600 // LOC_Os11g10280 // LOC_Os05g42210 // LOC_Os11g03840 // LOC_Os07g36544 // LOC_Os01g65030 // LOC_Os01g57560 // LOC_Os12g03650 // LOC_Os04g01310

GO:0048544 P recognition of pollen 9 778 99 24460 0.0062 0.13 //

LOC_Os03g35600 // LOC_Os11g10280 // LOC_Os05g42210 // LOC_Os11g03840 // LOC_Os07g36544 // LOC_Os01g65030 // LOC_Os01g57560 // LOC_Os12g03650 // LOC_Os04g01310

GO:0009856 P pollination 9 778 99 24460 0.0062 0.13 //

LOC_Os03g35600 // LOC_Os11g10280 // LOC_Os05g42210 // LOC_Os11g03840 // LOC_Os07g36544 // LOC_Os01g65030 // LOC_Os01g57560 // LOC_Os12g03650 // LOC_Os04g01310

GO:0044262 P cellular carbohydrate metabolic process 22 778 379 24460 0.007 0.14 //

LOC_Os08g34050 // LOC_Os01g70170 // LOC_Os03g59340 // LOC_Os02g37590 // LOC_Os09g12660 // LOC_Os04g40874 // LOC_Os06g48200 // LOC_Os06g48160 // LOC_Os05g08370 // LOC_Os12g05110 // LOC_Os06g06560 // LOC_Os03g13570 // LOC_Os01g54620 // LOC_Os02g46910 // LOC_Os04g51460 // LOC_Os11g07020 // LOC_Os06g40640 // LOC_Os02g48360 // LOC_Os05g41590 // LOC_Os07g10770 // LOC_Os05g44210 // LOC_Os04g52730

GO:0009116 P nucleoside metabolic process 6 778 50 24460 0.0074 0.15 //

LOC_Os02g50350 // LOC_Os01g52530 // LOC_Os05g13970 // LOC_Os01g39830 // LOC_Os06g10520 // LOC_Os01g65260

GO:0022414 P reproductive process 9 778 106 24460 0.0093 0.18 //

LOC_Os03g35600 // LOC_Os11g10280 // LOC_Os05g42210 // LOC_Os11g03840 // LOC_Os07g36544 // LOC_Os01g65030 // LOC_Os01g57560 // LOC_Os12g03650 // LOC_Os04g01310

GO:0019725 P cellular homeostasis 10 778 129 24460 0.011 0.21 //

LOC_Os02g51370 // LOC_Os05g48930 // LOC_Os03g29240 // LOC_Os07g29410 // LOC_Os11g09280 // LOC_Os06g06790 // LOC_Os02g06480 // LOC_Os03g21000 // LOC_Os12g01530 // LOC_Os11g01530

GO:0016567 P protein ubiquitination 8 778 93 24460 0.013 0.24 //

LOC_Os03g13010 // LOC_Os02g57700 // LOC_Os02g46500 // LOC_Os02g33680 // LOC_Os06g13870 // LOC_Os05g39930 // LOC_Os02g49950 // LOC_Os09g39620

GO:0006351 P transcription, DNA-dependent 40 778 855 24460 0.013 0.24 //

LOC_Os04g46020 // LOC_Os08g34360 // LOC_Os01g64790 // LOC_Os02g45450 // LOC_Os01g21120 // LOC_Os06g10880 // LOC_Os01g62310 // LOC_Os09g35030 // LOC_Os03g53340 // LOC_Os09g35010 // LOC_Os09g28354 // LOC_Os03g09170 // LOC_Os09g36910 // LOC_Os02g43790 // LOC_Os01g58760 // LOC_Os10g42230 // LOC_Os09g28440 // LOC_Os03g55990 // LOC_Os06g03670 // LOC_Os06g10780 // LOC_Os02g58210 // LOC_Os02g45420 // LOC_Os08g43210 // LOC_Os02g46500 // LOC_Os03g06630 // LOC_Os08g43334 // LOC_Os12g40920 // LOC_Os10g42490 // LOC_Os01g04800 // LOC_Os01g66270 // LOC_Os09g35020 // LOC_Os04g40630 // LOC_Os11g06170 // LOC_Os03g03164 // LOC_Os04g32620 // LOC_Os01g68020 // LOC_Os01g52514 // LOC_Os01g12440 // LOC_Os01g62950 // LOC_Os04g57720

GO:0032774 P RNA biosynthetic process 40 778 857 24460 0.013 0.24 //

LOC_Os04g46020 // LOC_Os08g34360 // LOC_Os01g64790 // LOC_Os02g45450 // LOC_Os01g21120 // LOC_Os06g10880 // LOC_Os01g62310 // LOC_Os09g35030 // LOC_Os03g53340 // LOC_Os09g35010 // LOC_Os09g28354 // LOC_Os03g09170 // LOC_Os09g36910 // LOC_Os02g43790 // LOC_Os01g58760 // LOC_Os10g42230 // LOC_Os09g28440 // LOC_Os03g55990 // LOC_Os06g03670 // LOC_Os06g10780 // LOC_Os02g58210 // LOC_Os02g45420 // LOC_Os08g43210 // LOC_Os02g46500 // LOC_Os03g06630 // LOC_Os08g43334 // LOC_Os12g40920 // LOC_Os10g42490 // LOC_Os01g04800 // LOC_Os01g66270 // LOC_Os09g35020 // LOC_Os04g40630 // LOC_Os11g06170 // LOC_Os03g03164 // LOC_Os04g32620 // LOC_Os01g68020 // LOC_Os01g52514 // LOC_Os01g12440 // LOC_Os01g62950 // LOC_Os04g57720

GO:0070647 P protein modification by small protein conjugation or removal 8 778 94 24460 0.014 0.24 //

LOC_Os03g13010 // LOC_Os02g57700 // LOC_Os02g46500 // LOC_Os02g33680 // LOC_Os06g13870 // LOC_Os05g39930 // LOC_Os02g49950 // LOC_Os09g39620

GO:0032446 P protein modification by small protein conjugation 8 778 94 24460 0.014 0.24 //

LOC_Os03g13010 // LOC_Os02g57700 // LOC_Os02g46500 // LOC_Os02g33680 // LOC_Os06g13870 // LOC_Os05g39930 // LOC_Os02g49950 // LOC_Os09g39620

GO:0032501 P multicellular organismal process 11 778 155 24460 0.014 0.24 //

LOC_Os03g35600 // LOC_Os12g03360 // LOC_Os11g10280 // LOC_Os04g51150 // LOC_Os05g42210 // LOC_Os11g03840 // LOC_Os07g36544 // LOC_Os01g65030 // LOC_Os01g57560 // LOC_Os12g03650 // LOC_Os04g01310

GO:0042592 P homeostatic process 10 778 136 24460 0.015 0.26 //

LOC_Os02g51370 // LOC_Os05g48930 // LOC_Os03g29240 // LOC_Os07g29410 // LOC_Os11g09280 // LOC_Os06g06790 // LOC_Os02g06480 // LOC_Os03g21000 // LOC_Os12g01530 // LOC_Os11g01530

GO:0009628 P response to abiotic stimulus 6 778 60 24460 0.016 0.27 //

LOC_Os04g35240 // LOC_Os12g02530 // LOC_Os11g02610 // LOC_Os02g34600 // LOC_Os12g02540 // LOC_Os03g10880

GO:0030001 P metal ion transport 14 778 225 24460 0.017 0.27 //

LOC_Os03g02070 // LOC_Os07g12900 // LOC_Os04g39010 // LOC_Os02g06480 // LOC_Os02g37330 // LOC_Os03g54100 // LOC_Os01g70240 // LOC_Os07g01214 // LOC_Os04g57200 // LOC_Os04g32800 // LOC_Os01g70710 // LOC_Os10g30450 // LOC_Os12g01530 // LOC_Os11g01530

GO:0009308 P amine metabolic process 22 778 414 24460 0.017 0.27 //

LOC_Os08g41100 // LOC_Os03g12890 // LOC_Os03g63330 // LOC_Os10g39680 // LOC_Os02g09100 // LOC_Os04g20164 // LOC_Os02g39790 // LOC_Os12g42980 // LOC_Os03g58300 // LOC_Os06g51060 // LOC_Os01g51410 // LOC_Os04g57410 // LOC_Os07g46460 // LOC_Os09g32080 // LOC_Os10g37340 // LOC_Os03g53800 // LOC_Os10g39700 // LOC_Os11g37970 // LOC_Os11g37950 // LOC_Os06g51050 // LOC_Os02g41650 // LOC_Os04g51400

GO:0009250 P glucan biosynthetic process 6 778 65 24460 0.022 0.34 // LOC_Os09g12660 // LOC_Os06g06560 // LOC_Os03g59340 // LOC_Os01g54620 // LOC_Os07g10770 // LOC_Os05g08370

GO:0043623 P cellular protein complex assembly 6 778 65 24460 0.022 0.34 // LOC_Os02g55970 // LOC_Os01g59150 // LOC_Os02g06480 // LOC_Os03g44420 // LOC_Os02g07060 // LOC_Os04g56970

GO:0000003 P reproduction 9 778 125 24460 0.023 0.35 //

LOC_Os03g35600 // LOC_Os11g10280 // LOC_Os05g42210 // LOC_Os11g03840 // LOC_Os07g36544 // LOC_Os01g65030 // LOC_Os01g57560 // LOC_Os12g03650 // LOC_Os04g01310

GO:0033692 P cellular polysaccharide biosynthetic process 6 778 70 24460 0.03 0.45 //

LOC_Os09g12660 // LOC_Os06g06560 // LOC_Os03g59340 // LOC_Os01g54620 // LOC_Os07g10770 // LOC_Os05g08370

GO:0006006 P glucose metabolic process 8 778 112 24460 0.032 0.48 //

LOC_Os12g05110 // LOC_Os11g07020 // LOC_Os09g12660 // LOC_Os06g40640 // LOC_Os04g40874 // LOC_Os02g48360 // LOC_Os01g70170 // LOC_Os08g34050

GO:0019318 P hexose metabolic process 9 778 134 24460 0.033 0.49 //

LOC_Os12g05110 // LOC_Os11g07020 // LOC_Os09g12660 // LOC_Os06g40640 // LOC_Os04g40874 // LOC_Os02g48360 // LOC_Os01g70170 // LOC_Os08g34050 // LOC_Os04g52730

GO:0000271 P polysaccharide biosynthetic process 6 778 73 24460 0.035 0.51 //

LOC_Os09g12660 // LOC_Os06g06560 // LOC_Os03g59340 // LOC_Os01g54620 // LOC_Os07g10770 // LOC_Os05g08370

GO:0044275 P cellular carbohydrate catabolic process 7 778 98 24460 0.044 0.61 // LOC_Os12g05110 // LOC_Os11g07020 // LOC_Os08g34050 // LOC_Os02g48360 // LOC_Os05g41590 // LOC_Os01g70170 // LOC_Os06g40640

GO:0046164 P alcohol catabolic process 7 778 98 24460 0.044 0.61 //

LOC_Os12g05110 // LOC_Os11g07020 // LOC_Os08g34050 // LOC_Os02g48360 // LOC_Os05g41590 // LOC_Os01g70170 // LOC_Os06g40640

GO:0015979 P photosynthesis 7 778 100 24460 0.048 0.66 //

LOC_Os12g23200 // LOC_Os01g41710 // LOC_Os07g25430 // LOC_Os01g31690 // LOC_Os07g05480 // LOC_Os03g39610 // LOC_Os07g37550

GO:0004553 F hydrolase activity, hydrolyzing O-glycosyl compounds 46 778 454 24460 4.8e-11 3.4e-08 //

LOC_Os08g41100 // LOC_Os01g71474 // LOC_Os11g43750 // LOC_Os09g29600 // LOC_Os07g46280 // LOC_Os01g39830 // LOC_Os10g39680 // LOC_Os09g31270 // LOC_Os04g39900 // LOC_Os06g25010 // LOC_Os03g12140 // LOC_Os01g71810 // LOC_Os04g51460 // LOC_Os06g51060 // LOC_Os01g64170 // LOC_Os06g48200 // LOC_Os06g48160 // LOC_Os04g41970 // LOC_Os04g39880 // LOC_Os03g49610 // LOC_Os10g35070 // LOC_Os02g05744 // LOC_Os03g13570 // LOC_Os05g32710 // LOC_Os05g15770 // LOC_Os06g46284 // LOC_Os09g32080 // LOC_Os03g25790 // LOC_Os06g51050 // LOC_Os10g39700 // LOC_Os05g31140 // LOC_Os10g41550 // LOC_Os09g36350 // LOC_Os02g46910 // LOC_Os11g37970 // LOC_Os01g03950 // LOC_Os05g15510 // LOC_Os11g37950 // LOC_Os03g53800 // LOC_Os09g23084 // LOC_Os01g43160 // LOC_Os08g01940 // LOC_Os02g50490 // LOC_Os09g33690 // LOC_Os06g46940 // LOC_Os03g49600

GO:0016798 F hydrolase activity, acting on glycosyl bonds 48 778 503 24460 1.2e-10 4.2e-08 //

LOC_Os08g41100 // LOC_Os01g71474 // LOC_Os11g43750 // LOC_Os09g29600 // LOC_Os07g46280 // LOC_Os01g39830 // LOC_Os10g39680 // LOC_Os09g31270 // LOC_Os04g39900 // LOC_Os06g25010 // LOC_Os03g12140 // LOC_Os01g71810 // LOC_Os04g51460 // LOC_Os06g51060 // LOC_Os01g64170 // LOC_Os06g48200 // LOC_Os06g48160 // LOC_Os04g41970 // LOC_Os04g39880 // LOC_Os03g49610 // LOC_Os10g35070 // LOC_Os02g05744 // LOC_Os03g13570 // LOC_Os05g32710 // LOC_Os05g15770 // LOC_Os06g46284 // LOC_Os09g32080 // LOC_Os03g25790 // LOC_Os06g51050 // LOC_Os10g39700 // LOC_Os05g31140 // LOC_Os10g41550 // LOC_Os09g36350 // LOC_Os02g46910 // LOC_Os11g37970 // LOC_Os01g03950 // LOC_Os05g15510 // LOC_Os11g37950 // LOC_Os03g53800 // LOC_Os09g23084 // LOC_Os01g43160 // LOC_Os08g01940 // LOC_Os02g50490 // LOC_Os12g38920 // LOC_Os09g33690 // LOC_Os06g46940 // LOC_Os03g49600 // LOC_Os01g06740

GO:0016757 F transferase activity, transferring glycosyl groups 46 778 547 24460 1.2e-08 2.7e-06 //

LOC_Os04g12950 // LOC_Os01g53330 // LOC_Os07g31960 // LOC_Os03g18890 // LOC_Os09g34230 // LOC_Os02g17534 // LOC_Os03g59340 // LOC_Os06g28124 // LOC_Os01g53430 // LOC_Os04g58040 // LOC_Os04g25440 // LOC_Os07g32060 // LOC_Os07g32630 // LOC_Os06g49810 // LOC_Os01g65780 // LOC_Os04g46990 // LOC_Os05g45090 // LOC_Os03g55090 // LOC_Os02g36840 // LOC_Os02g14630 // LOC_Os02g49140 // LOC_Os06g48200 // LOC_Os07g48370 // LOC_Os06g48160 // LOC_Os03g17850 // LOC_Os01g65260 // LOC_Os05g08370 // LOC_Os04g12960 // LOC_Os06g06560 // LOC_Os03g13570 // LOC_Os03g59350 // LOC_Os04g46970 // LOC_Os01g54620 // LOC_Os01g08440 // LOC_Os06g12280 // LOC_Os06g17090 // LOC_Os01g52710 // LOC_Os01g31370 // LOC_Os05g12450 // LOC_Os02g46910 // LOC_Os04g51460 // LOC_Os04g20400 // LOC_Os04g47720 // LOC_Os07g10770 // LOC_Os05g44210 // LOC_Os06g11720

GO:0030528 F transcription regulator activity 65 778 1037 24460 4.7e-07 6.1e-05 //

LOC_Os04g46020 // LOC_Os08g34360 // LOC_Os07g02060 // LOC_Os01g50940 // LOC_Os06g28550 // LOC_Os09g35020 // LOC_Os01g06640 // LOC_Os02g45450 // LOC_Os01g21120 // LOC_Os06g10880 // LOC_Os01g62310 // LOC_Os02g08440 // LOC_Os09g35030 // LOC_Os08g43210 // LOC_Os08g38210 // LOC_Os09g35010 // LOC_Os09g28354 // LOC_Os03g09170 // LOC_Os03g55990 // LOC_Os09g28210 // LOC_Os02g43790 // LOC_Os01g58760 // LOC_Os01g68020 // LOC_Os09g28440 // LOC_Os01g09100 // LOC_Os12g02400 // LOC_Os04g40630 // LOC_Os05g49100 // LOC_Os06g03670 // LOC_Os11g02470 // LOC_Os11g02520 // LOC_Os06g10780 // LOC_Os09g25060 // LOC_Os02g45420 // LOC_Os11g29870 // LOC_Os01g64790 // LOC_Os03g53340 // LOC_Os02g46500 // LOC_Os03g06630 // LOC_Os08g43334 // LOC_Os12g40920 // LOC_Os09g36910 // LOC_Os01g51140 // LOC_Os01g60600 // LOC_Os01g04800 // LOC_Os01g66270 // LOC_Os01g56690 // LOC_Os07g43530 // LOC_Os04g41570 // LOC_Os11g06170 // LOC_Os02g45170 // LOC_Os03g03164 // LOC_Os03g22780 // LOC_Os04g32620 // LOC_Os06g41060 // LOC_Os04g23550 // LOC_Os08g09840 // LOC_Os10g42490 // LOC_Os01g12440 // LOC_Os01g01840 // LOC_Os08g41320 // LOC_Os01g18584 // LOC_Os03g59670 // LOC_Os08g09900 // LOC_Os04g57720

GO:0016758 F transferase activity, transferring hexosyl groups 37 778 450 24460 5.3e-07 6.1e-05 //

LOC_Os04g12950 // LOC_Os01g53330 // LOC_Os07g31960 // LOC_Os09g34230 // LOC_Os02g17534 // LOC_Os03g59340 // LOC_Os01g53430 // LOC_Os04g58040 // LOC_Os04g25440 // LOC_Os02g49140 // LOC_Os07g32630 // LOC_Os04g46990 // LOC_Os05g45090 // LOC_Os03g55090 // LOC_Os02g36840 // LOC_Os02g14630 // LOC_Os07g32060 // LOC_Os06g48200 // LOC_Os06g48160 // LOC_Os03g17850 // LOC_Os05g08370 // LOC_Os04g12960 // LOC_Os06g06560 // LOC_Os03g13570 // LOC_Os03g59350 // LOC_Os04g46970 // LOC_Os01g54620 // LOC_Os01g08440 // LOC_Os06g17090 // LOC_Os05g12450 // LOC_Os02g46910 // LOC_Os04g51460 // LOC_Os04g20400 // LOC_Os04g47720 // LOC_Os07g10770 // LOC_Os05g44210 // LOC_Os06g11720

GO:0004674 F protein serine/threonine kinase activity 82 778 1424 24460 4e-07 6.1e-05 //

LOC_Os03g51440 // LOC_Os07g48760 // LOC_Os11g10280 // LOC_Os01g04480 // LOC_Os07g35300 // LOC_Os07g29330 // LOC_Os07g48310 // LOC_Os03g17700 // LOC_Os01g65650 // LOC_Os05g51190 // LOC_Os01g47470 // LOC_Os05g42210 // LOC_Os11g03840 // LOC_Os01g70130 // LOC_Os12g02200 // LOC_Os06g03970 // LOC_Os02g34600 // LOC_Os03g60810 // LOC_Os09g18360 // LOC_Os07g35790 // LOC_Os01g53640 // LOC_Os07g39520 // LOC_Os01g04230 // LOC_Os02g48210 // LOC_Os10g02250 // LOC_Os03g21510 // LOC_Os02g30540 // LOC_Os01g50410 // LOC_Os02g48080 // LOC_Os07g05740 // LOC_Os01g02770 // LOC_Os07g36544 // LOC_Os11g10710 // LOC_Os04g24510 // LOC_Os01g57560 // LOC_Os01g02790 // LOC_Os02g41580 // LOC_Os11g02240 // LOC_Os01g54700 // LOC_Os01g02840 // LOC_Os02g57700 // LOC_Os03g16010 // LOC_Os01g02760 // LOC_Os07g35140 // LOC_Os04g52630 // LOC_Os04g01310 // LOC_Os04g35240 // LOC_Os07g35004 // LOC_Os03g16260 // LOC_Os07g48730 // LOC_Os01g12290 // LOC_Os10g35040 // LOC_Os06g38970 // LOC_Os01g68870 // LOC_Os09g39650 // LOC_Os01g56330 // LOC_Os12g27520 // LOC_Os03g17980 // LOC_Os03g12520 // LOC_Os03g38710 // LOC_Os09g39620 // LOC_Os01g12430 // LOC_Os09g38910 // LOC_Os07g43570 // LOC_Os01g59580 // LOC_Os01g70410 // LOC_Os03g35600 // LOC_Os07g35540 // LOC_Os01g02400 // LOC_Os11g36160 // LOC_Os10g30540 // LOC_Os06g10790 // LOC_Os03g08550 // LOC_Os01g04450 // LOC_Os01g02390 // LOC_Os04g39930 // LOC_Os03g03410 // LOC_Os03g06330 // LOC_Os10g30530 // LOC_Os12g03650 // LOC_Os09g07730 // LOC_Os01g44110

GO:0004713 F protein tyrosine kinase activity 81 778 1426 24460 7.8e-07 7.8e-05 //

LOC_Os03g51440 // LOC_Os07g48760 // LOC_Os11g10280 // LOC_Os01g04480 // LOC_Os07g35300 // LOC_Os07g29330 // LOC_Os07g48310 // LOC_Os03g17700 // LOC_Os01g65650 // LOC_Os05g51190 // LOC_Os01g47470 // LOC_Os05g42210 // LOC_Os11g03840 // LOC_Os01g70130 // LOC_Os12g02200 // LOC_Os06g03970 // LOC_Os02g34600 // LOC_Os03g60810 // LOC_Os09g18360 // LOC_Os07g35790 // LOC_Os01g53640 // LOC_Os07g39520 // LOC_Os01g04230 // LOC_Os02g48210 // LOC_Os10g02250 // LOC_Os03g21510 // LOC_Os02g30540 // LOC_Os01g50410 // LOC_Os02g48080 // LOC_Os07g05740 // LOC_Os01g02770 // LOC_Os07g36544 // LOC_Os11g10710 // LOC_Os01g57560 // LOC_Os01g02790 // LOC_Os02g41580 // LOC_Os11g02240 // LOC_Os01g54700 // LOC_Os01g02840 // LOC_Os02g57700 // LOC_Os03g16010 // LOC_Os01g02760 // LOC_Os07g35140 // LOC_Os04g52630 // LOC_Os04g01310 // LOC_Os04g35240 // LOC_Os07g35004 // LOC_Os03g16260 // LOC_Os07g48730 // LOC_Os01g12290 // LOC_Os10g35040 // LOC_Os06g38970 // LOC_Os01g68870 // LOC_Os09g39650 // LOC_Os01g56330 // LOC_Os12g27520 // LOC_Os03g17980 // LOC_Os03g12520 // LOC_Os03g38710 // LOC_Os09g39620 // LOC_Os01g12430 // LOC_Os09g38910 // LOC_Os07g43570 // LOC_Os01g59580 // LOC_Os01g70410 // LOC_Os03g35600 // LOC_Os07g35540 // LOC_Os01g02400 // LOC_Os11g36160 // LOC_Os10g30540 // LOC_Os06g10790 // LOC_Os03g08550 // LOC_Os01g04450 // LOC_Os01g02390 // LOC_Os04g39930 // LOC_Os03g03410 // LOC_Os03g06330 // LOC_Os10g30530 // LOC_Os12g03650 // LOC_Os09g07730 // LOC_Os01g44110

GO:0016301 F kinase activity 94 778 1756 24460 1.1e-06 9.9e-05 //

LOC_Os01g68870 // LOC_Os03g16010 // LOC_Os03g17700 // LOC_Os05g42210 // LOC_Os11g03840 // LOC_Os01g70130 // LOC_Os02g48210 // LOC_Os07g29330 // LOC_Os02g47020 // LOC_Os06g03970 // LOC_Os01g02790 // LOC_Os04g57410 // LOC_Os01g04230 // LOC_Os03g16260 // LOC_Os09g18360 // LOC_Os06g38970 // LOC_Os04g39930 // LOC_Os10g02250 // LOC_Os03g51440 // LOC_Os02g34600 // LOC_Os11g36160 // LOC_Os01g02760 // LOC_Os03g35600 // LOC_Os03g06330 // LOC_Os10g30530 // LOC_Os08g34050 // LOC_Os12g41410 // LOC_Os01g02400 // LOC_Os04g56580 // LOC_Os03g21510 // LOC_Os01g50410 // LOC_Os05g31140 // LOC_Os01g02770 // LOC_Os07g36544 // LOC_Os01g57560 // LOC_Os02g41580 // LOC_Os04g01310 // LOC_Os12g05110 // LOC_Os03g03410 // LOC_Os10g30540 // LOC_Os01g54700 // LOC_Os09g39650 // LOC_Os12g27520 // LOC_Os03g17980 // LOC_Os07g43570 // LOC_Os02g48360 // LOC_Os06g10790 // LOC_Os01g04450 // LOC_Os07g48760 // LOC_Os07g35300 // LOC_Os07g48310 // LOC_Os07g39520 // LOC_Os05g07090 // LOC_Os04g24510 // LOC_Os11g02240 // LOC_Os02g57700 // LOC_Os07g35140 // LOC_Os04g35240 // LOC_Os07g35004 // LOC_Os03g12520 // LOC_Os01g70410 // LOC_Os07g35540 // LOC_Os01g56330 // LOC_Os12g02200 // LOC_Os01g59580 // LOC_Os05g33570 // LOC_Os05g51190 // LOC_Os03g08550 // LOC_Os01g12430 // LOC_Os03g60810 // LOC_Os01g65650 // LOC_Os03g63330 // LOC_Os01g47470 // LOC_Os07g35790 // LOC_Os09g39620 // LOC_Os02g48080 // LOC_Os07g05740 // LOC_Os11g10710 // LOC_Os01g02840 // LOC_Os01g04480 // LOC_Os04g52630 // LOC_Os01g12290 // LOC_Os07g48730 // LOC_Os06g10520 // LOC_Os01g53640 // LOC_Os03g38710 // LOC_Os09g38910 // LOC_Os11g10280 // LOC_Os10g35040 // LOC_Os01g02390 // LOC_Os02g30540 // LOC_Os12g03650 // LOC_Os09g07730 // LOC_Os01g44110

GO:0009055 F electron carrier activity 57 778 919 24460 3.3e-06 0.00025 //

LOC_Os12g04480 // LOC_Os11g05380 // LOC_Os07g44130 // LOC_Os04g59190 // LOC_Os07g44140 // LOC_Os01g25820 // LOC_Os10g30410 // LOC_Os03g57120 // LOC_Os03g44740 // LOC_Os05g41440 // LOC_Os01g57730 // LOC_Os07g48050 // LOC_Os01g22336 // LOC_Os01g16450 // LOC_Os04g57550 // LOC_Os07g38290 // LOC_Os01g53294 // LOC_Os08g35210 // LOC_Os09g26660 // LOC_Os06g46910 // LOC_Os08g37660 // LOC_Os05g48930 // LOC_Os02g56930 // LOC_Os03g55120 // LOC_Os07g42440 // LOC_Os10g38110 // LOC_Os08g17160 // LOC_Os03g63310 // LOC_Os10g08474 // LOC_Os01g43851 // LOC_Os01g43700 // LOC_Os10g36960 // LOC_Os02g47470 // LOC_Os05g07090 // LOC_Os02g51370 // LOC_Os07g33600 // LOC_Os02g40840 // LOC_Os07g23570 // LOC_Os08g01490 // LOC_Os06g28050 // LOC_Os04g59200 // LOC_Os09g20440 // LOC_Os01g59000 // LOC_Os04g33630 // LOC_Os04g40460 // LOC_Os07g02440 // LOC_Os08g39840 // LOC_Os07g02200 // LOC_Os02g10120 // LOC_Os03g12660 // LOC_Os02g12890 // LOC_Os02g43660 // LOC_Os07g18120 // LOC_Os07g47990 // LOC_Os02g45710 // LOC_Os02g57810 // LOC_Os04g36720

GO:0016773 F phosphotransferase activity, alcohol group as acceptor 91 778 1738 24460 4e-06 0.00026 //

LOC_Os01g68870 // LOC_Os03g16010 // LOC_Os03g17700 // LOC_Os05g42210 // LOC_Os11g03840 // LOC_Os01g70130 // LOC_Os02g48210 // LOC_Os07g29330 // LOC_Os02g47020 // LOC_Os06g03970 // LOC_Os01g02790 // LOC_Os04g57410 // LOC_Os01g04230 // LOC_Os03g16260 // LOC_Os09g18360 // LOC_Os06g38970 // LOC_Os04g39930 // LOC_Os10g02250 // LOC_Os03g51440 // LOC_Os02g34600 // LOC_Os11g36160 // LOC_Os01g02760 // LOC_Os03g35600 // LOC_Os03g06330 // LOC_Os10g30530 // LOC_Os08g34050 // LOC_Os12g41410 // LOC_Os01g02400 // LOC_Os04g56580 // LOC_Os03g21510 // LOC_Os01g50410 // LOC_Os01g02770 // LOC_Os07g36544 // LOC_Os01g57560 // LOC_Os02g41580 // LOC_Os04g01310 // LOC_Os12g05110 // LOC_Os03g03410 // LOC_Os10g30540 // LOC_Os01g54700 // LOC_Os09g39650 // LOC_Os12g27520 // LOC_Os03g17980 // LOC_Os07g43570 // LOC_Os02g48360 // LOC_Os06g10790 // LOC_Os01g04450 // LOC_Os07g48760 // LOC_Os07g35300 // LOC_Os07g48310 // LOC_Os07g39520 // LOC_Os05g07090 // LOC_Os04g24510 // LOC_Os11g02240 // LOC_Os02g57700 // LOC_Os07g35140 // LOC_Os04g35240 // LOC_Os07g35004 // LOC_Os03g12520 // LOC_Os01g70410 // LOC_Os07g35540 // LOC_Os01g56330 // LOC_Os12g02200 // LOC_Os01g59580 // LOC_Os05g51190 // LOC_Os03g08550 // LOC_Os01g12430 // LOC_Os03g60810 // LOC_Os01g65650 // LOC_Os01g47470 // LOC_Os07g35790 // LOC_Os09g39620 // LOC_Os02g48080 // LOC_Os07g05740 // LOC_Os11g10710 // LOC_Os01g02840 // LOC_Os01g04480 // LOC_Os04g52630 // LOC_Os01g12290 // LOC_Os07g48730 // LOC_Os06g10520 // LOC_Os01g53640 // LOC_Os03g38710 // LOC_Os09g38910 // LOC_Os11g10280 // LOC_Os10g35040 // LOC_Os01g02390 // LOC_Os02g30540 // LOC_Os12g03650 // LOC_Os09g07730 // LOC_Os01g44110

GO:0003700 F transcription factor activity 46 778 682 24460 4e-06 0.00026 // LOC_Os04g46020 //

LOC_Os08g34360 // LOC_Os07g02060 // LOC_Os01g64790 // LOC_Os02g45450 // LOC_Os01g21120 // LOC_Os06g10880 // LOC_Os01g62310 // LOC_Os02g08440 // LOC_Os09g35030 // LOC_Os08g43210 // LOC_Os09g35010 // LOC_Os09g28354 // LOC_Os03g09170 // LOC_Os03g55990 // LOC_Os09g36910 // LOC_Os02g43790 // LOC_Os01g58760 // LOC_Os09g28440 // LOC_Os12g02400 // LOC_Os05g49100 // LOC_Os06g03670 // LOC_Os11g02470 // LOC_Os11g02520 // LOC_Os06g10780 // LOC_Os09g25060 // LOC_Os02g45420 // LOC_Os11g29870 // LOC_Os01g09100 // LOC_Os03g53340 // LOC_Os02g46500 // LOC_Os03g06630 // LOC_Os08g43334 // LOC_Os12g40920 // LOC_Os10g42490 // LOC_Os01g60600 // LOC_Os01g04800 // LOC_Os01g66270 // LOC_Os09g35020 // LOC_Os11g06170 // LOC_Os03g03164 // LOC_Os04g32620 // LOC_Os08g09840 // LOC_Os01g12440 // LOC_Os01g18584 // LOC_Os08g09900

GO:0004568 F chitinase activity 8 778 26 24460 7e-06 0.00038 // LOC_Os11g37950 //

LOC_Os06g51050 // LOC_Os06g51060 // LOC_Os09g32080 // LOC_Os08g41100 // LOC_Os10g39700 // LOC_Os10g39680 // LOC_Os11g37970

GO:0004672 F protein kinase activity 84 778 1587 24460 6.7e-06 0.00038 //

LOC_Os03g51440 // LOC_Os07g48760 // LOC_Os11g10280 // LOC_Os01g04480 // LOC_Os07g35300 // LOC_Os07g29330 // LOC_Os07g48310 // LOC_Os03g17700 // LOC_Os01g65650 // LOC_Os05g51190 // LOC_Os01g47470 // LOC_Os05g42210 // LOC_Os11g03840 // LOC_Os01g70130 // LOC_Os12g02200 // LOC_Os06g03970 // LOC_Os02g34600 // LOC_Os03g60810 // LOC_Os09g18360 // LOC_Os07g35790 // LOC_Os01g53640 // LOC_Os07g39520 // LOC_Os01g04230 // LOC_Os02g48210 // LOC_Os10g02250 // LOC_Os03g21510 // LOC_Os02g30540 // LOC_Os01g50410 // LOC_Os02g48080 // LOC_Os07g05740 // LOC_Os01g02770 // LOC_Os07g36544 // LOC_Os11g10710 // LOC_Os04g24510 // LOC_Os01g57560 // LOC_Os01g02790 // LOC_Os02g41580 // LOC_Os11g02240 // LOC_Os01g54700 // LOC_Os01g02840 // LOC_Os05g07090 // LOC_Os02g57700 // LOC_Os03g16010 // LOC_Os01g02760 // LOC_Os07g35140 // LOC_Os04g52630 // LOC_Os04g01310 // LOC_Os04g35240 // LOC_Os07g35004 // LOC_Os03g16260 // LOC_Os07g48730 // LOC_Os01g12290 // LOC_Os10g35040 // LOC_Os06g38970 // LOC_Os01g68870 // LOC_Os09g39650 // LOC_Os01g56330 // LOC_Os12g27520 // LOC_Os03g17980 // LOC_Os03g12520 // LOC_Os03g38710 // LOC_Os09g39620 // LOC_Os01g12430 // LOC_Os09g38910 // LOC_Os07g43570 // LOC_Os01g59580 // LOC_Os01g70410 // LOC_Os12g41410 // LOC_Os03g35600 // LOC_Os07g35540 // LOC_Os01g02400 // LOC_Os11g36160 // LOC_Os10g30540 // LOC_Os06g10790 // LOC_Os03g08550 // LOC_Os01g04450 // LOC_Os01g02390 // LOC_Os04g39930 // LOC_Os03g03410 // LOC_Os03g06330 // LOC_Os10g30530 // LOC_Os12g03650 // LOC_Os09g07730 // LOC_Os01g44110

GO:0005506 F iron ion binding 47 778 733 24460 1.1e-05 0.00054 //

LOC_Os12g04480 // LOC_Os11g05380 // LOC_Os07g44130 // LOC_Os04g59190 // LOC_Os07g44140 // LOC_Os01g25820 // LOC_Os10g30410 // LOC_Os03g63310 // LOC_Os07g47990 // LOC_Os05g41440 // LOC_Os01g57730 // LOC_Os07g48050 // LOC_Os01g22336 // LOC_Os03g56820 // LOC_Os01g16450 // LOC_Os01g53294 // LOC_Os08g35210 // LOC_Os09g26660 // LOC_Os12g01530 // LOC_Os08g41090 // LOC_Os11g01530 // LOC_Os02g56930 // LOC_Os01g43851 // LOC_Os10g38110 // LOC_Os02g10120 // LOC_Os10g08474 // LOC_Os01g43700 // LOC_Os10g36960 // LOC_Os02g47470 // LOC_Os07g33600 // LOC_Os07g23570 // LOC_Os08g01490 // LOC_Os05g03640 // LOC_Os04g59200 // LOC_Os07g18120 // LOC_Os01g59000 // LOC_Os01g61610 // LOC_Os04g40460 // LOC_Os07g02440 // LOC_Os08g39840 // LOC_Os03g12660 // LOC_Os02g12890 // LOC_Os05g41590 // LOC_Os03g44740 // LOC_Os02g57810 // LOC_Os04g14680 // LOC_Os04g36720

GO:0050662 F coenzyme binding 30 778 380 24460 1.3e-05 0.0006 //

LOC_Os10g33980 // LOC_Os08g04630 // LOC_Os01g25820 // LOC_Os10g01044 // LOC_Os05g07090 // LOC_Os01g39830 // LOC_Os01g53294 // LOC_Os08g35210 // LOC_Os04g53810 // LOC_Os09g26660 // LOC_Os12g25690 // LOC_Os11g37890 // LOC_Os03g55070 // LOC_Os07g42440 // LOC_Os03g57120 // LOC_Os04g14690 // LOC_Os01g27240 // LOC_Os09g37690 // LOC_Os06g44180 // LOC_Os02g40840 // LOC_Os05g32710 // LOC_Os07g18120 // LOC_Os03g22780 // LOC_Os07g41060 // LOC_Os05g41590 // LOC_Os01g27230 // LOC_Os04g36720 // LOC_Os04g52730 // LOC_Os03g17230 // LOC_Os06g46920

GO:0030246 F carbohydrate binding 27 778 327 24460 1.7e-05 0.00072 //

LOC_Os03g60810 // LOC_Os01g72810 // LOC_Os05g42210 // LOC_Os11g03840 // LOC_Os01g39830 // LOC_Os03g18910 // LOC_Os01g60480 // LOC_Os02g48210 // LOC_Os06g38160 // LOC_Os07g36544 // LOC_Os01g65030 // LOC_Os01g57560 // LOC_Os04g01310 // LOC_Os01g12290 // LOC_Os10g30540 // LOC_Os10g35460 // LOC_Os04g39930 // LOC_Os06g51050 // LOC_Os03g35600 // LOC_Os06g51060 // LOC_Os05g32110 // LOC_Os06g10790 // LOC_Os11g10280 // LOC_Os01g12430 // LOC_Os10g30530 // LOC_Os12g03650 // LOC_Os09g07730

GO:0016491 F oxidoreductase activity 97 778 1995 24460 3.3e-05 0.0013 //

LOC_Os12g04480 // LOC_Os11g05380 // LOC_Os07g44130 // LOC_Os03g63310 // LOC_Os02g39490 // LOC_Os05g07090 // LOC_Os07g48050 // LOC_Os04g57550 // LOC_Os04g58200 // LOC_Os04g40874 // LOC_Os03g29240 // LOC_Os02g50350 // LOC_Os03g55070 // LOC_Os07g42440 // LOC_Os10g38110 // LOC_Os04g39980 // LOC_Os01g43851 // LOC_Os03g32470 // LOC_Os01g43700 // LOC_Os12g12514 // LOC_Os09g37690 // LOC_Os03g53340 // LOC_Os02g40840 // LOC_Os07g23570 // LOC_Os05g11810 // LOC_Os01g51140 // LOC_Os04g14690 // LOC_Os07g44140 // LOC_Os08g32170 // LOC_Os01g27230 // LOC_Os10g30410 // LOC_Os03g57120 // LOC_Os01g61760 // LOC_Os01g22336 // LOC_Os01g16450 // LOC_Os01g39830 // LOC_Os01g53294 // LOC_Os09g26660 // LOC_Os12g25690 // LOC_Os12g07810 // LOC_Os10g36960 // LOC_Os03g07440 // LOC_Os02g47470 // LOC_Os01g51410 // LOC_Os07g33600 // LOC_Os01g59000 // LOC_Os01g61610 // LOC_Os02g12890 // LOC_Os03g18060 // LOC_Os03g44740 // LOC_Os08g16910 // LOC_Os01g70930 // LOC_Os07g46846 // LOC_Os05g41440 // LOC_Os08g01490 // LOC_Os03g56820 // LOC_Os08g35210 // LOC_Os12g01530 // LOC_Os08g04630 // LOC_Os06g03830 // LOC_Os10g39140 // LOC_Os12g15530 // LOC_Os02g10120 // LOC_Os10g08474 // LOC_Os01g61380 // LOC_Os07g02440 // LOC_Os02g51370 // LOC_Os07g46460 // LOC_Os04g59200 // LOC_Os08g33720 // LOC_Os03g03034 // LOC_Os05g41590 // LOC_Os07g47990 // LOC_Os04g41960 // LOC_Os02g57810 // LOC_Os11g32030 // LOC_Os04g36720 // LOC_Os04g59190 // LOC_Os01g25820 // LOC_Os01g57730 // LOC_Os04g22660 // LOC_Os04g20164 // LOC_Os01g65830 // LOC_Os08g41090 // LOC_Os05g48930 // LOC_Os01g27240 // LOC_Os01g62880 // LOC_Os11g01530 // LOC_Os05g03640 // LOC_Os12g27830 // LOC_Os09g20440 // LOC_Os04g40460 // LOC_Os08g39840 // LOC_Os03g12660 // LOC_Os01g39860 // LOC_Os07g18120 // LOC_Os04g14680

GO:0048037 F cofactor binding 37 778 547 24460 3.3e-05 0.0013 //

LOC_Os10g33980 // LOC_Os10g37340 // LOC_Os03g51740 // LOC_Os08g04630 // LOC_Os01g25820 // LOC_Os10g01044 // LOC_Os05g07090 // LOC_Os01g39830 // LOC_Os01g53294 // LOC_Os08g35210 // LOC_Os04g53810 // LOC_Os09g26660 // LOC_Os12g25690 // LOC_Os03g55090 // LOC_Os11g37890 // LOC_Os03g55070 // LOC_Os07g42440 // LOC_Os04g20164 // LOC_Os03g57120 // LOC_Os01g27240 // LOC_Os09g37690 // LOC_Os01g51410 // LOC_Os06g44180 // LOC_Os02g40840 // LOC_Os05g32710 // LOC_Os01g51980 // LOC_Os04g14690 // LOC_Os03g22780 // LOC_Os07g41060 // LOC_Os12g42980 // LOC_Os07g18120 // LOC_Os05g41590 // LOC_Os01g27230 // LOC_Os04g36720 // LOC_Os04g52730 // LOC_Os03g17230 // LOC_Os06g46920

GO:0005529 F sugar binding 22 778 272 24460 0.00013 0.0047 //

LOC_Os03g60810 // LOC_Os05g42210 // LOC_Os11g03840 // LOC_Os01g39830 // LOC_Os01g60480 // LOC_Os02g48210 // LOC_Os06g38160 // LOC_Os07g36544 // LOC_Os01g65030 // LOC_Os01g57560 // LOC_Os04g01310 // LOC_Os01g12290 // LOC_Os10g30540 // LOC_Os11g10280 // LOC_Os04g39930 // LOC_Os03g35600 // LOC_Os01g72810 // LOC_Os06g10790 // LOC_Os01g12430 // LOC_Os10g30530 // LOC_Os12g03650 // LOC_Os09g07730

GO:0016209 F antioxidant activity 16 778 219 24460 0.0027 0.074 //

LOC_Os05g41590 // LOC_Os08g41090 // LOC_Os01g25820 // LOC_Os04g14680 // LOC_Os03g29240 // LOC_Os07g47990 // LOC_Os04g59190 // LOC_Os01g57730 // LOC_Os07g48050 // LOC_Os01g22336 // LOC_Os04g59200 // LOC_Os01g16450 // LOC_Os07g02440 // LOC_Os01g53294 // LOC_Os08g35210 // LOC_Os09g26660

GO:0020037 F heme binding 34 778 622 24460 0.0024 0.074 //

LOC_Os12g04480 // LOC_Os11g05380 // LOC_Os07g44130 // LOC_Os04g59190 // LOC_Os07g44140 // LOC_Os10g30410 // LOC_Os03g63310 // LOC_Os07g47990 // LOC_Os05g41440 // LOC_Os01g57730 // LOC_Os07g48050 // LOC_Os01g22336 // LOC_Os01g16450 // LOC_Os08g41090 // LOC_Os02g56930 // LOC_Os01g43851 // LOC_Os10g38110 // LOC_Os10g08474 // LOC_Os01g43700 // LOC_Os07g02440 // LOC_Os02g47470 // LOC_Os07g33600 // LOC_Os07g23570 // LOC_Os08g01490 // LOC_Os04g59200 // LOC_Os01g59000 // LOC_Os04g40460 // LOC_Os10g36960 // LOC_Os03g12660 // LOC_Os02g12890 // LOC_Os05g41590 // LOC_Os03g44740 // LOC_Os02g57810 // LOC_Os04g14680

GO:0017076 F purine nucleotide binding 137 778 3394 24460 0.0024 0.074 //

LOC_Os05g30220 // LOC_Os03g16010 // LOC_Os03g17700 // LOC_Os01g68870 // LOC_Os05g42210 // LOC_Os11g03840 // LOC_Os01g70130 // LOC_Os02g48210 // LOC_Os04g13210 // LOC_Os07g29330 // LOC_Os02g47020 // LOC_Os06g03970 // LOC_Os09g37690 // LOC_Os01g02790 // LOC_Os05g07090 // LOC_Os01g04230 // LOC_Os03g16260 // LOC_Os07g48730 // LOC_Os04g30930 // LOC_Os07g18120 // LOC_Os04g39930 // LOC_Os08g42700 // LOC_Os03g53660 // LOC_Os10g02250 // LOC_Os08g41340 // LOC_Os05g04600 // LOC_Os03g35600 // LOC_Os02g34600 // LOC_Os11g36160 // LOC_Os04g56970 // LOC_Os01g02760 // LOC_Os03g51440 // LOC_Os03g06330 // LOC_Os10g30530 // LOC_Os12g17430 // LOC_Os08g34050 // LOC_Os03g57120 // LOC_Os11g29990 // LOC_Os01g53294 // LOC_Os12g41410 // LOC_Os01g02400 // LOC_Os09g26660 // LOC_Os10g07978 // LOC_Os04g56580 // LOC_Os01g59150 // LOC_Os01g18670 // LOC_Os03g21510 // LOC_Os01g50410 // LOC_Os01g02770 // LOC_Os07g36544 // LOC_Os01g57560 // LOC_Os02g41580 // LOC_Os11g22350 // LOC_Os04g01310 // LOC_Os03g03410 // LOC_Os10g30540 // LOC_Os01g54700 // LOC_Os01g25710 // LOC_Os09g39650 // LOC_Os12g27520 // LOC_Os03g17980 // LOC_Os03g53800 // LOC_Os07g43570 // LOC_Os02g48360 // LOC_Os06g05359 // LOC_Os06g10790 // LOC_Os01g04450 // LOC_Os03g44420 // LOC_Os01g62500 // LOC_Os07g48760 // LOC_Os08g10260 // LOC_Os07g35300 // LOC_Os07g48310 // LOC_Os06g08790 // LOC_Os04g57350 // LOC_Os08g35210 // LOC_Os07g39520 // LOC_Os08g04630 // LOC_Os05g44340 // LOC_Os04g24510 // LOC_Os11g02240 // LOC_Os02g57700 // LOC_Os07g12900 // LOC_Os07g35140 // LOC_Os04g35240 // LOC_Os07g35004 // LOC_Os03g12520 // LOC_Os01g70410 // LOC_Os07g35540 // LOC_Os01g56330 // LOC_Os12g02200 // LOC_Os02g09100 // LOC_Os06g12790 // LOC_Os01g59580 // LOC_Os05g33570 // LOC_Os10g04290 // LOC_Os05g51190 // LOC_Os03g08550 // LOC_Os01g62950 // LOC_Os04g36720 // LOC_Os01g12430 // LOC_Os03g60810 // LOC_Os01g25820 // LOC_Os01g45450 // LOC_Os01g65650 // LOC_Os01g47470 // LOC_Os10g37500 // LOC_Os02g19750 // LOC_Os07g35790 // LOC_Os02g40840 // LOC_Os02g07060 // LOC_Os02g48080 // LOC_Os07g05740 // LOC_Os11g10710 // LOC_Os04g14690 // LOC_Os01g02840 // LOC_Os01g04480 // LOC_Os03g58790 // LOC_Os04g52630 // LOC_Os09g34160 // LOC_Os03g58800 // LOC_Os01g12290 // LOC_Os09g18360 // LOC_Os06g10520 // LOC_Os01g53640 // LOC_Os03g38710 // LOC_Os09g39620 // LOC_Os09g38910 // LOC_Os06g38970 // LOC_Os11g10280 // LOC_Os10g35040 // LOC_Os01g05620 // LOC_Os01g02390 // LOC_Os02g30540 // LOC_Os12g03650 // LOC_Os09g07730 // LOC_Os01g44110

GO:0043565 F sequence-specific DNA binding 27 778 455 24460 0.0023 0.074 //

LOC_Os04g46020 // LOC_Os07g02060 // LOC_Os01g09100 // LOC_Os06g10880 // LOC_Os01g62310 // LOC_Os02g08440 // LOC_Os09g28354 // LOC_Os09g36910 // LOC_Os01g58760 // LOC_Os12g02400 // LOC_Os03g55990 // LOC_Os11g02470 // LOC_Os11g02520 // LOC_Os09g25060 // LOC_Os11g29870 // LOC_Os03g53340 // LOC_Os03g06630 // LOC_Os08g43334 // LOC_Os12g40920 // LOC_Os10g42490 // LOC_Os01g60600 // LOC_Os05g49100 // LOC_Os11g06170 // LOC_Os03g03164 // LOC_Os08g09840 // LOC_Os01g18584 // LOC_Os08g09900

GO:0046906 F tetrapyrrole binding 34 778 629 24460 0.0028 0.074 //

LOC_Os12g04480 // LOC_Os11g05380 // LOC_Os07g44130 // LOC_Os04g59190 // LOC_Os07g44140 // LOC_Os10g30410 // LOC_Os03g63310 // LOC_Os07g47990 // LOC_Os05g41440 // LOC_Os01g57730 // LOC_Os07g48050 // LOC_Os01g22336 // LOC_Os01g16450 // LOC_Os08g41090 // LOC_Os02g56930 // LOC_Os01g43851 // LOC_Os10g38110 // LOC_Os10g08474 // LOC_Os01g43700 // LOC_Os07g02440 // LOC_Os02g47470 // LOC_Os07g33600 // LOC_Os07g23570 // LOC_Os08g01490 // LOC_Os04g59200 // LOC_Os01g59000 // LOC_Os04g40460 // LOC_Os10g36960 // LOC_Os03g12660 // LOC_Os02g12890 // LOC_Os05g41590 // LOC_Os03g44740 // LOC_Os02g57810 // LOC_Os04g14680

GO:0030554 F adenyl nucleotide binding 130 778 3210 24460 0.0028 0.074 //

LOC_Os05g30220 // LOC_Os03g16010 // LOC_Os03g17700 // LOC_Os01g68870 // LOC_Os05g42210 // LOC_Os11g03840 // LOC_Os01g70130 // LOC_Os02g48210 // LOC_Os04g13210 // LOC_Os07g29330 // LOC_Os02g47020 // LOC_Os06g03970 // LOC_Os09g37690 // LOC_Os01g02790 // LOC_Os05g07090 // LOC_Os01g04230 // LOC_Os03g16260 // LOC_Os07g48730 // LOC_Os04g30930 // LOC_Os07g18120 // LOC_Os04g39930 // LOC_Os08g42700 // LOC_Os03g53660 // LOC_Os10g02250 // LOC_Os05g04600 // LOC_Os03g35600 // LOC_Os02g34600 // LOC_Os11g36160 // LOC_Os01g02760 // LOC_Os03g51440 // LOC_Os03g06330 // LOC_Os10g30530 // LOC_Os12g17430 // LOC_Os08g34050 // LOC_Os03g57120 // LOC_Os11g29990 // LOC_Os01g53294 // LOC_Os12g41410 // LOC_Os01g02400 // LOC_Os09g26660 // LOC_Os10g07978 // LOC_Os04g56580 // LOC_Os01g18670 // LOC_Os03g21510 // LOC_Os01g50410 // LOC_Os01g02770 // LOC_Os07g36544 // LOC_Os01g57560 // LOC_Os02g41580 // LOC_Os11g22350 // LOC_Os04g01310 // LOC_Os03g03410 // LOC_Os10g30540 // LOC_Os01g54700 // LOC_Os01g25710 // LOC_Os09g39650 // LOC_Os12g27520 // LOC_Os03g17980 // LOC_Os03g53800 // LOC_Os07g43570 // LOC_Os02g48360 // LOC_Os06g05359 // LOC_Os06g10790 // LOC_Os01g04450 // LOC_Os01g62500 // LOC_Os07g48760 // LOC_Os08g10260 // LOC_Os07g35300 // LOC_Os07g48310 // LOC_Os06g08790 // LOC_Os08g35210 // LOC_Os07g39520 // LOC_Os08g04630 // LOC_Os05g44340 // LOC_Os04g24510 // LOC_Os11g02240 // LOC_Os02g57700 // LOC_Os07g12900 // LOC_Os07g35140 // LOC_Os04g35240 // LOC_Os07g35004 // LOC_Os03g12520 // LOC_Os01g70410 // LOC_Os07g35540 // LOC_Os01g56330 // LOC_Os12g02200 // LOC_Os02g09100 // LOC_Os01g59580 // LOC_Os05g33570 // LOC_Os10g04290 // LOC_Os05g51190 // LOC_Os03g08550 // LOC_Os01g62950 // LOC_Os04g36720 // LOC_Os01g12430 // LOC_Os03g60810 // LOC_Os01g25820 // LOC_Os01g45450 // LOC_Os01g65650 // LOC_Os01g47470 // LOC_Os10g37500 // LOC_Os02g19750 // LOC_Os07g35790 // LOC_Os02g40840 // LOC_Os02g48080 // LOC_Os07g05740 // LOC_Os11g10710 // LOC_Os04g14690 // LOC_Os01g02840 // LOC_Os01g04480 // LOC_Os03g58790 // LOC_Os04g52630 // LOC_Os09g34160 // LOC_Os03g58800 // LOC_Os01g12290 // LOC_Os09g18360 // LOC_Os06g10520 // LOC_Os01g53640 // LOC_Os03g38710 // LOC_Os09g39620 // LOC_Os09g38910 // LOC_Os06g38970 // LOC_Os11g10280 // LOC_Os10g35040 // LOC_Os01g05620 // LOC_Os01g02390 // LOC_Os02g30540 // LOC_Os12g03650 // LOC_Os09g07730 // LOC_Os01g44110

GO:0001883 F purine nucleoside binding 130 778 3210 24460 0.0028 0.074 //

LOC_Os05g30220 // LOC_Os03g16010 // LOC_Os03g17700 // LOC_Os01g68870 // LOC_Os05g42210 // LOC_Os11g03840 // LOC_Os01g70130 // LOC_Os02g48210 // LOC_Os04g13210 // LOC_Os07g29330 // LOC_Os02g47020 // LOC_Os06g03970 // LOC_Os09g37690 // LOC_Os01g02790 // LOC_Os05g07090 // LOC_Os01g04230 // LOC_Os03g16260 // LOC_Os07g48730 // LOC_Os04g30930 // LOC_Os07g18120 // LOC_Os04g39930 // LOC_Os08g42700 // LOC_Os03g53660 // LOC_Os10g02250 // LOC_Os05g04600 // LOC_Os03g35600 // LOC_Os02g34600 // LOC_Os11g36160 // LOC_Os01g02760 // LOC_Os03g51440 // LOC_Os03g06330 // LOC_Os10g30530 // LOC_Os12g17430 // LOC_Os08g34050 // LOC_Os03g57120 // LOC_Os11g29990 // LOC_Os01g53294 // LOC_Os12g41410 // LOC_Os01g02400 // LOC_Os09g26660 // LOC_Os10g07978 // LOC_Os04g56580 // LOC_Os01g18670 // LOC_Os03g21510 // LOC_Os01g50410 // LOC_Os01g02770 // LOC_Os07g36544 // LOC_Os01g57560 // LOC_Os02g41580 // LOC_Os11g22350 // LOC_Os04g01310 // LOC_Os03g03410 // LOC_Os10g30540 // LOC_Os01g54700 // LOC_Os01g25710 // LOC_Os09g39650 // LOC_Os12g27520 // LOC_Os03g17980 // LOC_Os03g53800 // LOC_Os07g43570 // LOC_Os02g48360 // LOC_Os06g05359 // LOC_Os06g10790 // LOC_Os01g04450 // LOC_Os01g62500 // LOC_Os07g48760 // LOC_Os08g10260 // LOC_Os07g35300 // LOC_Os07g48310 // LOC_Os06g08790 // LOC_Os08g35210 // LOC_Os07g39520 // LOC_Os08g04630 // LOC_Os05g44340 // LOC_Os04g24510 // LOC_Os11g02240 // LOC_Os02g57700 // LOC_Os07g12900 // LOC_Os07g35140 // LOC_Os04g35240 // LOC_Os07g35004 // LOC_Os03g12520 // LOC_Os01g70410 // LOC_Os07g35540 // LOC_Os01g56330 // LOC_Os12g02200 // LOC_Os02g09100 // LOC_Os01g59580 // LOC_Os05g33570 // LOC_Os10g04290 // LOC_Os05g51190 // LOC_Os03g08550 // LOC_Os01g62950 // LOC_Os04g36720 // LOC_Os01g12430 // LOC_Os03g60810 // LOC_Os01g25820 // LOC_Os01g45450 // LOC_Os01g65650 // LOC_Os01g47470 // LOC_Os10g37500 // LOC_Os02g19750 // LOC_Os07g35790 // LOC_Os02g40840 // LOC_Os02g48080 // LOC_Os07g05740 // LOC_Os11g10710 // LOC_Os04g14690 // LOC_Os01g02840 // LOC_Os01g04480 // LOC_Os03g58790 // LOC_Os04g52630 // LOC_Os09g34160 // LOC_Os03g58800 // LOC_Os01g12290 // LOC_Os09g18360 // LOC_Os06g10520 // LOC_Os01g53640 // LOC_Os03g38710 // LOC_Os09g39620 // LOC_Os09g38910 // LOC_Os06g38970 // LOC_Os11g10280 // LOC_Os10g35040 // LOC_Os01g05620 // LOC_Os01g02390 // LOC_Os02g30540 // LOC_Os12g03650 // LOC_Os09g07730 // LOC_Os01g44110

GO:0001882 F nucleoside binding 130 778 3220 24460 0.0031 0.074 //

LOC_Os05g30220 // LOC_Os03g16010 // LOC_Os03g17700 // LOC_Os01g68870 // LOC_Os05g42210 // LOC_Os11g03840 // LOC_Os01g70130 // LOC_Os02g48210 // LOC_Os04g13210 // LOC_Os07g29330 // LOC_Os02g47020 // LOC_Os06g03970 // LOC_Os09g37690 // LOC_Os01g02790 // LOC_Os05g07090 // LOC_Os01g04230 // LOC_Os03g16260 // LOC_Os07g48730 // LOC_Os04g30930 // LOC_Os07g18120 // LOC_Os04g39930 // LOC_Os08g42700 // LOC_Os03g53660 // LOC_Os10g02250 // LOC_Os05g04600 // LOC_Os03g35600 // LOC_Os02g34600 // LOC_Os11g36160 // LOC_Os01g02760 // LOC_Os03g51440 // LOC_Os03g06330 // LOC_Os10g30530 // LOC_Os12g17430 // LOC_Os08g34050 // LOC_Os03g57120 // LOC_Os11g29990 // LOC_Os01g53294 // LOC_Os12g41410 // LOC_Os01g02400 // LOC_Os09g26660 // LOC_Os10g07978 // LOC_Os04g56580 // LOC_Os01g18670 // LOC_Os03g21510 // LOC_Os01g50410 // LOC_Os01g02770 // LOC_Os07g36544 // LOC_Os01g57560 // LOC_Os02g41580 // LOC_Os11g22350 // LOC_Os04g01310 // LOC_Os03g03410 // LOC_Os10g30540 // LOC_Os01g54700 // LOC_Os01g25710 // LOC_Os09g39650 // LOC_Os12g27520 // LOC_Os03g17980 // LOC_Os03g53800 // LOC_Os07g43570 // LOC_Os02g48360 // LOC_Os06g05359 // LOC_Os06g10790 // LOC_Os01g04450 // LOC_Os01g62500 // LOC_Os07g48760 // LOC_Os08g10260 // LOC_Os07g35300 // LOC_Os07g48310 // LOC_Os06g08790 // LOC_Os08g35210 // LOC_Os07g39520 // LOC_Os08g04630 // LOC_Os05g44340 // LOC_Os04g24510 // LOC_Os11g02240 // LOC_Os02g57700 // LOC_Os07g12900 // LOC_Os07g35140 // LOC_Os04g35240 // LOC_Os07g35004 // LOC_Os03g12520 // LOC_Os01g70410 // LOC_Os07g35540 // LOC_Os01g56330 // LOC_Os12g02200 // LOC_Os02g09100 // LOC_Os01g59580 // LOC_Os05g33570 // LOC_Os10g04290 // LOC_Os05g51190 // LOC_Os03g08550 // LOC_Os01g62950 // LOC_Os04g36720 // LOC_Os01g12430 // LOC_Os03g60810 // LOC_Os01g25820 // LOC_Os01g45450 // LOC_Os01g65650 // LOC_Os01g47470 // LOC_Os10g37500 // LOC_Os02g19750 // LOC_Os07g35790 // LOC_Os02g40840 // LOC_Os02g48080 // LOC_Os07g05740 // LOC_Os11g10710 // LOC_Os04g14690 // LOC_Os01g02840 // LOC_Os01g04480 // LOC_Os03g58790 // LOC_Os04g52630 // LOC_Os09g34160 // LOC_Os03g58800 // LOC_Os01g12290 // LOC_Os09g18360 // LOC_Os06g10520 // LOC_Os01g53640 // LOC_Os03g38710 // LOC_Os09g39620 // LOC_Os09g38910 // LOC_Os06g38970 // LOC_Os11g10280 // LOC_Os10g35040 // LOC_Os01g05620 // LOC_Os01g02390 // LOC_Os02g30540 // LOC_Os12g03650 // LOC_Os09g07730 // LOC_Os01g44110

GO:0016684 F oxidoreductase activity, acting on peroxide as acceptor 15 778 201 24460 0.003 0.074 //

LOC_Os08g41090 // LOC_Os01g25820 // LOC_Os04g14680 // LOC_Os05g41590 // LOC_Os07g47990 // LOC_Os04g59190 // LOC_Os01g57730 // LOC_Os07g48050 // LOC_Os01g22336 // LOC_Os04g59200 // LOC_Os01g16450 // LOC_Os07g02440 // LOC_Os01g53294 // LOC_Os08g35210 // LOC_Os09g26660

GO:0004601 F peroxidase activity 15 778 201 24460 0.003 0.074 //

LOC_Os08g41090 // LOC_Os01g25820 // LOC_Os04g14680 // LOC_Os05g41590 // LOC_Os07g47990 // LOC_Os04g59190 // LOC_Os01g57730 // LOC_Os07g48050 // LOC_Os01g22336 // LOC_Os04g59200 // LOC_Os01g16450 // LOC_Os07g02440 // LOC_Os01g53294 // LOC_Os08g35210 // LOC_Os09g26660

GO:0016762 F xyloglucan: xyloglucosyl transferase activity 5 778 30 24460 0.0041 0.096 //

LOC_Os04g51460 // LOC_Os06g48160 // LOC_Os02g46910 // LOC_Os06g48200 // LOC_Os03g13570

GO:0050660 F FAD binding 12 778 153 24460 0.0052 0.12 //

LOC_Os05g07090 // LOC_Os01g25820 // LOC_Os02g40840 // LOC_Os07g18120 // LOC_Os03g57120 // LOC_Os08g04630 // LOC_Os01g53294 // LOC_Os04g14690 // LOC_Os08g35210 // LOC_Os09g37690 // LOC_Os04g36720 // LOC_Os09g26660

GO:0004497 F monooxygenase activity 24 778 423 24460 0.0065 0.14 //

LOC_Os12g04480 // LOC_Os11g05380 // LOC_Os07g44130 // LOC_Os04g40460 // LOC_Os10g30410 // LOC_Os03g63310 // LOC_Os05g41440 // LOC_Os08g01490 // LOC_Os10g08474 // LOC_Os10g38110 // LOC_Os01g43851 // LOC_Os01g43700 // LOC_Os04g14690 // LOC_Os10g36960 // LOC_Os09g37690 // LOC_Os02g47470 // LOC_Os07g33600 // LOC_Os07g23570 // LOC_Os01g59000 // LOC_Os07g44140 // LOC_Os03g12660 // LOC_Os02g12890 // LOC_Os03g44740 // LOC_Os02g57810

GO:0000166 F nucleotide binding 147 778 3794 24460 0.007 0.15 //

LOC_Os05g30220 // LOC_Os03g16010 // LOC_Os03g17700 // LOC_Os01g68870 // LOC_Os05g42210 // LOC_Os11g03840 // LOC_Os01g70130 // LOC_Os02g48210 // LOC_Os04g13210 // LOC_Os03g55070 // LOC_Os07g42440 // LOC_Os02g47020 // LOC_Os06g03970 // LOC_Os09g37690 // LOC_Os01g02790 // LOC_Os05g07090 // LOC_Os01g04230 // LOC_Os03g16260 // LOC_Os07g48730 // LOC_Os04g30930 // LOC_Os07g18120 // LOC_Os04g39930 // LOC_Os08g42700 // LOC_Os03g53660 // LOC_Os10g02250 // LOC_Os08g41340 // LOC_Os05g04600 // LOC_Os03g35600 // LOC_Os02g34600 // LOC_Os11g36160 // LOC_Os01g27230 // LOC_Os04g56970 // LOC_Os01g02760 // LOC_Os03g51440 // LOC_Os03g06330 // LOC_Os10g30530 // LOC_Os12g17430 // LOC_Os08g34050 // LOC_Os03g57120 // LOC_Os11g29990 // LOC_Os01g53294 // LOC_Os12g41410 // LOC_Os01g02400 // LOC_Os09g26660 // LOC_Os10g07978 // LOC_Os04g56580 // LOC_Os12g25690 // LOC_Os01g18670 // LOC_Os03g21510 // LOC_Os01g50410 // LOC_Os04g14690 // LOC_Os01g02770 // LOC_Os07g36544 // LOC_Os01g57560 // LOC_Os02g41580 // LOC_Os11g22350 // LOC_Os04g01310 // LOC_Os03g03410 // LOC_Os10g30540 // LOC_Os01g54700 // LOC_Os01g25710 // LOC_Os09g39650 // LOC_Os12g27520 // LOC_Os03g17980 // LOC_Os03g53800 // LOC_Os07g43570 // LOC_Os02g48360 // LOC_Os06g05359 // LOC_Os02g51890 // LOC_Os06g10790 // LOC_Os01g04450 // LOC_Os03g44420 // LOC_Os01g62500 // LOC_Os01g65550 // LOC_Os07g48760 // LOC_Os08g10260 // LOC_Os07g35300 // LOC_Os07g29330 // LOC_Os07g48310 // LOC_Os06g08790 // LOC_Os04g57350 // LOC_Os08g35210 // LOC_Os07g39520 // LOC_Os08g04630 // LOC_Os05g44340 // LOC_Os04g24510 // LOC_Os11g02240 // LOC_Os02g57700 // LOC_Os07g12900 // LOC_Os07g35140 // LOC_Os04g35240 // LOC_Os07g35004 // LOC_Os03g12520 // LOC_Os01g70410 // LOC_Os07g35540 // LOC_Os01g56330 // LOC_Os12g02200 // LOC_Os02g09100 // LOC_Os06g12790 // LOC_Os05g13620 // LOC_Os01g59580 // LOC_Os05g33570 // LOC_Os05g41590 // LOC_Os10g04290 // LOC_Os05g51190 // LOC_Os03g08550 // LOC_Os01g62950 // LOC_Os04g36720 // LOC_Os01g12430 // LOC_Os03g60810 // LOC_Os01g25820 // LOC_Os01g45450 // LOC_Os01g65650 // LOC_Os01g47470 // LOC_Os10g37500 // LOC_Os02g19750 // LOC_Os07g35790 // LOC_Os02g40840 // LOC_Os02g07060 // LOC_Os02g48080 // LOC_Os07g05740 // LOC_Os11g10710 // LOC_Os01g27240 // LOC_Os01g02840 // LOC_Os01g04480 // LOC_Os03g58790 // LOC_Os04g52630 // LOC_Os09g34160 // LOC_Os03g58800 // LOC_Os01g12290 // LOC_Os09g18360 // LOC_Os01g11130 // LOC_Os06g10520 // LOC_Os01g53640 // LOC_Os03g38710 // LOC_Os01g59150 // LOC_Os09g39620 // LOC_Os09g38910 // LOC_Os06g38970 // LOC_Os11g10280 // LOC_Os10g35040 // LOC_Os01g05620 // LOC_Os01g02390 // LOC_Os02g30540 // LOC_Os12g03650 // LOC_Os09g07730 // LOC_Os01g44110

GO:0032555 F purine ribonucleotide binding 126 778 3238 24460 0.011 0.22 //

LOC_Os05g30220 // LOC_Os03g16010 // LOC_Os03g17700 // LOC_Os01g68870 // LOC_Os05g42210 // LOC_Os11g03840 // LOC_Os01g70130 // LOC_Os02g48210 // LOC_Os03g44420 // LOC_Os07g29330 // LOC_Os02g47020 // LOC_Os06g03970 // LOC_Os01g02790 // LOC_Os05g07090 // LOC_Os01g04230 // LOC_Os03g16260 // LOC_Os07g48730 // LOC_Os04g30930 // LOC_Os06g38970 // LOC_Os04g39930 // LOC_Os08g42700 // LOC_Os03g53660 // LOC_Os10g02250 // LOC_Os08g41340 // LOC_Os05g04600 // LOC_Os03g35600 // LOC_Os02g34600 // LOC_Os11g36160 // LOC_Os04g56970 // LOC_Os01g02760 // LOC_Os03g51440 // LOC_Os03g06330 // LOC_Os10g30530 // LOC_Os12g17430 // LOC_Os08g34050 // LOC_Os12g41410 // LOC_Os01g02400 // LOC_Os10g07978 // LOC_Os04g56580 // LOC_Os01g59150 // LOC_Os01g18670 // LOC_Os03g21510 // LOC_Os01g50410 // LOC_Os01g02770 // LOC_Os07g36544 // LOC_Os01g57560 // LOC_Os02g41580 // LOC_Os11g22350 // LOC_Os04g01310 // LOC_Os03g03410 // LOC_Os10g30540 // LOC_Os01g54700 // LOC_Os09g39650 // LOC_Os12g27520 // LOC_Os03g17980 // LOC_Os03g53800 // LOC_Os07g43570 // LOC_Os02g48360 // LOC_Os06g05359 // LOC_Os06g10790 // LOC_Os01g04450 // LOC_Os04g13210 // LOC_Os01g62500 // LOC_Os07g48760 // LOC_Os08g10260 // LOC_Os07g35300 // LOC_Os07g48310 // LOC_Os06g08790 // LOC_Os04g57350 // LOC_Os11g29990 // LOC_Os07g39520 // LOC_Os05g44340 // LOC_Os04g24510 // LOC_Os11g02240 // LOC_Os02g57700 // LOC_Os07g12900 // LOC_Os07g35140 // LOC_Os04g35240 // LOC_Os07g35004 // LOC_Os03g12520 // LOC_Os01g70410 // LOC_Os07g35540 // LOC_Os01g56330 // LOC_Os12g02200 // LOC_Os02g09100 // LOC_Os06g12790 // LOC_Os01g59580 // LOC_Os05g33570 // LOC_Os10g04290 // LOC_Os05g51190 // LOC_Os03g08550 // LOC_Os01g62950 // LOC_Os01g25710 // LOC_Os01g12430 // LOC_Os03g60810 // LOC_Os01g45450 // LOC_Os01g65650 // LOC_Os01g47470 // LOC_Os10g37500 // LOC_Os02g19750 // LOC_Os07g35790 // LOC_Os09g39620 // LOC_Os02g07060 // LOC_Os02g48080 // LOC_Os07g05740 // LOC_Os11g10710 // LOC_Os01g02840 // LOC_Os01g04480 // LOC_Os03g58790 // LOC_Os04g52630 // LOC_Os09g34160 // LOC_Os03g58800 // LOC_Os01g12290 // LOC_Os09g18360 // LOC_Os06g10520 // LOC_Os01g53640 // LOC_Os03g38710 // LOC_Os09g38910 // LOC_Os11g10280 // LOC_Os10g35040 // LOC_Os01g05620 // LOC_Os01g02390 // LOC_Os02g30540 // LOC_Os12g03650 // LOC_Os09g07730 // LOC_Os01g44110

GO:0032553 F ribonucleotide binding 126 778 3238 24460 0.011 0.22 //

LOC_Os05g30220 // LOC_Os03g16010 // LOC_Os03g17700 // LOC_Os01g68870 // LOC_Os05g42210 // LOC_Os11g03840 // LOC_Os01g70130 // LOC_Os02g48210 // LOC_Os03g44420 // LOC_Os07g29330 // LOC_Os02g47020 // LOC_Os06g03970 // LOC_Os01g02790 // LOC_Os05g07090 // LOC_Os01g04230 // LOC_Os03g16260 // LOC_Os07g48730 // LOC_Os04g30930 // LOC_Os06g38970 // LOC_Os04g39930 // LOC_Os08g42700 // LOC_Os03g53660 // LOC_Os10g02250 // LOC_Os08g41340 // LOC_Os05g04600 // LOC_Os03g35600 // LOC_Os02g34600 // LOC_Os11g36160 // LOC_Os04g56970 // LOC_Os01g02760 // LOC_Os03g51440 // LOC_Os03g06330 // LOC_Os10g30530 // LOC_Os12g17430 // LOC_Os08g34050 // LOC_Os12g41410 // LOC_Os01g02400 // LOC_Os10g07978 // LOC_Os04g56580 // LOC_Os01g59150 // LOC_Os01g18670 // LOC_Os03g21510 // LOC_Os01g50410 // LOC_Os01g02770 // LOC_Os07g36544 // LOC_Os01g57560 // LOC_Os02g41580 // LOC_Os11g22350 // LOC_Os04g01310 // LOC_Os03g03410 // LOC_Os10g30540 // LOC_Os01g54700 // LOC_Os09g39650 // LOC_Os12g27520 // LOC_Os03g17980 // LOC_Os03g53800 // LOC_Os07g43570 // LOC_Os02g48360 // LOC_Os06g05359 // LOC_Os06g10790 // LOC_Os01g04450 // LOC_Os04g13210 // LOC_Os01g62500 // LOC_Os07g48760 // LOC_Os08g10260 // LOC_Os07g35300 // LOC_Os07g48310 // LOC_Os06g08790 // LOC_Os04g57350 // LOC_Os11g29990 // LOC_Os07g39520 // LOC_Os05g44340 // LOC_Os04g24510 // LOC_Os11g02240 // LOC_Os02g57700 // LOC_Os07g12900 // LOC_Os07g35140 // LOC_Os04g35240 // LOC_Os07g35004 // LOC_Os03g12520 // LOC_Os01g70410 // LOC_Os07g35540 // LOC_Os01g56330 // LOC_Os12g02200 // LOC_Os02g09100 // LOC_Os06g12790 // LOC_Os01g59580 // LOC_Os05g33570 // LOC_Os10g04290 // LOC_Os05g51190 // LOC_Os03g08550 // LOC_Os01g62950 // LOC_Os01g25710 // LOC_Os01g12430 // LOC_Os03g60810 // LOC_Os01g45450 // LOC_Os01g65650 // LOC_Os01g47470 // LOC_Os10g37500 // LOC_Os02g19750 // LOC_Os07g35790 // LOC_Os09g39620 // LOC_Os02g07060 // LOC_Os02g48080 // LOC_Os07g05740 // LOC_Os11g10710 // LOC_Os01g02840 // LOC_Os01g04480 // LOC_Os03g58790 // LOC_Os04g52630 // LOC_Os09g34160 // LOC_Os03g58800 // LOC_Os01g12290 // LOC_Os09g18360 // LOC_Os06g10520 // LOC_Os01g53640 // LOC_Os03g38710 // LOC_Os09g38910 // LOC_Os11g10280 // LOC_Os10g35040 // LOC_Os01g05620 // LOC_Os01g02390 // LOC_Os02g30540 // LOC_Os12g03650 // LOC_Os09g07730 // LOC_Os01g44110

GO:0005524 F ATP binding 119 778 3055 24460 0.013 0.25 //

LOC_Os05g30220 // LOC_Os03g16010 // LOC_Os03g17700 // LOC_Os01g68870 // LOC_Os05g42210 // LOC_Os11g03840 // LOC_Os01g70130 // LOC_Os02g48210 // LOC_Os04g13210 // LOC_Os07g29330 // LOC_Os02g47020 // LOC_Os06g03970 // LOC_Os01g02790 // LOC_Os05g07090 // LOC_Os01g04230 // LOC_Os03g16260 // LOC_Os09g18360 // LOC_Os04g30930 // LOC_Os06g38970 // LOC_Os04g39930 // LOC_Os08g42700 // LOC_Os03g53660 // LOC_Os10g02250 // LOC_Os05g04600 // LOC_Os03g35600 // LOC_Os02g34600 // LOC_Os11g36160 // LOC_Os01g02760 // LOC_Os03g51440 // LOC_Os03g06330 // LOC_Os10g30530 // LOC_Os12g17430 // LOC_Os08g34050 // LOC_Os12g41410 // LOC_Os01g02400 // LOC_Os10g07978 // LOC_Os04g56580 // LOC_Os01g18670 // LOC_Os03g21510 // LOC_Os01g50410 // LOC_Os01g02770 // LOC_Os07g36544 // LOC_Os01g57560 // LOC_Os02g41580 // LOC_Os11g22350 // LOC_Os04g01310 // LOC_Os03g03410 // LOC_Os10g30540 // LOC_Os01g54700 // LOC_Os09g39650 // LOC_Os12g27520 // LOC_Os03g17980 // LOC_Os03g53800 // LOC_Os07g43570 // LOC_Os02g48360 // LOC_Os06g05359 // LOC_Os06g10790 // LOC_Os01g04450 // LOC_Os01g62500 // LOC_Os07g48760 // LOC_Os08g10260 // LOC_Os07g35300 // LOC_Os07g48310 // LOC_Os06g08790 // LOC_Os11g29990 // LOC_Os07g39520 // LOC_Os05g44340 // LOC_Os04g24510 // LOC_Os11g02240 // LOC_Os02g57700 // LOC_Os07g12900 // LOC_Os07g35140 // LOC_Os04g35240 // LOC_Os07g35004 // LOC_Os03g12520 // LOC_Os01g70410 // LOC_Os07g35540 // LOC_Os01g56330 // LOC_Os12g02200 // LOC_Os02g09100 // LOC_Os01g59580 // LOC_Os05g33570 // LOC_Os10g04290 // LOC_Os05g51190 // LOC_Os03g08550 // LOC_Os01g62950 // LOC_Os01g25710 // LOC_Os01g12430 // LOC_Os03g60810 // LOC_Os01g45450 // LOC_Os01g65650 // LOC_Os01g47470 // LOC_Os10g37500 // LOC_Os02g19750 // LOC_Os07g35790 // LOC_Os09g39620 // LOC_Os02g48080 // LOC_Os07g05740 // LOC_Os11g10710 // LOC_Os01g02840 // LOC_Os01g04480 // LOC_Os03g58790 // LOC_Os04g52630 // LOC_Os09g34160 // LOC_Os03g58800 // LOC_Os01g12290 // LOC_Os07g48730 // LOC_Os06g10520 // LOC_Os01g53640 // LOC_Os03g38710 // LOC_Os09g38910 // LOC_Os11g10280 // LOC_Os10g35040 // LOC_Os01g05620 // LOC_Os01g02390 // LOC_Os02g30540 // LOC_Os12g03650 // LOC_Os09g07730 // LOC_Os01g44110

GO:0032559 F adenyl ribonucleotide binding 119 778 3058 24460 0.014 0.25 //

LOC_Os05g30220 // LOC_Os03g16010 // LOC_Os03g17700 // LOC_Os01g68870 // LOC_Os05g42210 // LOC_Os11g03840 // LOC_Os01g70130 // LOC_Os02g48210 // LOC_Os04g13210 // LOC_Os07g29330 // LOC_Os02g47020 // LOC_Os06g03970 // LOC_Os01g02790 // LOC_Os05g07090 // LOC_Os01g04230 // LOC_Os03g16260 // LOC_Os09g18360 // LOC_Os04g30930 // LOC_Os06g38970 // LOC_Os04g39930 // LOC_Os08g42700 // LOC_Os03g53660 // LOC_Os10g02250 // LOC_Os05g04600 // LOC_Os03g35600 // LOC_Os02g34600 // LOC_Os11g36160 // LOC_Os01g02760 // LOC_Os03g51440 // LOC_Os03g06330 // LOC_Os10g30530 // LOC_Os12g17430 // LOC_Os08g34050 // LOC_Os12g41410 // LOC_Os01g02400 // LOC_Os10g07978 // LOC_Os04g56580 // LOC_Os01g18670 // LOC_Os03g21510 // LOC_Os01g50410 // LOC_Os01g02770 // LOC_Os07g36544 // LOC_Os01g57560 // LOC_Os02g41580 // LOC_Os11g22350 // LOC_Os04g01310 // LOC_Os03g03410 // LOC_Os10g30540 // LOC_Os01g54700 // LOC_Os09g39650 // LOC_Os12g27520 // LOC_Os03g17980 // LOC_Os03g53800 // LOC_Os07g43570 // LOC_Os02g48360 // LOC_Os06g05359 // LOC_Os06g10790 // LOC_Os01g04450 // LOC_Os01g62500 // LOC_Os07g48760 // LOC_Os08g10260 // LOC_Os07g35300 // LOC_Os07g48310 // LOC_Os06g08790 // LOC_Os11g29990 // LOC_Os07g39520 // LOC_Os05g44340 // LOC_Os04g24510 // LOC_Os11g02240 // LOC_Os02g57700 // LOC_Os07g12900 // LOC_Os07g35140 // LOC_Os04g35240 // LOC_Os07g35004 // LOC_Os03g12520 // LOC_Os01g70410 // LOC_Os07g35540 // LOC_Os01g56330 // LOC_Os12g02200 // LOC_Os02g09100 // LOC_Os01g59580 // LOC_Os05g33570 // LOC_Os10g04290 // LOC_Os05g51190 // LOC_Os03g08550 // LOC_Os01g62950 // LOC_Os01g25710 // LOC_Os01g12430 // LOC_Os03g60810 // LOC_Os01g45450 // LOC_Os01g65650 // LOC_Os01g47470 // LOC_Os10g37500 // LOC_Os02g19750 // LOC_Os07g35790 // LOC_Os09g39620 // LOC_Os02g48080 // LOC_Os07g05740 // LOC_Os11g10710 // LOC_Os01g02840 // LOC_Os01g04480 // LOC_Os03g58790 // LOC_Os04g52630 // LOC_Os09g34160 // LOC_Os03g58800 // LOC_Os01g12290 // LOC_Os07g48730 // LOC_Os06g10520 // LOC_Os01g53640 // LOC_Os03g38710 // LOC_Os09g38910 // LOC_Os11g10280 // LOC_Os10g35040 // LOC_Os01g05620 // LOC_Os01g02390 // LOC_Os02g30540 // LOC_Os12g03650 // LOC_Os09g07730 // LOC_Os01g44110

GO:0004842 F ubiquitin-protein ligase activity 8 778 96 24460 0.015 0.28 //

LOC_Os03g13010 // LOC_Os02g57700 // LOC_Os02g46500 // LOC_Os02g33680 // LOC_Os06g13870 // LOC_Os05g39930 // LOC_Os02g49950 // LOC_Os09g39620

GO:0003993 F acid phosphatase activity 5 778 45 24460 0.019 0.33 //

LOC_Os05g10310 // LOC_Os04g13210 // LOC_Os05g09660 // LOC_Os08g17784 // LOC_Os01g09540

GO:0005509 F calcium ion binding 20 778 381 24460 0.025 0.43 //

LOC_Os01g72530 // LOC_Os09g24580 // LOC_Os09g38910 // LOC_Os01g25820 // LOC_Os06g47640 // LOC_Os01g31690 // LOC_Os05g31620 // LOC_Os09g29600 // LOC_Os01g04330 // LOC_Os04g57350 // LOC_Os03g54100 // LOC_Os03g63310 // LOC_Os02g50060 // LOC_Os01g53294 // LOC_Os10g36960 // LOC_Os06g46950 // LOC_Os03g53660 // LOC_Os10g02250 // LOC_Os09g26660 // LOC_Os09g30490

GO:0016829 F lyase activity 16 778 286 24460 0.026 0.44 //

LOC_Os02g17920 // LOC_Os03g51740 // LOC_Os06g40640 // LOC_Os12g42980 // LOC_Os11g07020 // LOC_Os02g36285 // LOC_Os01g51980 // LOC_Os02g39790 // LOC_Os08g44210 // LOC_Os08g09250 // LOC_Os10g39710 // LOC_Os03g58300 // LOC_Os02g41650 // LOC_Os07g34520 // LOC_Os02g02930 // LOC_Os01g45274

GO:0046527 F glucosyltransferase activity 6 778 69 24460 0.028 0.47 //

LOC_Os06g06560 // LOC_Os03g59340 // LOC_Os01g54620 // LOC_Os07g10770 // LOC_Os05g44210 // LOC_Os05g08370

GO:0003824 F catalytic activity 455 778 13462 24460 0.031 0.5 //

LOC_Os12g04480 // LOC_Os02g33680 // LOC_Os10g01044 // LOC_Os07g48050 // LOC_Os06g28124 // LOC_Os08g44210 // LOC_Os06g08790 // LOC_Os01g40070 // LOC_Os03g02750 // LOC_Os05g22614 // LOC_Os02g48210 // LOC_Os03g29240 // LOC_Os07g31960 // LOC_Os10g40830 // LOC_Os02g50350 // LOC_Os06g48160 // LOC_Os05g48330 // LOC_Os07g35004 // LOC_Os09g37690 // LOC_Os05g08370 // LOC_Os04g57410 // LOC_Os09g26360 // LOC_Os02g40840 // LOC_Os03g53660 // LOC_Os03g07440 // LOC_Os01g41550 // LOC_Os07g41060 // LOC_Os05g04600 // LOC_Os03g35600 // LOC_Os09g36680 // LOC_Os01g02400 // LOC_Os11g03840 // LOC_Os01g02760 // LOC_Os04g52730 // LOC_Os10g30530 // LOC_Os11g04980 // LOC_Os03g57120 // LOC_Os01g22336 // LOC_Os03g59340 // LOC_Os01g16450 // LOC_Os01g53294 // LOC_Os07g32060 // LOC_Os09g26660 // LOC_Os02g17920 // LOC_Os03g55090 // LOC_Os03g21510 // LOC_Os01g65260 // LOC_Os10g36960 // LOC_Os02g57810 // LOC_Os01g51410 // LOC_Os05g01140 // LOC_Os05g15770 // LOC_Os09g39650 // LOC_Os11g37950 // LOC_Os03g53800 // LOC_Os04g24600 // LOC_Os06g10790 // LOC_Os01g04450 // LOC_Os01g03740 // LOC_Os02g02930 // LOC_Os01g70930 // LOC_Os10g01080 // LOC_Os04g12950 // LOC_Os07g48760 // LOC_Os01g55510 // LOC_Os07g48310 // LOC_Os07g32560 // LOC_Os02g16909 // LOC_Os08g35210 // LOC_Os04g46990 // LOC_Os01g22900 // LOC_Os06g48200 // LOC_Os07g46280 // LOC_Os04g24510 // LOC_Os07g02440 // LOC_Os07g35140 // LOC_Os05g32710 // LOC_Os04g35240 // LOC_Os07g46460 // LOC_Os04g41970 // LOC_Os04g46970 // LOC_Os02g09100 // LOC_Os05g15510 // LOC_Os01g59580 // LOC_Os09g30458 // LOC_Os01g15000 // LOC_Os07g47990 // LOC_Os06g03540 // LOC_Os02g50490 // LOC_Os05g09660 // LOC_Os02g49950 // LOC_Os12g23190 // LOC_Os01g12430 // LOC_Os03g18890 // LOC_Os11g43750 // LOC_Os01g45450 // LOC_Os01g57730 // LOC_Os01g47470 // LOC_Os05g38260 // LOC_Os03g53340 // LOC_Os07g32630 // LOC_Os01g65830 // LOC_Os07g05740 // LOC_Os11g10710 // LOC_Os01g62880 // LOC_Os01g02840 // LOC_Os02g39850 // LOC_Os06g06560 // LOC_Os04g52630 // LOC_Os03g58800 // LOC_Os08g39840 // LOC_Os04g55710 // LOC_Os05g48930 // LOC_Os10g35040 // LOC_Os10g34930 // LOC_Os03g17230 // LOC_Os09g07730 // LOC_Os08g41100 // LOC_Os03g63310 // LOC_Os03g16010 // LOC_Os04g42250 // LOC_Os04g57550 // LOC_Os02g48860 // LOC_Os04g58200 // LOC_Os01g65780 // LOC_Os04g40874 // LOC_Os02g36840 // LOC_Os04g13210 // LOC_Os04g39980 // LOC_Os01g43851 // LOC_Os01g61380 // LOC_Os01g03950 // LOC_Os01g70170 // LOC_Os05g11810 // LOC_Os07g48730 // LOC_Os01g51140 // LOC_Os09g36350 // LOC_Os03g22780 // LOC_Os03g32314 // LOC_Os03g57640 // LOC_Os11g36160 // LOC_Os01g27230 // LOC_Os07g10770 // LOC_Os06g47910 // LOC_Os08g38900 // LOC_Os03g06330 // LOC_Os10g30410 // LOC_Os09g34230 // LOC_Os07g46350 // LOC_Os11g10750 // LOC_Os06g46710 // LOC_Os01g02770 // LOC_Os08g32170 // LOC_Os01g57560 // LOC_Os11g22350 // LOC_Os12g05110 // LOC_Os07g33600 // LOC_Os03g59350 // LOC_Os01g59000 // LOC_Os01g31370 // LOC_Os02g46910 // LOC_Os02g36285 // LOC_Os06g40818 // LOC_Os04g47720 // LOC_Os03g44740 // LOC_Os08g16910 // LOC_Os08g35440 // LOC_Os03g49600 // LOC_Os06g46920 // LOC_Os01g71474 // LOC_Os02g17534 // LOC_Os01g22980 // LOC_Os12g18560 // LOC_Os03g44420 // LOC_Os04g57350 // LOC_Os05g33400 // LOC_Os02g49140 // LOC_Os03g38710 // LOC_Os09g29404 // LOC_Os10g08474 // LOC_Os12g15530 // LOC_Os01g03730 // LOC_Os05g10310 // LOC_Os10g35070 // LOC_Os07g34520 // LOC_Os06g22440 // LOC_Os02g46500 // LOC_Os09g32080 // LOC_Os04g59200 // LOC_Os01g52710 // LOC_Os08g33720 // LOC_Os01g56330 // LOC_Os04g57850 // LOC_Os03g03034 // LOC_Os05g33570 // LOC_Os11g32030 // LOC_Os04g36720 // LOC_Os03g13010 // LOC_Os01g25820 // LOC_Os01g65650 // LOC_Os04g25440 // LOC_Os04g20164 // LOC_Os11g37890 // LOC_Os05g41180 // LOC_Os03g18010 // LOC_Os01g27240 // LOC_Os04g12960 // LOC_Os03g13570 // LOC_Os05g31140 // LOC_Os05g03640 // LOC_Os12g27830 // LOC_Os10g39700 // LOC_Os04g40460 // LOC_Os05g35470 // LOC_Os12g25690 // LOC_Os01g43160 // LOC_Os05g23260 // LOC_Os01g52530 // LOC_Os05g39930 // LOC_Os06g46960 // LOC_Os01g02390 // LOC_Os04g38880 // LOC_Os02g30540 // LOC_Os12g03650 // LOC_Os07g34510 // LOC_Os07g44130 // LOC_Os11g08610 // LOC_Os03g17700 // LOC_Os01g68870 // LOC_Os11g09280 // LOC_Os01g53430 // LOC_Os01g42690 // LOC_Os08g17784 // LOC_Os04g58040 // LOC_Os05g12450 // LOC_Os04g39900 // LOC_Os01g13690 // LOC_Os05g45090 // LOC_Os03g55070 // LOC_Os07g42440 // LOC_Os10g38110 // LOC_Os05g13970 // LOC_Os05g06710 // LOC_Os02g47020 // LOC_Os06g03970 // LOC_Os12g12514 // LOC_Os08g39100 // LOC_Os07g44780 // LOC_Os01g04230 // LOC_Os01g64170 // LOC_Os07g23570 // LOC_Os01g52750 // LOC_Os03g15270 // LOC_Os07g18120 // LOC_Os03g12110 // LOC_Os06g17090 // LOC_Os07g44140 // LOC_Os02g37590 // LOC_Os04g51460 // LOC_Os08g32960 // LOC_Os01g47510 // LOC_Os04g51660 // LOC_Os02g34600 // LOC_Os06g11720 // LOC_Os07g25430 // LOC_Os01g70130 // LOC_Os02g10120 // LOC_Os06g28550 // LOC_Os01g39830 // LOC_Os10g39680 // LOC_Os04g53810 // LOC_Os08g33710 // LOC_Os09g12660 // LOC_Os11g01530 // LOC_Os01g71810 // LOC_Os01g50410 // LOC_Os06g11090 // LOC_Os04g58390 // LOC_Os04g39880 // LOC_Os08g38910 // LOC_Os02g41580 // LOC_Os04g01310 // LOC_Os12g07150 // LOC_Os02g05744 // LOC_Os03g03410 // LOC_Os10g30540 // LOC_Os06g13870 // LOC_Os04g38560 // LOC_Os01g54270 // LOC_Os03g17980 // LOC_Os02g12890 // LOC_Os07g01540 // LOC_Os07g43570 // LOC_Os08g01940 // LOC_Os12g38920 // LOC_Os06g13470 // LOC_Os03g12890 // LOC_Os07g35300 // LOC_Os03g56820 // LOC_Os04g40630 // LOC_Os03g18970 // LOC_Os12g42980 // LOC_Os08g04630 // LOC_Os06g03830 // LOC_Os09g20440 // LOC_Os01g54370 // LOC_Os03g17850 // LOC_Os03g49610 // LOC_Os02g57700 // LOC_Os04g20400 // LOC_Os03g12520 // LOC_Os07g35540 // LOC_Os11g37970 // LOC_Os05g51190 // LOC_Os01g18744 // LOC_Os03g08550 // LOC_Os03g51740 // LOC_Os04g59190 // LOC_Os01g09220 // LOC_Os03g63330 // LOC_Os09g11500 // LOC_Os01g60480 // LOC_Os06g25010 // LOC_Os03g12140 // LOC_Os06g49810 // LOC_Os09g39620 // LOC_Os07g48370 // LOC_Os03g16260 // LOC_Os09g18360 // LOC_Os01g54620 // LOC_Os06g10520 // LOC_Os11g18194 // LOC_Os03g12660 // LOC_Os01g39860 // LOC_Os01g51980 // LOC_Os06g51050 // LOC_Os09g38910 // LOC_Os11g08100 // LOC_Os01g04480 // LOC_Os04g14680 // LOC_Os12g13120 // LOC_Os11g10490 // LOC_Os01g44110 // LOC_Os01g62500 // LOC_Os11g05380 // LOC_Os01g53330 // LOC_Os12g24050 // LOC_Os02g39490 // LOC_Os08g43300 // LOC_Os05g07090 // LOC_Os02g46650 // LOC_Os08g09250 // LOC_Os10g39710 // LOC_Os09g31270 // LOC_Os01g20206 // LOC_Os02g39790 // LOC_Os03g58300 // LOC_Os07g29330 // LOC_Os03g32470 // LOC_Os01g43700 // LOC_Os05g49120 // LOC_Os01g02790 // LOC_Os06g13110 // LOC_Os01g45274 // LOC_Os04g14690 // LOC_Os04g39930 // LOC_Os03g13540 // LOC_Os06g40640 // LOC_Os05g42210 // LOC_Os10g02250 // LOC_Os08g41340 // LOC_Os03g24990 // LOC_Os03g51440 // LOC_Os06g05230 // LOC_Os04g56970 // LOC_Os08g34050 // LOC_Os01g61760 // LOC_Os06g44180 // LOC_Os12g41410 // LOC_Os04g56580 // LOC_Os06g12280 // LOC_Os01g59150 // LOC_Os01g18670 // LOC_Os11g42390 // LOC_Os09g34960 // LOC_Os07g36544 // LOC_Os05g43830 // LOC_Os03g49440 // LOC_Os02g47470 // LOC_Os08g41630 // LOC_Os01g54700 // LOC_Os01g08440 // LOC_Os01g61610 // LOC_Os12g27520 // LOC_Os09g29600 // LOC_Os01g67540 // LOC_Os11g07020 // LOC_Os03g18060 // LOC_Os09g23084 // LOC_Os02g48360 // LOC_Os04g51400 // LOC_Os02g14630 // LOC_Os09g33690 // LOC_Os01g51540 // LOC_Os04g51930 // LOC_Os07g46846 // LOC_Os05g41440 // LOC_Os08g01490 // LOC_Os09g28230 // LOC_Os12g01530 // LOC_Os07g39520 // LOC_Os06g51060 // LOC_Os05g44340 // LOC_Os10g39140 // LOC_Os11g02240 // LOC_Os02g51370 // LOC_Os07g12900 // LOC_Os06g46284 // LOC_Os01g70410 // LOC_Os05g50570 // LOC_Os10g41550 // LOC_Os12g07810 // LOC_Os12g02200 // LOC_Os11g40360 // LOC_Os01g68020 // LOC_Os05g41590 // LOC_Os04g41960 // LOC_Os01g62950 // LOC_Os05g44210 // LOC_Os06g46940 // LOC_Os03g60810 // LOC_Os04g22660 // LOC_Os10g37500 // LOC_Os02g15230 // LOC_Os08g41090 // LOC_Os02g08100 // LOC_Os02g07060 // LOC_Os02g48080 // LOC_Os07g35790 // LOC_Os12g39360 // LOC_Os01g09540 // LOC_Os03g58790 // LOC_Os01g12290 // LOC_Os03g25790 // LOC_Os04g47360 // LOC_Os01g71840 // LOC_Os01g53640 // LOC_Os10g37340 // LOC_Os08g10244 // LOC_Os02g41650 // LOC_Os11g10280 // LOC_Os08g06100 // LOC_Os06g38970 // LOC_Os05g32380 // LOC_Os01g06740

GO:0008194 F UDP-glycosyltransferase activity 7 778 93 24460 0.035 0.55 //

LOC_Os03g59340 // LOC_Os01g54620 // LOC_Os05g44210 // LOC_Os07g10770 // LOC_Os04g58040 // LOC_Os03g17850 // LOC_Os05g08370

GO:0016614 F oxidoreductase activity, acting on CH-OH group of donors 8 778 116 24460 0.038 0.59 //

LOC_Os12g25690 // LOC_Os02g40840 // LOC_Os05g41590 // LOC_Os03g55070 // LOC_Os01g61380 // LOC_Os01g39830 // LOC_Os08g33720 // LOC_Os04g40874

GO:0005507 F copper ion binding 9 778 139 24460 0.04 0.61 //

LOC_Os08g37660 // LOC_Os02g43660 // LOC_Os02g06480 // LOC_Os03g55120 // LOC_Os12g15530 // LOC_Os07g02200 // LOC_Os07g38290 // LOC_Os08g17160 // LOC_Os04g20164

GO:0035251 F UDP-glucosyltransferase activity 5 778 59 24460 0.047 0.7 //

LOC_Os01g54620 // LOC_Os05g44210 // LOC_Os05g08370 // LOC_Os07g10770 // LOC_Os03g59340

GO:0031224 C intrinsic to membrane 41 778 748 24460 0.00086 0.076 //

LOC_Os01g62500 // LOC_Os08g43320 // LOC_Os01g08020 // LOC_Os01g25820 // LOC_Os07g34006 // LOC_Os09g31478 // LOC_Os08g17370 // LOC_Os05g43650 // LOC_Os01g65380 // LOC_Os01g53294 // LOC_Os08g35210 // LOC_Os02g49140 // LOC_Os09g26660 // LOC_Os04g35540 // LOC_Os04g13210 // LOC_Os01g18670 // LOC_Os04g10800 // LOC_Os03g17790 // LOC_Os03g18540 // LOC_Os04g14690 // LOC_Os09g37690 // LOC_Os07g33954 // LOC_Os02g49630 // LOC_Os01g28840 // LOC_Os01g65880 // LOC_Os07g12900 // LOC_Os10g30790 // LOC_Os08g01140 // LOC_Os06g35930 // LOC_Os01g74450 // LOC_Os03g38730 // LOC_Os05g04600 // LOC_Os08g01940 // LOC_Os09g36930 // LOC_Os05g04700 // LOC_Os02g02540 // LOC_Os01g43460 // LOC_Os06g45990 // LOC_Os04g36720 // LOC_Os06g44250 // LOC_Os12g02840

GO:0016020 C membrane 98 778 2185 24460 0.0005 0.076 //

LOC_Os07g34510 // LOC_Os01g63870 // LOC_Os09g31478 // LOC_Os08g17370 // LOC_Os04g58040 // LOC_Os01g65380 // LOC_Os12g33300 // LOC_Os02g50820 // LOC_Os04g13210 // LOC_Os03g17790 // LOC_Os10g42299 // LOC_Os09g37690 // LOC_Os05g08370 // LOC_Os07g34006 // LOC_Os01g43460 // LOC_Os01g28840 // LOC_Os12g40920 // LOC_Os09g26160 // LOC_Os01g58910 // LOC_Os05g02060 // LOC_Os05g04600 // LOC_Os02g06480 // LOC_Os06g05230 // LOC_Os07g10770 // LOC_Os11g22350 // LOC_Os06g45990 // LOC_Os07g25430 // LOC_Os08g43320 // LOC_Os03g57120 // LOC_Os03g59340 // LOC_Os01g53294 // LOC_Os09g26660 // LOC_Os09g25784 // LOC_Os01g59150 // LOC_Os01g31690 // LOC_Os09g36930 // LOC_Os01g18670 // LOC_Os01g08020 // LOC_Os02g49630 // LOC_Os07g37550 // LOC_Os12g23200 // LOC_Os05g32630 // LOC_Os09g25770 // LOC_Os10g30790 // LOC_Os08g01140 // LOC_Os09g26144 // LOC_Os01g74450 // LOC_Os03g18060 // LOC_Os01g70180 // LOC_Os12g03360 // LOC_Os08g01940 // LOC_Os05g04700 // LOC_Os12g38920 // LOC_Os05g14240 // LOC_Os01g62500 // LOC_Os02g17534 // LOC_Os05g35650 // LOC_Os05g43650 // LOC_Os03g11510 // LOC_Os08g35210 // LOC_Os02g49140 // LOC_Os02g55970 // LOC_Os01g67630 // LOC_Os04g10800 // LOC_Os03g39610 // LOC_Os07g37100 // LOC_Os03g17850 // LOC_Os03g42830 // LOC_Os07g12900 // LOC_Os04g43070 // LOC_Os07g01214 // LOC_Os01g65110 // LOC_Os03g38730 // LOC_Os03g41070 // LOC_Os02g02540 // LOC_Os02g48570 // LOC_Os04g36720 // LOC_Os06g44250 // LOC_Os04g35540 // LOC_Os01g25820 // LOC_Os02g45100 // LOC_Os05g45370 // LOC_Os01g41710 // LOC_Os12g25160 // LOC_Os10g21236 // LOC_Os03g18540 // LOC_Os04g14690 // LOC_Os07g33954 // LOC_Os01g65880 // LOC_Os07g05480 // LOC_Os05g23260 // LOC_Os01g54620 // LOC_Os06g35930 // LOC_Os03g08900 // LOC_Os12g41890 // LOC_Os03g54100 // LOC_Os01g70190 // LOC_Os12g02840

GO:0016021 C integral to membrane 39 778 727 24460 0.0016 0.097 //

LOC_Os01g62500 // LOC_Os08g43320 // LOC_Os01g08020 // LOC_Os01g25820 // LOC_Os07g34006 // LOC_Os09g31478 // LOC_Os08g17370 // LOC_Os05g43650 // LOC_Os01g65380 // LOC_Os01g53294 // LOC_Os08g35210 // LOC_Os02g49140 // LOC_Os09g26660 // LOC_Os04g35540 // LOC_Os04g13210 // LOC_Os01g18670 // LOC_Os04g10800 // LOC_Os03g17790 // LOC_Os03g18540 // LOC_Os07g33954 // LOC_Os02g49630 // LOC_Os01g28840 // LOC_Os01g65880 // LOC_Os07g12900 // LOC_Os10g30790 // LOC_Os08g01140 // LOC_Os06g35930 // LOC_Os01g74450 // LOC_Os03g38730 // LOC_Os05g04600 // LOC_Os08g01940 // LOC_Os09g36930 // LOC_Os05g04700 // LOC_Os02g02540 // LOC_Os01g43460 // LOC_Os06g45990 // LOC_Os04g36720 // LOC_Os06g44250 // LOC_Os12g02840

GO:0000151 C ubiquitin ligase complex 9 778 87 24460 0.0029 0.13 //

LOC_Os03g13010 // LOC_Os02g57700 // LOC_Os02g46500 // LOC_Os02g33680 // LOC_Os01g50980 // LOC_Os06g13870 // LOC_Os05g39930 // LOC_Os02g49950 // LOC_Os09g39620

GO:0044425 C membrane part 47 778 1006 24460 0.0074 0.26 //

LOC_Os01g62500 // LOC_Os07g25430 // LOC_Os08g43320 // LOC_Os01g08020 // LOC_Os01g25820 // LOC_Os07g34006 // LOC_Os09g31478 // LOC_Os08g17370 // LOC_Os05g43650 // LOC_Os01g65380 // LOC_Os01g53294 // LOC_Os08g35210 // LOC_Os02g49140 // LOC_Os09g26660 // LOC_Os04g35540 // LOC_Os02g55970 // LOC_Os01g31690 // LOC_Os04g13210 // LOC_Os01g18670 // LOC_Os04g10800 // LOC_Os10g21236 // LOC_Os03g17790 // LOC_Os03g18540 // LOC_Os04g14690 // LOC_Os09g37690 // LOC_Os07g33954 // LOC_Os02g49630 // LOC_Os01g28840 // LOC_Os12g23200 // LOC_Os01g65880 // LOC_Os07g12900 // LOC_Os10g30790 // LOC_Os07g05480 // LOC_Os08g01140 // LOC_Os06g35930 // LOC_Os01g74450 // LOC_Os03g38730 // LOC_Os05g04600 // LOC_Os08g01940 // LOC_Os09g36930 // LOC_Os05g04700 // LOC_Os02g02540 // LOC_Os01g43460 // LOC_Os06g45990 // LOC_Os04g36720 // LOC_Os06g44250 // LOC_Os12g02840

GO:0005618 C cell wall 7 778 82 24460 0.02 0.59 //

LOC_Os09g26360 // LOC_Os03g13570 // LOC_Os06g48200 // LOC_Os06g48160 // LOC_Os04g38560 // LOC_Os02g46910 // LOC_Os04g51460
