# Supplementary material for: OsbZIP81, A Homologue of Arabidopsis VIP1, May Positively Regulate JA Levels by Directly Targetting the Genes in JA Signaling and Metabolism Pathway in Rice
Source: Int J Mol Sci. 2019 May 13;20(9):2360. doi: 10.3390/ijms20092360 (PMC6539606; doi:10.3390/ijms20092360)
Supplement: Supplementary file 1 [file ijms-20-02360-s001.zip › Supplementary Table 1.doc]

**Supplemental Table 1.** Identified motifs in ChIP-Seq experiment with MEME online software

| NO. | Motif | E-value | Sites | Width |
| --- | --- | --- | --- | --- |
| 1 |  | 8.4e-177 | 805 | 8 |
| 2 |  | 2.4e-137 | 277 | 12 |
| 3 |  | 1.2e-057 | 106 | 12 |
| 4 |  | 8.6e-021 | 57 | 12 |
| 5 |  | 8.4e-008 | 37 | 11 |
| 6 |  | 7.4e-008 | 54 | 11 |
| 7 |  | 5.0e-007 | 24 | 12 |
| 8 |  | 3.8e-010 | 57 | 12 |
| 9 |  | 1.6e-004 | 39 | 12 |
| 10 |  | 9.5e-006 | 119 | 10 |
| 11 |  | 3.2e-004 | 45 | 12 |
| 12 |  | 2.5e-002 | 59 | 8 |
| 13 |  | 4.6e-002 | 37 | 12 |
| 14 |  | 3.1e-002 | 45 | 11 |
| 15 |  | 5.2e-002 | 87 | 11 |
