# Supplementary material for: OsbZIP81, A Homologue of Arabidopsis VIP1, May Positively Regulate JA Levels by Directly Targetting the Genes in JA Signaling and Metabolism Pathway in Rice
Source: Int J Mol Sci. 2019 May 13;20(9):2360. doi: 10.3390/ijms20092360 (PMC6539606; doi:10.3390/ijms20092360)
Supplement: Supplementary file 1 [file ijms-20-02360-s001.zip › Supplementary Table 2.doc]

**Supplemental Table 2.** Identified motifs in RDSA experiment with DREME online software

| NO. | Motif | E-value | Width |
| --- | --- | --- | --- |
| 1 |  | 6.3e-023 | 5 |
| 2 |  | 8.4e-005 | 6 |
| 3 |  | 7.6e-003 | 8 |
