# Supplementary material for: OsbZIP81, A Homologue of Arabidopsis VIP1, May Positively Regulate JA Levels by Directly Targetting the Genes in JA Signaling and Metabolism Pathway in Rice
Source: Int J Mol Sci. 2019 May 13;20(9):2360. doi: 10.3390/ijms20092360 (PMC6539606; doi:10.3390/ijms20092360)
Supplement: Supplementary file 1 [file ijms-20-02360-s001.zip › Supplementary Table 3.doc]

**Supplemental Table 3.** Summary of RNA-Seq data collected from the rice shoots of the ZH11, OX-OsbZIP81.1, OX-OsbZIP81.2 and osbzip81 under normal condition, each group contains three biological repeats.

| **Sample** | **Raw reads** | **Clean reads** | **Total mapped** | **Unique mapped** |
| --- | --- | --- | --- | --- |
| ZH11-1 | 77189512 | 75944310 | 71453700 (94.09%) | 69777211 (91.88%) |
| ZH11-2 | 71606854 | 70436190 | 66321136 (94.16%) | 64775847 (91.96%) |
| ZH11-3 | 81620872 | 80476688 | 75812907 (94.2%) | 74084172 (92.06%) |
| OsbZIP81.1ox-1 | 69531678 | 68562684 | 59573766 (86.89%) | 58203506 (84.89%) |
| OsbZIP81.1ox-3 | 73725336 | 72701612 | 64084254 (88.15%) | 62567825 (86.06%) |
| OsbZIP81.1ox-5 | 63133316 | 62225064 | 54950549 (88.31%) | 53668469 (86.25%) |
| OsbZIP81.2ox-2 | 67307620 | 66520854 | 57308966 (86.15%) | 56006722 (84.19%) |
| OsbZIP81.2ox-3 | 60998792 | 59844668 | 52562014 (87.83%) | 51320301 (85.76%) |
| OsbZIP81.2ox-4 | 64952358 | 64088420 | 54014533 (84.28%) | 52801182 (82.39%) |
