# Supplementary material for: OsbZIP81, A Homologue of Arabidopsis VIP1, May Positively Regulate JA Levels by Directly Targetting the Genes in JA Signaling and Metabolism Pathway in Rice
Source: Int J Mol Sci. 2019 May 13;20(9):2360. doi: 10.3390/ijms20092360 (PMC6539606; doi:10.3390/ijms20092360)
Supplement: Supplementary file 1 [file ijms-20-02360-s001.zip › Supplementary Table 4.doc]

**Supplemental Table S4.** **List of clones screened by yeast two-hybrid assay**

| **Gene ID** | **Annotation** | **Clone Numbers** |
| --- | --- | --- |
| LOC_Os01g09010 | transferase family protein, putative, expressed | 26 |
| LOC_Os12g41250 | methylcrotonoyl-CoA carboxylase subunit alpha, mitochondrial precursor, putative, expressed | 18 |
| LOC_Os12g36830 | pathogenesis-related Bet v I family protein, putative, expressed (RSOsPR10) | 11 |
| LOC_Os11g10480 | dehydrogenase, putative, expressed (Adh1) | 10 |
| LOC_Os09g30418 | heat shock protein, putative, expressed | 8 |
| LOC_Os12g36880 | pathogenesis-related Bet v I family protein, putative, expressed (PBZ1) | 6 |
| LOC_Os08g39500 | 60S ribosomal protein L31, putative, expressed | 5 |
| LOC_Os04g48310 | RING-H2 finger protein, putative, expressed | 4 |
| LOC_Os11g05860 | harpin-induced protein 1 domain containing protein, expressed | 4 |
| LOC_Os03g11614 | OsMADS1 - MADS-box family gene with MIKCc type-box (OsMADS1) | 3 |
| LOC_Os06g09870 | retrotransposon protein, putative, unclassified, expressed | 3 |
| LOC_Os03g60720 | expansin precursor, putative, expressed | 3 |
| LOC_Os01g57968 | expressed protein | 3 |
| LOC_Os03g12890 | aminotransferase domain containing protein, putative, expressed | 3 |
| LOC_Os12g25090 | expressed protein (PR6) | 1 |
| LOC_Os12g33944 | ELF protein, expressed | 1 |
| LOC_Os05g02310 | soluble inorganic pyrophosphatase, putative, expressed | 1 |
| LOC_Os09g32810 | ribulose-phosphate 3-epimerase, putative, expressed | 1 |
| LOC_Os03g46060 | thaumatin family domain containing protein, expressed | 1 |
| LOC_Os06g09870 | retrotransposon protein, putative, unclassified, expressed | 1 |
| LOC_Os03g58070 | frigida, putative, expressed | 1 |
| LOC_Os03g63740 | NFD2, putative, expressed | 1 |
| LOC_Os02g07160 | glyoxalase family protein, putative, expressed | 1 |
| LOC_Os02g07150 | urease accessory protein F, putative, expressed | 1 |
| LOC_Os04g55720 | D-3-phosphoglycerate dehydrogenase, chloroplast precursor, putative, expressed | 1 |
| LOC_Os11g02379 | LTPL6 - Protease inhibitor/seed storage | 1 |
| LOC_Os12g08564 | retrotransposon protein, putative, unclassified, expressed | 1 |
| LOC_Os10g03850 | OsFBX352 - F-box domain containing protein, expressed (OsFbx352) | 1 |
| LOC_Os08g39694 | cytochrome P450, putative, expressed | 1 |
| LOC_Os06g22919 | DEFL9 - Defensin and Defensin-like DEFL family, expressed | 1 |
| LOC_Os12g36850 | pathogenesis-related Bet v I family protein, putative, expressed (OsPR10b) | 1 |
| LOC_Os01g09990 | helix-loop-helix DNA-binding domain containing protein, expressed (DPF) | 1 |
| LOC_Os03g38000 | 40S ribosomal protein S3-1, putative, expressed | 1 |
| LOC_Os02g03100 | oxidoreductase, aldo/keto reductase family protein, putative, expressed | 1 |
| LOC_Os07g46852 | sex determination protein tasselseed-2, putative, expressed | 1 |
| LOC_Os03g08320 | ZIM domain containing protein, putative, expressed | 1 |
| LOC_Os02g56140 | helix-loop-helix DNA-binding domain containing protein, expressed | 1 |
| LOC_Os09g10930 | gamma response I protein, putative, expressed | 1 |
| LOC_Os02g06640 | ubiquitin family protein, putative, expressed | 1 |
| LOC_Os10g40730 | expansin precursor, putative, expressed | 1 |
| LOC_Os03g02750 | OsSub25 - Putative Subtilisin homologue, expressed | 1 |
| LOC_Os02g57750 | protein binding protein, putative, expressed | 1 |

This experiment was performed two times getting 154 clones in total
