# Supplementary material for: OsbZIP81, A Homologue of Arabidopsis VIP1, May Positively Regulate JA Levels by Directly Targetting the Genes in JA Signaling and Metabolism Pathway in Rice
Source: Int J Mol Sci. 2019 May 13;20(9):2360. doi: 10.3390/ijms20092360 (PMC6539606; doi:10.3390/ijms20092360)
Supplement: Supplementary file 1 [file ijms-20-02360-s001.zip › Supplementary Table 5.doc]

**Supplemental Table S5.** Partial genes related to plant disease response.

| **Gene ID** | **Reported Name** | **ChIP-Seq (OsbZIP81.1ox)** | **RNA-Seq (OsbZIP81.1ox)** | **RNA-Seq (OsbZIP81.2ox)** | **Y2H Screening** |
| --- | --- | --- | --- | --- | --- |
| LOC_Os01g27230 |  | **+** | **+** | **+** | **-** |
| LOC_Os01g27240 |  | **+** | **+** | **-** | **-** |
| LOC_Os02g10120 | OsLOX5 | **+** | **+** | **+** | **-** |
| LOC_Os03g32314 | OsAOC | **+** | **+** | **+** | **-** |
| LOC_Os05g07090 |  | **+** | **+** | **-** | **-** |
| LOC_Os08g39840 | OsHI-LOX | **+** | **+** | **+** | **-** |
| LOC_Os12g26290 | OsPIOX | **+** | **+** | **+** | **-** |
| LOC_Os12g36830 | RSOsPR10 | **+** | **+** | **+** | **+** |
| LOC_Os12g36850 |  | **-** | **-** | **+** | **+** |
| LOC_Os12g36880 | PR10a/PBZ1 | **-** | **-** | **+** | **+** |
| LOC_Os01g14590 |  | **+** | **+** | **+** | **-** |
| LOC_Os03g56820 | OsFAH2 | **+** | **+** | **-** | **-** |
| LOC_Os11g45740 | OsJAmyb | **+** | **+** | **+** | **-** |
| LOC_Os03g08330 | OsJAZ10 | **+** | **+** | **+** | **-** |
| LOC_Os12g25090 | PR6 | **-** | **+** | **+** | **+** |
| LOC_Os03g46060 | PR-5 | **+** | **+** | **+** | **+** |

“+” means contained, “-” means not contained.
