# Supplementary material for: OsbZIP81, A Homologue of Arabidopsis VIP1, May Positively Regulate JA Levels by Directly Targetting the Genes in JA Signaling and Metabolism Pathway in Rice
Source: Int J Mol Sci. 2019 May 13;20(9):2360. doi: 10.3390/ijms20092360 (PMC6539606; doi:10.3390/ijms20092360)
Supplement: Supplementary file 1 [file ijms-20-02360-s001.zip › Supplementary Table 6.doc]

**Supplemental Table 6.** **List of the primers used in the study**

| **No.** | **Primer Name** | **Primer sequence** | **Vector** | **Restriction site** | **Use in the study** |
| --- | --- | --- | --- | --- | --- |
| 1 | AD/BD-OsbZIP81.1/81.2-F | GGAATTCATGGACCCGCGATTCCCG | pGADT7/pGBKT7 | *Eco*RI | Yeast Two-Hybrid/ Yeast Two-Hybrid Screening |
| 2 | AD/BD-OsbZIP81.1-R | CCGCTCGAGCATGAAATCCATGGAGTCATTTAG | pGADT7/pGBKT7 | *Xho*I | Yeast Two-Hybrid |
| 3 | AD/BD-OsbZIP81.2-R | CCGCTCGAGACTGATCAGATAGAGCACTA | pGADT7/pGBKT7 | *Xho*I | Yeast Two-Hybrid/ Yeast Two-Hybrid Screening |
| 4 | 1301-OsbZIP81.1/81.2-F | CGGGGTACCATGGACCCGCGATTCCCG | pU1301 | *Kpn*I | Transgenic Rice Construction |
| 5 | 1301-OsbZIP81.1-R | CGCGGATCCTCAACTGATCAGATAGAGCA | pU1301 | *Bam*HI | Transgenic Rice Construction |
| 6 | 1301-OsbZIP81.2-R | CGCGGATCCTCACATGAAATCCATGGAGTCATT | pU1301 | *Bam*HI | Transgenic Rice Construction |
| 7 | U3-OsbZIP81-N-F | GGCACTCCTCAGGTGTGCACCCCC | pYLgRNA-OsU3/LacZ | *Bsa*I | Transgenic Rice Construction |
| 8 | U3-OsbZIP81-N-R | AAACGGGGGTGCACACCTGAGGAG | pYLgRNA-OsU3/LacZ | *Bsa*I | Transgenic Rice Construction |
| 9 | U6a-OsbZIP81-C-F | GCCGAACGGAGGCCAATCGATGAG | pYLgRNA-OsU6a | *Bsa*I | Transgenic Rice Construction |
| 10 | U6a-OsbZIP81-C-R | AAACCTCATCGATTGGCCTCCGTT | pYLgRNA-OsU6a | *Bsa*I | Transgenic Rice Construction |
| 11 | U-F | CTCCGTTTTACCTGTGGAATCG |  |  | Transgenic Rice Construction |
| 12 | gRNA-R | CGGAGGAAAATTCCATCCAC |  |  | Transgenic Rice Construction |
| 13 | SP-ML | GCGGTGTCATCTATGTTACTAG |  |  | Transgenic Rice Construction |
| 14 | SP-R | GGGCTGTATCTACGTTATTGAAG |  |  | Transgenic Rice Construction |
| 15 | 6P1-OsbZIP81.1/81.2-F | CGGGATCCATGGACCCGCGATTCCCG | pGEX-6P1 | *Bam*HI | Western blot and Pull-down |
| 16 | 6P1-OsbZIP81.1-R | CCGCTCGAGCATGAAATCCATGGAGTCATTTAG | pGEX-6P1 | *Xho*I | Western blot and Pull-down |
| 17 | 6P1-OsbZIP81.2-R | CCGCTCGAGACTGATCAGATAGAGCACTA | pGEX-6P1 | *Xho*I | Western blot and Pull-down |
| 18 | AD-OsbZIP84-F | TCCCCCGGGTATGGACGGGCGGTTCCCGCTG | pGADT7/pGBKT7 | *Xma*I | Yeast Two-Hybrid |
| 19 | AD-OsbZIP84-R | CGGGATCCCTCACATGAAATCCATGGAGTCG | pGADT7/pGBKT7 | *Bam*HI | Yeast Two-Hybrid |
| 20 | GFP-OsbZIP81.1/81.2-F | GCTCTAGAATGGACCCGCGATTCCCG | pM999-GFP | *Xho*I | Subcellular Localization |
| 21 | GFP-OsbZIP81.1-R | GCTCTAGACATGAAATCCATGGAGTCATTTAG | pM999-GFP | *Xho*I | Subcellular Localization |
| 22 | GFP-OsbZIP81.2-R | GCTCTAGAACTGATCAGATAGAGCACTA | pM999-GFP | *Xho*I | Subcellular Localization |
| 23 | BiFC-OsbZIP81.1/81.2-F | CGGGATCCATGGACCCGCGATTCCCG | pSPYCE(M)/pSPYNE173 | *Bam*HI | BiFC |
| 24 | BiFC-OsbZIP81.1-R | GGGGTACCCATGAAATCCATGGAGTCATT | pSPYCE(M)/pSPYNE173 | *Kpn*I | BiFC |
| 25 | BiFC-OsbZIP81.2-R | GGGGTACCACTGATCAGATAGAGCACTA | pSPYCE(M)/pSPYNE173 | *Kpn*I | BiFC |
| 26 | BiFC-OsbZIP84-F | CGGGATCCATGGACGGGCGGTTCCCGCTG | pSPYCE(M)/pSPYNE173 | *Bam*HI | BiFC |
| 27 | BiFC-OsbZIP84-R | GGGGTACCCATGAAATCCATGGAGTCGTT | pSPYCE(M)/pSPYNE173 | *Kpn*I | BiFC |
| 28 | BiFC-RSOsPR10-F | CGGGATCCATGGCTCCGGTCAGCATCTC | pSPYCE(M)/pSPYNE173 | *Bam*HI | BiFC |
| 29 | BiFC-RSOsPR10-R | GGGGTACCAGCATACTCGGTAGGGTGAGC | pSPYCE(M)/pSPYNE173 | *Kpn*I | BiFC |
| 30 | BiFC-PBZ1-F | CGGGATCCATGGCTCCGGCCTGCGTC | pSPYCE(M)/pSPYNE173 | *Bam*HI | BiFC |
| 31 | BiFC-PBZ1-R | GGGGTACCGGCGTATTCGGCAGGGTGAG | pSPYCE(M)/pSPYNE173 | *Kpn*I | BiFC |
| 32 | BiFC-OsMADS1-F | CGGGATCCATGGGGAGGGGGAAGGTGGA | pSPYCE(M)/pSPYNE173 | *Bam*HI | BiFC |
| 33 | BiFC-OsMADS1-R | CCGCTCGAGTATCCAGCCGGATGGGATGTG | pSPYCE(M)/pSPYNE173 | *Kpn*I | BiFC |
| 34 | Actin-rt-F | TGTATGCCAGTGGTCGTACCA |  |  | Real-time PCR |
| 35 | Actin-rt-R | CCAGCAAGGTCGAGACGAA |  |  | Real-time PCR |
| 36 | OsbZIP81.1-rt-F | CTTAAGTGGCCACCAGGGAC |  |  | Real-time PCR |
| 37 | OsbZIP81.1-rt-R | AGTCATTTAGGGTCTGGCCG |  |  | Real-time PCR |
| 38 | OsbZIP81.2-rt-F | CTGGAGAGGAAGGTGCAGAC |  |  | Real-time PCR |
| 39 | OsbZIP81.2-rt-R | AGTAGTTGCAGAGGCACCAC |  |  | Real-time PCR |
| 40 | AD/BD-OsbZIP81.1-378-R | CGGGATCCCGACCCGCTCCTCTTATGC | pGADT7/pGBKT7 | *Bam*HI | Yeast Two-Hybrid |
| 41 | AD/BD-OsbZIP81.1-379-F | GGAATTCATGGATGGGGAGAGCTCG | pGADT7/pGBKT7 | *Eco*RI | Yeast Two-Hybrid |
| 42 | AD/BD-OsbZIP81.1-630-R | CGGGATCCCTGGAGCAGCGTGAGCTGTG | pGADT7/pGBKT7 | *Bam*HI | Yeast Two-Hybrid |
| 43 | AD/BD-OsbZIP81.1-631-F | GGAATTCAGGGACACGTCTGGCCTA | pGADT7/pGBKT7 | *Eco*RI | Yeast Two-Hybrid |
| 44 | 5'PCR primer-AD | TTCCACCCAAGCAGTGGTATCAACGCAGAGTGG |  |  | Yeast Two-Hybrid Screening |
| 45 | 3'PCR primer-AD | GTATCGATGCCCACCCTCTAGAGGCCGAGGCGGCCGACA |  |  | Yeast Two-Hybrid Screening |
| 46 | Hn-F | GATGTAGGAGGGCGTGGATATGTC |  |  | Transgenic Rice Construction |
| 47 | Hn-R | CTTCTACACAGCCATCGGTCCAGA |  |  | Transgenic Rice Construction |
| 48 | pTXB3-RSOsPR10-F | CATGCCATGGCTATGGCTCCGGTCAGCATCTC | pTXB3 | *Nco*I | Western blot and Pull-down |
| 49 | pTXB3-RSOsPR10-R | GGAATTCAGCATACTCGGTAGGGTGAGC | pTXB3 | *Eco*RI | Western blot and Pull-down |
| 50 | pTXB3-PBZ1-F | CATGCCATGGCTATGGCTCCGGCCTGCGTC | pTXB3 | *Nco*I | Western blot and Pull-down |
| 51 | pTXB3-PBZ1-R | GGAATTCGGCGTATTCGGCAGGGTGAG | pTXB3 | *Eco*RI | Western blot and Pull-down |
| 52 | pTXB3-OsMADS1-F | CATGCCATGGCTATGGGGAGGGGGAAGGTGGA | pTXB3 | *Nco*I | Western blot and Pull-down |
| 53 | pTXB3-OsMADS1-R | GGAATTCTATCCAGCCGGATGGGATGTG | pTXB3 | *Eco*RI | Western blot and Pull-down |
| 54 | AD/BD-OsbZIP25-F | CATATGATGAACGGTGGCATTGGC | pGADT7/pGBKT7 | *Nde*I | Yeast Two-Hybrid |
| 55 | AD/BD-OsbZIP25-R | CTCGAGTTCTTTTGATTCATTGTTCTTCGC | pGADT7/pGBKT7 | *Xho*I | Yeast Two-Hybrid |
| 56 | AD/BD-OsbZIP30-F | CATATGATGCAGGAGCCAAAGCACAC | pGADT7/pGBKT7 | *Nde*I | Yeast Two-Hybrid |
| 57 | AD/BD-OsbZIP30-R | CTCGAGGAAGGTGCTGCTGCTTTCAC | pGADT7/pGBKT7 | *Xho*I | Yeast Two-Hybrid |
| 58 | AD/BD-OsbZIP35-F | GAATTCATGGAGGGTGAACCCTCACG | pGADT7/pGBKT7 | *Eco*RI | Yeast Two-Hybrid |
| 59 | AD/BD-OsbZIP35-R | CTCGAGTTGTGGCTGATTATGCTGGATTTG | pGADT7/pGBKT7 | *Xho*I | Yeast Two-Hybrid |
| 60 | AD/BD-OsbZIP36-F | CATATGATGGCAGAGTCCAGAATATTCG | pGADT7/pGBKT7 | *Nde*I | Yeast Two-Hybrid |
| 61 | AD/BD-OsbZIP36-R | GGATCCCGGCTCACTCCTCAGAGC | pGADT7/pGBKT7 | *Bam*HI | Yeast Two-Hybrid |
| 62 | AD/BD-OsbZIP61-F | CATATGATGGCGATGCCGCCCAAG | pGADT7/pGBKT7 | *Nde*I | Yeast Two-Hybrid |
| 63 | AD/BD-OsbZIP61-R | GGATCCGAATGTGCTGTTGCTTTCACCTG | pGADT7/pGBKT7 | *Bam*HI | Yeast Two-Hybrid |
| 64 | AD/BD-OsbZIP68-F | CATATGATGGACGACCCCGGTGCG | pGADT7/pGBKT7 | *Nde*I | Yeast Two-Hybrid |
| 65 | AD/BD-OsbZIP68-R | GGATCCTGCGGATTTCTGTGGCTGTTGTTG | pGADT7/pGBKT7 | *Bam*HI | Yeast Two-Hybrid |
| 66 | AD/BD-OsbZIP75 | GAATTCATGAACAGGGAGAAATCCCCGA | pGADT7/pGBKT7 | *Eco*RI | Yeast Two-Hybrid |
| 67 | AD/BD-OsbZIP75 | CTCGAGGTTGCCGCTGCTTCCTGA | pGADT7/pGBKT7 | *Xho*I | Yeast Two-Hybrid |
| 68 | AD/BD-OsbZIP76 | GAATTCATGGAGGATGATCCTACACGC | pGADT7/pGBKT7 | *Eco*RI | Yeast Two-Hybrid |
| 69 | AD/BD-OsbZIP76 | CTCGAGTGGCTGATATCCCTGGATTTGT | pGADT7/pGBKT7 | *Xho*I | Yeast Two-Hybrid |
| 70 | AD/BD-OsbZIP78 | GAATTCATGAACACCACGTCCGCGAA | pGADT7/pGBKT7 | *Eco*RI | Yeast Two-Hybrid |
| 71 | AD/BD-OsbZIP78 | CTCGAGTTTGGACTCGTGATTCCTCGC | pGADT7/pGBKT7 | *Xho*I | Yeast Two-Hybrid |
| 72 | GAL4DB-OsbZIP81.1-F | CGGGATCCATGGACCCGCGATTCCCG | GAL4DB | *Eco*RI | TF activity |
| 73 | GAL4DB-OsbZIP81.1-R | GGAATTCCATGAAATCCATGGAGTCATTTAG | GAL4DB | *Bam*HI | TF activity |
| 74 | OsbZIP25-rt-F | CCCTGACAACCGAAGTCCAA |  |  | Real-time PCR |
| 75 | OsbZIP25-rt-R | ATGGTATCTGCGTAGCCTGC |  |  | Real-time PCR |
| 76 | OsbZIP30-rt-F | CCCAGTTGCCACCACAATTC |  |  | Real-time PCR |
| 77 | OsbZIP30-rt-R | TGCTGATGTCCAAACCCTGA |  |  | Real-time PCR |
| 78 | OsbZIP35-rt-F | CTGCAGAGCTTGCGAATGTG |  |  | Real-time PCR |
| 79 | OsbZIP35-rt-R | GCAGATGCCCGATTGTTGAG |  |  | Real-time PCR |
| 80 | OsbZIP36-rt-F | TTAGCACGGTCCAACAGCAG |  |  | Real-time PCR |
| 81 | OsbZIP36-rt-R | ATGCCGCCACAATTCCTTTC |  |  | Real-time PCR |
| 82 | OsbZIP61-rt-F | AGCTATCTGACCAACACGCA |  |  | Real-time PCR |
| 83 | OsbZIP61-rt-R | GGTGGGATAGCATCTGGTGG |  |  | Real-time PCR |
| 84 | OsbZIP68-rt-F | GCACAGCTAAGAGATGCCCT |  |  | Real-time PCR |
| 85 | OsbZIP68-rt-R | TCGTCTGCGATGACTGTTGT |  |  | Real-time PCR |
| 86 | OsbZIP75-rt-F | ACAACCAGGCCATGCAATCT |  |  | Real-time PCR |
| 87 | OsbZIP75-rt-R | TCATCTTGAGGTCTCGTGCC |  |  | Real-time PCR |
| 88 | OsbZIP76-rt-F | AGCTCGTGTTGAGTGATCCG |  |  | Real-time PCR |
| 89 | OsbZIP76-rt-R | GAGCCGTCGCCAATTTTTCA |  |  | Real-time PCR |
| 90 | OsbZIP78-rt-F | TGCATTGAACGAGGCACTGA |  |  | Real-time PCR |
| 91 | OsbZIP78-rt-R | CTGACAGCGGAGCTGGATTT |  |  | Real-time PCR |
| 92 | OsbZIP84-rt-F | TCACGCTTCTCCAGAGGGAT |  |  | Real-time PCR |
| 93 | OsbZIP84-rt-R | TCGCTGAACTTCTTCTCGCA |  |  | Real-time PCR |
| 94 | Os01g04330-rt -F | GCCGCGTTCGACGTGTA |  |  | Real-time PCR |
| 95 | Os01g04330-rt -R | CGATCATCCTCTCGCACTCC |  |  | Real-time PCR |
| 96 | Os01g08320-rt -F | CCCCTGGAAGATGTTTGTGGA |  |  | Real-time PCR |
| 97 | Os01g08320-rt -R | GCGGCTCTTGGTGCTAAGTT |  |  | Real-time PCR |
| 98 | Os01g10590-rt -F | CAAACCCAAGCAATCCGACG |  |  | Real-time PCR |
| 99 | Os01g10590-rt -R | CTCTCGTAGCACACCACCTC |  |  | Real-time PCR |
| 100 | Os01g11150-rt -F | CCTCCAGGTGCTAACGAACG |  |  | Real-time PCR |
| 101 | Os01g11150-rt -R | CGACTTCTCGCTGTTCACCA |  |  | Real-time PCR |
| 102 | Os01g11940-rt -F | TGGTGTTCCTGCTGTTCGAG |  |  | Real-time PCR |
| 103 | Os01g11940-rt -R | AGACTCCCTCTGGCAGTTGA |  |  | Real-time PCR |
| 104 | Os01g20980-rt -F | CTCAGCACGCTCTACTACGC |  |  | Real-time PCR |
| 105 | Os01g20980-rt-R | ATGTTGCCGACGGTGAAGTT |  |  | Real-time PCR |
| 106 | Os01g25820-rt-F | CGATGCTCGTTCTGCTCTCA |  |  | Real-time PCR |
| 107 | Os01g25820-rt-R | TAGGTCGTGCGAAATGGGTC |  |  | Real-time PCR |
| 108 | Os01g27230-rt-F | ACTACATGGACTGCCACGAC |  |  | Real-time PCR |
| 109 | Os01g27230-rt-R | TTCTCGGCTCGATCATGTGG |  |  | Real-time PCR |
| 110 | Os01g29150-rt-F | TTCATACCGTTCGGCCTTGG |  |  | Real-time PCR |
| 111 | Os01g29150-rt-R | CTCGAACCGATGCAGGATGA |  |  | Real-time PCR |
| 112 | Os01g54490-rt-F | TTTGCACCAGAGATGCGACA |  |  | Real-time PCR |
| 113 | Os01g54490-rt-R | GGCTTCCCTTTGGCAGTTGA |  |  | Real-time PCR |
| 114 | Os01g55240-rt-F | ATGGGAGGTTCAAGAGCGTG |  |  | Real-time PCR |
| 115 | Os01g55240-rt-R | TGGCAATGGTGCAATCCTCT |  |  | Real-time PCR |
| 116 | Os01g59360-rt-F | ATTGCTGGATGCTTGTCGGA |  |  | Real-time PCR |
| 117 | Os01g59360-rt-R | CTTGGTCCCTTGCTTGGACA |  |  | Real-time PCR |
| 118 | Os01g72530-rt-F | TCGGCATGTACGAGATGGAAG |  |  | Real-time PCR |
| 119 | Os01g72530-rt-R | CGCCGTTGAGATCGAACCT |  |  | Real-time PCR |
| 120 | Os02g10120-rt-F | CGACGAGCGGAACAACGA |  |  | Real-time PCR |
| 121 | Os02g10120-rt-R | ATGGAGATGCTGTTTGGGATG |  |  | Real-time PCR |
| 122 | Os02g11020-rt-F | CACGACACGAGATACTGGGG |  |  | Real-time PCR |
| 123 | Os02g11020-rt-R | TAGCCCGAACGGTATGAACG |  |  | Real-time PCR |
| 124 | Os02g11070-rt-F | GACGACAAGTGCTTCGGTTG |  |  | Real-time PCR |
| 125 | Os02g11070-rt-R | AGCGTCGTGATGTTCGTCTT |  |  | Real-time PCR |
| 126 | Os02g17390-rt-F | GGCTGGATATGGAGTGGCAT |  |  | Real-time PCR |
| 127 | Os02g17390-rt-R | CTGCCGTCCATTCTGTACCA |  |  | Real-time PCR |
| 128 | Os02g30060-rt-F | CAGTTGTTGGCCTTACTGGC |  |  | Real-time PCR |
| 129 | Os02g30060-rt-R | CCAGGAGCAACAGCATTCAC |  |  | Real-time PCR |
| 130 | Os02g36210-rt-F | TCCGACAGTACAGTGCCAAC |  |  | Real-time PCR |
| 131 | Os02g36210-rt-R | GTAAGCTCCTCGCTGCTGAA |  |  | Real-time PCR |
| 132 | Os02g36264-rt-F | CTAGGGATGCGGGTGTTGTT |  |  | Real-time PCR |
| 133 | Os02g36264-rt-R | CATCCCCTCTGCCGTGAAAT |  |  | Real-time PCR |
| 134 | Os02g36510-rt-F | AGACCTCGCTGCACATCAC |  |  | Real-time PCR |
| 135 | Os02g36510-rt-R | CTACCGCTTCTGGTGTACTCG |  |  | Real-time PCR |
| 136 | Os02g43194-rt-F | GTTGAAACAGCGGATCGTCG |  |  | Real-time PCR |
| 137 | Os02g43194-rt-R | CGTGTACTTGCCGTGGTACT |  |  | Real-time PCR |
| 138 | Os02g49720-rt-F | TTCGGCGGCTACAAGATGAG |  |  | Real-time PCR |
| 139 | Os02g49720-rt-R | TTGGTCTGGAGGTAGTTGCG |  |  | Real-time PCR |
| 140 | Os03g32314-rt-F | CGTACCTGACCTACGAGGAG |  |  | Real-time PCR |
| 141 | Os03g32314-rt-R | GGATGCCCTTGAGGTAGAAGG |  |  | Real-time PCR |
| 142 | Os03g49610-rt-F | AACTTCGAGTGGCTGTCAGG |  |  | Real-time PCR |
| 143 | Os03g49610-rt-R | TGAACCAGTTGGCTGAGTCC |  |  | Real-time PCR |
| 144 | Os03g53800-rt-F | CGACAAGTACGACTACGCGA |  |  | Real-time PCR |
| 145 | Os03g53800-rt-R | CTCTTGCACACCGTCTGGAT |  |  | Real-time PCR |
| 146 | Os03g63970-rt-F | GGTGTACCCGGACTTCACG |  |  | Real-time PCR |
| 147 | Os03g63970-rt-R | TGTTGGTTGCAGGTGACGAT |  |  | Real-time PCR |
| 148 | Os04g24430-rt-F | ATTCGGGCTAACGGTCATCG |  |  | Real-time PCR |
| 149 | Os04g24430-rt-R | GTGGAAACCAGAGACACCGT |  |  | Real-time PCR |
| 150 | Os04g32480-rt-F | GATGGCAACCGAGGCAACTA |  |  | Real-time PCR |
| 151 | Os04g32480-rt-R | TACGGACCCAAGCTGGTCAA |  |  | Real-time PCR |
| 152 | Os04g35240-rt-F | GAACCACCCATGGTTCCTGA |  |  | Real-time PCR |
| 153 | Os04g35240-rt-R | AGTAGGTCGGGGCACTGTTA |  |  | Real-time PCR |
| 154 | Os04g39864-rt-F | GAGGGATGGAGCAAACGTGA |  |  | Real-time PCR |
| 155 | Os04g39864-rt-R | GGCCGAGTTCTTGGGGTATC |  |  | Real-time PCR |
| 156 | Os04g47300-rt-F | AGAAGTGGCACAATCACGGT |  |  | Real-time PCR |
| 157 | Os04g47300-rt-R | TGCTGCCACTCTTGTCTACG |  |  | Real-time PCR |
| 158 | Os04g52210-rt-F | TTTCTTCTACTCCCGTGGCG |  |  | Real-time PCR |
| 159 | Os04g52210-rt-R | TATGGGTTCTTGGCTTGCGA |  |  | Real-time PCR |
| 160 | Os05g04170-rt-F | TGCGATTGGAAGAAGTGCCT |  |  | Real-time PCR |
| 161 | Os05g04170-rt-F | TGCGATTGGAAGAAGTGCCT |  |  | Real-time PCR |
| 162 | Os05g04170-rt-R | ACCATCCATCAGCCATGACC |  |  | Real-time PCR |
| 163 | Os05g04170-rt-R | ACCATCCATCAGCCATGACC |  |  | Real-time PCR |
| 164 | Os05g05460-rt-F | CTGCGACATCGACAACAGGA |  |  | Real-time PCR |
| 165 | Os05g05460-rt-R | AGCCTGGCGGATTCAAACTC |  |  | Real-time PCR |
| 166 | Os05g11810-rt-F | CCACCACACCCTGAGTTGAA |  |  | Real-time PCR |
| 167 | Os05g11810-rt-R | AGAAGCCCAGCTACCATCAC |  |  | Real-time PCR |
| 168 | Os05g24780-rt-F | CGGGTGCGCGACATGA |  |  | Real-time PCR |
| 169 | Os05g24780-rt-R | TTAATTACCAGCGGCCATCA |  |  | Real-time PCR |
| 170 | Os05g30280-rt-F | GGGCTGGAGAATGCACTTGA |  |  | Real-time PCR |
| 171 | Os05g30280-rt-R | CACATCATCCAGGGTTCCGT |  |  | Real-time PCR |
| 172 | Os05g39720-rt-F | CTCTTACACGAGCCAGCAGC |  |  | Real-time PCR |
| 173 | Os05g39720-rt-R | CGTCTCTCGGCTCATCCTTG |  |  | Real-time PCR |
| 174 | Os05g44180-rt-F | GAGCCAAGATCAGGCATCCA |  |  | Real-time PCR |
| 175 | Os05g44180-rt-R | CCACGATGTTGAGGTGGTGT |  |  | Real-time PCR |
| 176 | Os06g06300-rt-F | GGGCAAGAGGTGATGTGCTA |  |  | Real-time PCR |
| 177 | Os06g06300-rt-R | TAGAGCTCGGCGAAGTTCCT |  |  | Real-time PCR |
| 178 | Os06g11290-rt-F | AAGGACGAAGGAAGATCGCC |  |  | Real-time PCR |
| 179 | Os06g11290-rt-R | ATCAGCCACCACCTTGTTCC |  |  | Real-time PCR |
| 180 | Os06g15170-rt-F | TTCGGCTCCGGTTTCAAGTG |  |  | Real-time PCR |
| 181 | Os06g15170-rt-R | TCGGGAATGTCCACCGGATA |  |  | Real-time PCR |
| 182 | Os06g24850-rt-F | CATGGCTTCTTGTCAGATGGC |  |  | Real-time PCR |
| 183 | Os06g24850-rt-R | GCATGCGGTCTCCCTCATTA |  |  | Real-time PCR |
| 184 | Os06g45970-rt-F | CAGGAGTTCGGGTACAAGCA |  |  | Real-time PCR |
| 185 | Os06g45970-rt-R | TTAGCTCTTGGCGGCGG |  |  | Real-time PCR |
| 186 | Os06g46284-rt-F | GATCTCTTCCTGGACGACGG |  |  | Real-time PCR |
| 187 | Os06g46284-rt-F | GATCTCTTCCTGGACGACGG |  |  | Real-time PCR |
| 188 | Os06g46284-rt-R | CGCCGCTGAATTTGATCTGG |  |  | Real-time PCR |
| 189 | Os06g46284-rt-R | CGCCGCTGAATTTGATCTGG |  |  | Real-time PCR |
| 190 | Os06g46940-rt-F | AAACGGCAGCATCAAGTTGG |  |  | Real-time PCR |
| 191 | Os06g46940-rt-R | AATGACTACGGGCGGGTTTC |  |  | Real-time PCR |
| 192 | Os06g48850-rt-F | CGAGCAACAAGATCAGGGACA |  |  | Real-time PCR |
| 193 | Os06g48850-rt-R | GTACACCGCGAACGACCC |  |  | Real-time PCR |
| 194 | Os07g03750-rt-F | GTGGTACAACTCGACGGACA |  |  | Real-time PCR |
| 195 | Os07g03750-rt-R | CCAGTAGTTGCAGGCGACG |  |  | Real-time PCR |
| 196 | Os07g26540-rt-F | GAAGAAGATCGGGAGGGTCG |  |  | Real-time PCR |
| 197 | Os07g26540-rt-R | AACTTGCCGTAGTGCTCGAA |  |  | Real-time PCR |
| 198 | Os07g46280-rt-F | GGTATGGACCAACCTGGCAA |  |  | Real-time PCR |
| 199 | Os07g46280-rt-R | GCACCATCGTCTATCGCCTT |  |  | Real-time PCR |
| 200 | Os07g49100-rt-F | TCGTAAGCCGATGGACAACC |  |  | Real-time PCR |
| 201 | Os07g49100-rt-R | CGTCTTCACCTTGTCCGTGA |  |  | Real-time PCR |
| 202 | Os08g12840-rt-F | GTCATCTTCAGAAACGCCGC |  |  | Real-time PCR |
| 203 | Os08g12840-rt-R | GAAATGAGCCTGCTGACACG |  |  | Real-time PCR |
| 204 | Os09g28500-rt-F | GGTTCACGGGGATCAAGTGC |  |  | Real-time PCR |
| 205 | Os09g28500-rt-R | TCGATCTCGCTGTCGTCGAT |  |  | Real-time PCR |
| 206 | Os09g31430-rt-F | ATGGACTGCTTTGAGTGGGG |  |  | Real-time PCR |
| 207 | Os09g31430-rt-R | TAGCTGGACTCCTTGCGGTA |  |  | Real-time PCR |
| 208 | Os10g25290-rt-F | CGACAACGAGCGAACCATAC |  |  | Real-time PCR |
| 209 | Os10g25290-rt-R | CGGCTCATCTTTCACCGGC |  |  | Real-time PCR |
| 210 | Os10g31950-rt-F | TTAACGGAGGTGCAATGGCT |  |  | Real-time PCR |
| 211 | Os10g31950-rt-R | CCCCCATGCCAGAACCTATG |  |  | Real-time PCR |
| 212 | Os11g04560-rt-F | GACGAGCTCTGCTCCGTCG |  |  | Real-time PCR |
| 213 | Os11g04560-rt-R | ATCATCATGCGGGCGAACTC |  |  | Real-time PCR |
| 214 | Os11g10510-rt-F | CGAAACCGAAAAAGGGCCAG |  |  | Real-time PCR |
| 215 | Os11g10510-rt-F | GAAGTTCATCACGCACAGCG |  |  | Real-time PCR |
| 216 | Os11g10510-rt-R | GGGTTCAGGTCCACACCAAT |  |  | Real-time PCR |
| 217 | Os11g10510-rt-R | CTCTCCCCCTTGAGCATGAG |  |  | Real-time PCR |
| 218 | Os11g34460-rt-F | CAAGTTCGCCTGGGGTCATA |  |  | Real-time PCR |
| 219 | Os11g34460-rt-R | AATCCACTCCTCCCCAGTGT |  |  | Real-time PCR |
| 220 | Os12g04480- rt-F | ACTTCAGAATGCGGTAGGGG |  |  | Real-time PCR |
| 221 | Os12g04480- rt-R | GCAAGCTCATAGCCAGGACA |  |  | Real-time PCR |
| 222 | Os12g26290- rt-F | ACAGAATCGACCTTGCTGCC |  |  | Real-time PCR |
| 223 | Os12g26290- rt-R | TTGTCAGATCCTCCCAGCTT |  |  | Real-time PCR |
| 224 | Os12g37320- rt-F | TTCCAAACGCCCGAGATTCA |  |  | Real-time PCR |
| 225 | Os12g37320- rt-R | CGTGTCCCTGACAAGTTGGA |  |  | Real-time PCR |
| 226 | Os11g06170-P1-F | ACCTACCCTATCCTCTCAGCC |  |  | ChIP-qPCR |
| 227 | Os11g06170-P1-R | AATCGGACACGAAAATGCACG |  |  | ChIP-qPCR |
| 228 | Os11g06170-P2-F | TCAGACAAGTATCTTGTGGCTTGTG |  |  | ChIP-qPCR |
| 229 | Os11g06170-P2-R | GGCCCCACGGGTAAGCC |  |  | ChIP-qPCR |
| 230 | Os11g06170-P3-F | CGTGTCTCACAGCTACCCG |  |  | ChIP-qPCR |
| 231 | Os11g06170-P3-R | GGTTGCGAGAAAGCGAGCA |  |  | ChIP-qPCR |
| 232 | Os11g06170-P4-F | TACACCCCAACTCATGCACC |  |  | ChIP-qPCR |
| 233 | Os11g06170-P4-R | GGTCCACGGAAGAACGGAAT |  |  | ChIP-qPCR |
| 234 | Os11g06170-P5-F | CGAGACCCATTACCTACCCG |  |  | ChIP-qPCR |
| 235 | Os11g06170-P5-R | GGGAATCGCGGGTCCATGA |  |  | ChIP-qPCR |
| 236 | Os05g01940-P1-F | TGTGGGTGTGTAATAAAGCAGT |  |  | ChIP-qPCR |
| 237 | Os05g01940-P1-R | TGACTGGCTCAACGTATGGT |  |  | ChIP-qPCR |
| 238 | Os05g01940-P2-F | GCAGTGGGCCAGCTACCAA |  |  | ChIP-qPCR |
| 239 | Os05g01940-P2-R | CCATGTCCGTCCATCGCTGT |  |  | ChIP-qPCR |
| 240 | Os05g01940-P3-F | GGTTTGGTTGACGACGACTTG |  |  | ChIP-qPCR |
| 241 | Os05g01940-P3-R | GGACATCAGCGCAAGCAGTA |  |  | ChIP-qPCR |
| 242 | Os05g01940-P4-F | AGCCTCGGCAACCAACC |  |  | ChIP-qPCR |
| 243 | Os05g01940-P4-R | CGCAAGAAGGGATGGATATTGTG |  |  | ChIP-qPCR |
| 244 | Os10g08230-P1-F | TCGCTGGTTGTGGTCGG |  |  | ChIP-qPCR |
| 245 | Os10g08230-P1-R | CCTCTAGATTTAGCCCCGTCA |  |  | ChIP-qPCR |
| 246 | Os10g08230-P2-F | AGGCCCAGTTTAGCACAGTTT |  |  | ChIP-qPCR |
| 247 | Os10g08230-P2-R | ACCCACGAATATGCCCCATT |  |  | ChIP-qPCR |
| 248 | Os10g08230-P3-F | GTTCCATTCAGCACACGTCG |  |  | ChIP-qPCR |
| 249 | Os10g08230-P3-R | CGAGGGTGAGAGCTTGTTGT |  |  | ChIP-qPCR |
| 250 | Os12g36830-qrt-F | CCAACGGTCCAGATTTGTTGC |  |  | ChIP-qPCR |
| 251 | Os12g36830-qrt-R | GATCCGGTGCAGTTGCTAGT |  |  | ChIP-qPCR |
| 252 | Os09g04680-qrt-F | GGCTGGCTCGATGGCTTTAT |  |  | ChIP-qPCR |
| 253 | Os09g04680-qrt-R | CTCCAACCACCCCACGAAT |  |  | ChIP-qPCR |
| 254 | Os06g12660-qrt-F | ACGGCCACTTCTCCACTGTA |  |  | ChIP-qPCR |
| 255 | Os06g12660-qrt-R | GATACGCGAGTGGGACATGG |  |  | ChIP-qPCR |
| 256 | Os04g52880-qrt-F | CAGCATGGCAGTGAGTCAGAG |  |  | ChIP-qPCR |
| 257 | Os04g52880-qrt-R | AGCTTCATTTCTCTCGTCGGTT |  |  | ChIP-qPCR |
| 258 | Os10g39440-qrt-F | TGGCTGGCTCTACTGCTAAT |  |  | ChIP-qPCR |
| 259 | Os10g39440-qrt-R | AGTGAGAGAGGGTGGTGATCT |  |  | ChIP-qPCR |
| 260 | Os05g03860-qrt-F | GAGCTGTAACTTTCCAAGGGGT |  |  | ChIP-qPCR |
| 261 | Os05g03860-qrt-R | AATGGGAGGTTGGACTGGAC |  |  | ChIP-qPCR |
| 262 | Os06g09900-qrt-F | GAGTGGTGTATGTGGCGGTG |  |  | ChIP-qPCR |
| 263 | Os06g09900-qrt-R | GCACCAATTCCACCTAGCAG |  |  | ChIP-qPCR |
| 264 | Os03g36750-qrt-F | TCAGTAGCCTTAGAAAACAAAAGGA |  |  | ChIP-qPCR |
| 265 | Os03g36750-qrt-R | GTATCTTTCGGTGGGTGGCA |  |  | ChIP-qPCR |
| 266 | Os01g12730-qrt-F | TCATGCTGTTTCTGGCTGGAC |  |  | ChIP-qPCR |
| 267 | Os01g12730-qrt-R | CCAACCAATTCATGCCTGTCTC |  |  | ChIP-qPCR |
| 268 | Os01g72330-qrt-F | ACAACGCATATTGGAGGACGA |  |  | ChIP-qPCR |
| 269 | Os01g72330-qrt-R | TGAGATTCGTGCAAGGGCG |  |  | ChIP-qPCR |
| 270 | Os12g36880-qrt-F | TATATCCGAGGTGGCACAGTGA |  |  | ChIP-qPCR |
| 271 | Os12g36880-qrt-R | TGCTCTGAGATGGGTCTAAACA |  |  | ChIP-qPCR |
| 272 | OVRE-F | TGCGCCTAGCTGGCCCCACAGCGCCTAGCTGGCCCCACAGCGCCTAGCTGGCCCCACAT |  |  | EMSA |
| 273 | OVRE-R | ATGTGGGGCCAGCTAGGCGCTGTGGGGCCAGCTAGGCGCTGTGGGGCCAGCTAGGCGCA |  |  | EMSA |
| 274 | OsbZIP81.1-GAL4DB-F | CGGGATCCATGGACCCGCGATTCCCG | 35S-GAL4DB | *Bam* HI | Dual Luciferase Transcriptional Activity Assay |
| 275 | OsbZIP81.1-GAL4DB-R | GGGGTACCTCACATGAAATCCATGGAGTCATT | 35S-GAL4DB | *Kpn* I | Dual Luciferase Transcriptional Activity Assay |
| 276 | OsbZIP84-GAL4DB-F | CGGGATCCATGGACGGGCGGTTCCCGCTG | 35S-GAL4DB | *Bam* HI | Dual Luciferase Transcriptional Activity Assay |
| 277 | OsbZIP84-GAL4DB-R | GGGGTACCTCACATGAAATCCATGGAGTCG | 35S-GAL4DB | *Kpn* I | Dual Luciferase Transcriptional Activity Assay |
| 278 | OsPIOX-190LUC-F | CCCAAGCTTAGGCTGTAAAGTTTTGGGGCA | 190LUC | *Hind* III | Dual Luciferase Transcriptional Activity Assay |
| 279 | OsPIOX-190LUC-R | CGGGATCCCGTGTCGTCGTTAGTCTCGT | 190LUC | *Bam* HI | Dual Luciferase Transcriptional Activity Assay |
